# The Dunedin study after half a century: reflections on the past, and course for the future

**DOI:** 10.1080/03036758.2022.2114508

**Published:** 2022-09-07

**Authors:** Richie Poulton, Hayley Guiney, Sandhya Ramrakha, Terrie E. Moffitt

**Affiliations:** a Dunedin Multidisciplinary Health and Development Research Unit, Department of Psychology, Division of Sciences, University of Otago, Dunedin, New Zealand; b Department of Psychology and Neuroscience, Duke University, Durham, NC, USA; c SGDP Centre, Kings College London, London, UK

**Keywords:** Lifecourse, multidisciplinary, Dunedin, development, policy

## Abstract

Over the last 50 years Dunedin Study researchers have published more than 1400 peer-reviewed journal articles, books, and reports on many aspects of human health and development. In this 50th anniversary piece we reflect on (i) our historical roots and necessary re-invention through time; (ii) the underpinning principles that have contributed to our success; (iii) some selected examples of high-impact work from the behavioural, oral health, and respiratory domains; (iv) some of the challenges we have encountered over time and how to overcome these; and (vi) review where we see the Study going in the future. We aim to present some of the ‘back story’, which is typically undocumented and oft lost to memory, and thus focus on ‘know-how’. Our hope is to humanise our research, share insights, and to acknowledge the real heroes of the Study – the 1037 Study members, their families and their friends, who have collectively given so much, for so long, in the hope of helping others.

## The study sample

Dunedin Study investigators have studied a representative community cohort of New Zealanders born between 1 April 1972 and 31 March 1973 in the city of Dunedin, located on the South Island of Aotearoa/New Zealand. Perinatal data were available from a larger six-year perinatal health study run by paediatrician Patricia Buckfield between 1968 and 1973 (Buckfield 1978). The Dunedin longitudinal study was formed under Phil Silva’s leadership when those families who were still resident in the greater Dunedin metropolitan three years after their child’s birth (*n* = 1139) were invited to take part in a follow-up study about child development (at ages three and five). Ninety-one percent (*n* = 1037) of those eligible agreed to participate, with families declining being no different from those enrolling in terms of sociodemographic background and birth characteristics. The foundation cohort has been assessed repeatedly at ages 3, 5, 7, 9, 11, 13, 15, 18, 21, 26, 32, 38 and most recently at 45 years, using measures capturing a wide range of psychosocial, physiological and biological outcomes (see Tables 1a and b in the Supplement for a summary of domains assessed/measures applied). Retention has been high with rates above 90% among living Study members at all ages except at 13 years (see Table 2 in the Supplement). The cohort represents the full range of socioeconomic status (SES) in the general population of New Zealand’s South Island (Poulton et al. 2015) and as adults matched the National Health and Nutrition Surveys on key adult indicators, for example BMI, smoking, GP visits, and educational attainment (Poulton et al. 2006; Richmond-Rakerd et al. 2020).

At our most recent age-45 assessment carried out between 2017 and 2019, 94.1% (*n* = 938/997) of living Study members participated. To the best of our knowledge, this is the highest follow-up rate for a study of this design and duration (e.g. Ferri et al. 2003). The next assessment is planned to begin in 2024 when Study members turn 52.

In addition to the main Dunedin Study, a further five sub-studies have been undertaken, beginning with that led by Jay Belsky about cross-generational parenting practices in the mid-1990s (e.g. Belsky et al. 2001), through to recent studies about familial liability to neurodegenerative disease (e.g. Reuben et al. in press), and a short-window data capture related to vaccine hesitancy among Study members completed between May and August in 2021 (Moffitt et al. 2022). See Supplement Part 3 for more detail on the Dunedin sub-studies.

The Study has generated many insights into what constitutes poor and optimal human development across the lifecourse. Importantly, the findings have influenced research, policy, and practice nationally and internationally, and are likely to continue to do so in the future.

## Periodic re-invention to stay ahead of the curve

In the beginning, our research project was known as the Queen Mary Multidisciplinary Child Health and Development Study, named after the hospital in which Study members were born. The focus was on early developmental milestones and health indicators measured at age 3 and 5 years, with the overarching aim being to determine whether babies born in adverse circumstances (e.g. low birth weight, jaundice, poor APGAR scores) were developmentally compromised compared to babies who were not born in such circumstances. Following the age-five assessment, Dunedin Study publications revealed alarmingly high and hitherto unrecognised rates of a range of child health problems for example, glue ear. The founding director Phil Silva adroitly parleyed this ‘Child Health Shock’ (Evening Post Billboard, late 1970s) to persuade the then NZ Medical Research Council to extend funding for another six years. This enabled three further assessments at ages seven, nine and 11 years. According to Graeme Fraser (New Zealand’s first Sociology Professor, and a member of the original Medical Research Council assessment panel in 1978), the panel came to the view that research conducted ‘in a city like Dunedin with its medical and dental schools and social science experts from many disciplines had enormous potential’ (personal communication, 28th February, 2022).

The age-13 assessment heralded the second ‘version’ of the study. Several factors signalled this change. First, it evolved from being a child development study to a study about child and adolescent development. Our name changed to the Dunedin Multidisciplinary Health and Development Study (‘Dunedin Study’) to reflect this. Second, the founding director capitalised on growing international interest by recruiting Terrie Moffitt, a young post-doc from Sarnoff Mednick’s laboratory at the University of Southern California, to conduct research on antisocial behaviour. At the time, local experts opined that this would be a fruitless exercise because the cohort's rates of antisocial behaviour would be too low. This assumption turned out to be quite incorrect, and Moffitt’s developmental taxonomy of antisocial behaviour (Moffitt 1993), developed in the Dunedin Study, is now the most cited and influential in the history of criminology. Third, a precipitous drop in sample retention at age 13 (82%) resulted in the formulation of strategies to drive retention back up over 90% in future assessments.

After data from the age-18 assessment had been written up (circa 1990) many researchers who had been involved in the Study from the early years moved on to other positions, but some remained to help re-position the Study for the third time as one about ‘emerging adulthood’ – the period between ages 19 and 29 years (see Arnett, 2000 for a description of this developmental epoch). In the early 1990s, most developmental research tended to concentrate on periods of greatest developmental change, that is, the beginning and end of the lifecourse, with comparatively less attention paid to the intervening years. This lacuna in understanding development between post-secondary school and retirement offered an exciting opportunity to contribute new knowledge. Pivoting in this direction entailed planning for longer periods between assessments, moving from every two years during childhood to every five to seven years in adulthood. This longer periodicity helped ensure sufficient developmental change had occurred to justify inviting Study members back to the Dunedin Research Unit for a busy day of assessments. Since age 21, and coinciding with increased geographic mobility among Study members, we have endeavoured to remove as many barriers to participation as possible. This includes costing in economy plane flights for Study members from around New Zealand and from overseas, back to their town of birth, to undergo complex physiological assessments under controlled conditions (e.g. Milne et al. 2001).

This strategy – a combination of direct, comprehensive, clinical-quality measurement, normally only possible in experimental laboratory settings, but nested within an epidemiological sampling frame – is one of the early design features of the Dunedin Study that explains its longevity and relevance.

## The fourth re-invention as a ‘geroscience’ study

Early brainstorming about the shape of the phase 38 assessment (circa 2013–2014) saw a decision to recast the study as one about the aging process – but long before people had become old. Although emerging developmental science supported this vision, we found that it confused many reviewers. They questioned how we could study aging when our Study members were not yet ‘old’. This scepticism resulted in our 2015 Health Research Council (HRC) programme grant being triaged from the round, without interview, placing the future of the Study at risk. Since that vexatious time the Study has generated approximately 40 papers on various aspects of the aging process, placing it at the forefront of the rapidly evolving geroscience field.

Contrary to the expectations of those sceptical peer-reviewers, it became obvious to us that some Study members were becoming biologically older than their peers as they pass through midlife, while others were remaining biologically younger. Increasingly, midlife is important to prevention-minded clinicians and health-policy makers because it offers the opportunity to intervene to delay the late-life diseases that shrink the lifespan and healthspan of older adults. Simply put, prevention of diseases should work better than trying to reverse organ damage after diseases are established. However, there was a research gap, because most studies of aging enrol older-adult participants who are well past midlife, and most studies of younger adults do not measure aging. This gap meant that there was surprisingly little knowledge about aging during midlife (e.g. Moffitt et al. 2017). The Dunedin Study geroscience research programme is uniquely filling this gap (e.g. Belsky et al. 2015; Elliott et al. 2021). The geroscience agenda posits the exciting possibility that a therapy that can slow whole-body aging will also simultaneously delay the onset and slow the progression of all age-related cardiovascular, sensory, immune, neurodegenerative, and musculoskeletal diseases. We set out to develop a novel tool to measure a person’s pace of whole-body biological aging, and the result of our efforts is named DunedinPACE (Belsky et al. 2022). To develop it, we tracked biomarkers measuring the physiological functions of the Dunedin Study members for two decades into midlife. Our goal was to measure, in people the same chronological age, variation in biological aging that is defined according to geroscience theory as gradual, progressive, decline that simultaneously affects multiple organ systems. We tracked declines in the cardiovascular, metabolic, pulmonary, renal, dental, hepatic, and immune functions of Study members by repeatedly assessing 19 biomarkers at ages 26, 32, 38 and 45 years. Modelling this data yielded a pace-of-aging metric that quantified how slowly or rapidly each Study member had been aging (Belsky et al. 2015; Elliott et al. 2021). In the next stage of the work, we applied machine-learning to Study members’ whole-genome DNA methylation data, training an algorithm on that pace-of-aging metric. This work produced a score we called DunedinPACE, for Pace of Aging Computed in the Epigenome (Belsky et al. 2022). We undertook validation checks in other cohorts in the US and the UK, and these checks confirmed that people who have faster DunedinPACE subsequently experience advanced facial age appearance, declines in cognitive and physical functioning, more chronic diseases, and earlier mortality (Belsky, Caspi, Arseneault, et al. 2020; Belsky et al. 2022). DunedinPACE can be estimated from just a single blood sample, and this technical advance means that it can now be exported to any research study anywhere that has collected blood methylation data. The algorithm is available open-access on Github (https://github.com/danbelsky/DunedinPACE). We are presently developing a version of the tool using buccal methylation data, for use in settings where blood collection is not feasible. DunedinPACE is being incorporated into the open-access data sets of many large cohorts in several countries. As the field studies it, DunedinPACE is proving valid in men, women, older adults and adolescents, and in varied race/ethnic groups and nationalities. Intervention studies that aim to slow aging and extend healthspan are beginning to use DunedinPACE to test whether an intervention has indeed slowed a person’s whole-body pace of aging. Basic aging science, too, is using DunedinPACE in etiological research to identify new anti-aging targets, and in health-disparities studies of disadvantaged social groups who age at different rates. By delivering a reliable, valid, open-access tool to measure how rapidly a person has been aging the Dunedin Study has made a major contribution to the geroscience agenda of slowing aging to extend our years of disease-free life.

## Some drivers of success – guiding principles

### Study member needs are paramount – the golden rule

We aim to treat our Study members as we ourselves would like to be treated – with respect, care, honesty and kindness, and without judgment. The instantiation of this ideal relies upon us trying to place ourselves in their shoes, and walk for a kilometre or 10, to better understand what their needs might be at any particular point in time. For example, when we were planning our age-32 assessment we knew that the median age of first birth in Aoteraoa/New Zealand was 31 years. Possibly influenced by the Director just becoming a father himself, we decided to convert the staff tea-room into an inviting creche, with floor to ceiling murals, including a beautifully rendered painting of Nemo. We hired professional Early Childhood teachers for one day per week, and mothers (or fathers) who wished to bring their baby with them to the assessment day were booked on creche day. This allowed parents to visit their child at morning tea, lunchtime or afternoon tea, having confidence that their child was well looked after, and close by. The latter recognises the anxiety almost all new parents feel when parted from their babies.

A second example followed the Christchurch earthquake that killed 185 people. At the time we had 102 Study members living in Christchurch. The Director sent a handwritten card to all those living in Christchurch, expressing care and concern. This small gesture meant a lot to the recipients, with a number still expressing heartfelt appreciation many years later ‘for the thought’.

Cumulatively such actions help strengthen the bond and trust between study participants and the researchers, without which the sustainability of lifecourse research is more challenging. The emphasis on trust may seem obvious, and we assume that many other studies also place high value on trust. It may be the lengths we go to maintain and enhance trust that is key here.

### Excellence – traditional and non-standard yardsticks for impact

We strive to produce the best quality work and disseminate via the best quality journals and publishing houses, cognisant of our aim to reach target audience(s) who determine healthcare policy. Methodological rigour is the cornerstone of this mahi (work). This involves time and resources to ensure high quality data collection and subsequent data analysis. Some standard attributes of best practice we prioritise are multi-source (e.g. self, parent, teacher, official records), multi-method (e.g. self-report, informant-report, direct observation, physiological, bioassay) data collection, recognising that most methods come with at least some source of error. The ability to ‘triangulate’ across time and source strengthens our ability to create robust dependent, independent and covariate variables. We also engage directly in methodological research to, for example, determine the impact of different design approaches (e.g. prospective versus retrospective, Reuben et al. 2016); conduct comparisons of popular commercial tests for biomarkers (e.g. Sugden et al. 2020); and to establish test-retest reliability of measures newly introduced to the Study, like fMRI scans. In the case of the latter, the Study raised serious questions for the field due to low concordance between repeat fMRI scans acquired over a short time period (Elliott et al. 2020). We have also contributed to the debate around research studies making the data they collect readily available to the public, noting that researchers, funders, and journals all have some form of ‘conflict-of-interest’ in this debate, yet those most critical to the research – the Study members – largely remain voiceless (Reeves et al. 2021, 2022).

The Dunedin Study has been productive. It has made significant contributions in many areas related to human development. These include: (i) mental health and well-being with more than 150 papers on, for example, prevalence of pre-adolescent psychiatric disorders (Anderson et al. 1987); early life risk factors (e.g. Poulton et al., 2000; Cannon et al. 2002; Koenen et al. 2007); natural history showing that most adult disorders have a juvenile onset (Kim-Cohen et al. 2003); developmental heterogeneity and implications for the timing and nature of interventions (Jaffee et al. 2002); comorbidities with poor physical wellbeing (Odgers et al. 2007); the role of nature-nurture interplay in risk and resilience for mental wellbeing (Caspi et al. 2002, 2003); that most of us will meet criteria for at least one psychiatric disorder at some point in our lives (Schaefer et al. 2017); and that the ‘true’ structure of psychopathology differs from that enshrined in current taxonomies (Caspi et al. 2014, 2020). Recently some of these contributions were acknowledged as being among the most important in the last 100 years (Science News, 2022).

The oral health programme has produced over 80 papers, with unprecedented information on the natural history of oral diseases from childhood to midlife, including the unexpected finding that the average caries increment with age is constant through life (Broadbent et al. 2013); tooth loss to caries begins in late adolescence (Thomson et al. 2000); early childhood caries is a risk factor for ongoing adult dentition caries (Broadbent et al. 2008); identification of different trajectories of dental anxiety (Thomson et al. 2009); cannabis use is an independent (from tobacco) risk factor for periodontal disease (Thomson et al. 2008), and the characterisation of the widening of social inequalities as we pass through adulthood (Thomson et al. 2004).

The respiratory programme of research has also been particularly prolific with close to 90 papers, leading to many insights related to the developmental course of asthma (e.g. Sears et al. 2003), early life risk factors (e.g. Sears et al. 2002): antecedents of allergy and atopy (e.g. Mandhane et al. 2009); the impacts of tobacco and cannabis on COPD symptoms and lung function (e.g. Hancox et al. 2022); and the genetics of asthma (e.g. Belsky, Sears et al. 2013).

In the social and behavioural domains, we have made a number of important contributions (e.g. Poulton et al. 2002; Moffitt et al. 2011; Belsky, Caspi, Moffitt et al. 2020), beginning with the seminal publication by Terrie Moffitt in 1993 describing a developmental taxonomy of antisocial behaviour. This has become the most influential theory in the field of criminology, garnering over 13,000 citations to date. It, along with the many papers that have flowed from the study, testing the robustness and value of the theory (e.g. Odgers et al. 2008; Moffitt 2018) have been incredibly influential in the judiciary (e.g. sentencing) and policing more generally, in both New Zealand (personal communication Judge Andrew Becroft, 2020, former head of the Youth Court and a recently retired Children’s Commissioner), as well as overseas. For example, it contributed to a successful Amicus Brief that was heard by the U.S. Supreme Court aimed at changing the law regarding execution of those who offended under the age of 18 (Moffitt 2018), which resulted in 73 people on death row having their sentence cancelled immediately.

Other notable themes of research within the Dunedin Study have included cardiovascular risk (e.g. Caspi et al. 2006; Theodore et al. 2015), sexual and reproductive health (e.g. Dickson et al. 1998; van Roode et al. 2015), vision (Simpson, Kirkland, & Silva, 1984; Singh et al. 2022; Wilson et al. 2021); hearing (Chalmers et al. 1989; Welch and Dawes 2007); and personal relationships (Ehrensaft et al. 2004; Bourassa et al. 2020).

There are also many ‘niche’ streams within the Dunedin Study, for example, work on: (i) sleep (e.g. Goldman-Mellor et al. 2015), (ii) cannabis use and its consequences (e.g. Poulton et al. 2020; Meier et al. 2022); irritable bowel syndrome (e.g. Talley et al. 2001, 2004); health risk behaviours like sedentary behaviour (Hancox et al. 2004), physical activity (Richards et al. 2009), tobacco and alcohol use (e.g. Meier et al. 2012; Belsky, Moffitt et al. 2013); the impact of lead exposure on development (e.g. Reuben et al. 2017, 2019, 2020), and more recently, the relation between brain structure and pace of aging (Elliott et al. 2021), psychiatric outcomes (Romer et al. 2021), and neurocognition (d’Arbeloff et al. 2021).

Increasingly, the highest impact papers from lifecourse research combine multi-domain information, gathered over multiple developmental epochs, leveraging multi-method and multi-source data. Less appreciated are the specialist skills required to work in this way. Our experience suggests that only highly experienced lifecourse researchers are able to lead such complex undertakings, whereas new recruits to lifecourse research are best to ‘cut their teeth’ on discipline-specific papers. The Dunedin Study recruits the best of early-career researchers, from at home and abroad. And their training can take time. Given that the best contemporary lifecourse research is holistic, capturing many levels, and crossing multiple disciplinary boundaries, the aim should be to systematically train the next cadre of lifecourse researchers to be ‘expert generalists’. Class 101 should ideally emphasise methodological rigour, as above.

We have also sought to achieve excellence through less ‘traditional’ pathways. Here we refer to efforts to disseminate our research as widely as possible. We think this should be obligatory but most researchers understandably focus on those activities that are incentivised within academia, that is, academic conference presentations and publishing in peer-reviewed journals. Examples of our recent efforts to disseminate in novel ways include the multi award-winning four-part documentary TV series ‘Why am I’ produced by Mark McNeill, founder of Razor Films. This popular series has been shown in countries that include over half the world’s population. However, it was only possible due to Mark McNeill’s respect for the both the science (he is a former neuroscientist) and our commitment to protecting Study members’ anonymity (he used professional actors in the series to bring the narrative to life). Several attempts to produce a TV series in the past had fallen over, for a variety of reasons, so this was not a straightforward undertaking. Suffice to say, it also took a number of years to bring this project to fruition, requiring considerable time commitment by both the documentary-makers and the researchers featured.

A second example of non-traditional dissemination involves creation of a multi-decade museum exhibition on the Dunedin Study entitled ‘A Slice of Life’, which ran at Toitū Otago Early Settlers Museum between March 2016 and May 2017 and was enjoyed by over 200,000 visitors. An updated and expanded travelling version of the exhibition was then developed and toured to Canterbury Museum between November 2019 and June 2020, before travelling to the Museum of Transport and Technology in Auckland, with its particular focus on educational visits by school groups, from June 2020 to October 2020. Following this the exhibition moved south to Nelson Provincial Museum between October 2020 and April 2021 before returning to Dunedin to a pop-up museum from June 2021 to August 2021 as part of the New Zealand International Science Festival, made possible by a gift from a major local business owner. The tour was subject to multiple closures because of various COVID related interventions but still amassed over 300,000 visitations giving a total in excess of 500,000 visits for the ‘Slice of Life’ experience. The quality of both exhibitions was exceptional and involved sterling work by the professionals at Toitū Otago Early Settlers Museum and one dedicated staff member from our Research Unit, Sean Hogan.

This last point illustrates more generally the importance of support (non-academic) staff, particularly those who have been involved in the Study for a long time. They hold invaluable knowledge about many procedural aspects of how the Study operates, as well as institutional practices, and they play a crucial role in the smooth running of our periodic assessments. They are often well-known by the Study members and contribute to the maintenance of good relationships.

### Internationalisation – the benefits of being outward-looking

For more than two-thirds of the life of the Dunedin Study we have received support from overseas public good funding agencies (from the US and UK). This typically has come via our long-term investigators based overseas Terrie Moffitt and Avshalom Caspi, and more recently Ahmad Hariri. The major exception has been the oral health programme of research led by Dunedin-based investigator Murray Thomson who has secured multiple overseas grants. Both represent major achievements, recognising the high standing of the investigators, but also the unique qualities of the Dunedin Study more generally. That is, all public funding agencies deal with constraints around disbursable funds, and many are loathe to send money off-shore for fear of local backlash. A special case has to be made for international transfer of funds demonstrating that the proposed work cannot be done locally and that the Dunedin Study provides the best opportunity to ask and answer questions of importance to the international funder. A corollary of this is that Dunedin Study findings are regarded as generalisable to these populations thereby enhancing uptake and application of Study findings more globally. Empirical work has shown that this is indeed the case (Caspi et al. 1994).

The leveraging of both money as well as intellectual property from overseas means that the Dunedin Study has been able to conduct in-depth and costly assessments which would not have been possible were we solely reliant on local funding mechanisms. More than that, we have benefitted enormously from the knowledge and international perspectives brought by our long-term colleagues based overseas. For example, Terrie Moffitt’s roles include chairing the Board on Behavioral, Cognitive, and Sensory Sciences at the National Academy of Sciences, and the National Advisory Council on Aging in the United States, and membership of the board of the Nuffield Foundation in the UK. Such input allows the Dunedin team to be privy to international trends at the highest levels. This has undoubtedly helped us remain at the cutting edge internationally. Of passing interest and recalling the recent emphasis on economic returns from research, we estimate that approximately $20M in overseas funding has been injected into the Dunedin economy over the years. This compares favourably with the research projects funded specifically to produce economic benefits.

Finally, to ensure maximum return from the data and the inclusion of new research colleagues (both local and international) we assign Associated Investigator (AI) status to applicants who submit a concept paper outlining their proposed research (see https://dunedinstudy.otago.ac.nz/for-investigators/policy-statement-code-of-practice for more detail). To receive approval four criteria must be met: (i) the project must have some public health benefits; (ii) the project is not already contracted for via existing grants; (iii) there are sufficiently robust data to provide a meaningful test of the hypothesis (i.e. weak proxies will not suffice); and (iv) there is a Principal Investigator available to oversee and support the AI’s work.

### Striking the right balance – between low-moderate risk (90%) versus high-risk (10%) research

Whenever an assessment phase is planned we identify assessment protocols that are known to provide strong tests of our hypotheses. When formulating our hypotheses, we seek to push the boundaries on what is known, in the hope of generating new and useful information. The majority of this science is not conservative, but it is comparatively ‘safe’ in terms of likely returns. In contrast, true discovery often entails taking risks, so we try to judiciously select a small number of avant-garde assessments at each assessment wave. Some have been very generative, for example, measures of retinal function at age 38 (e.g. Meier et al. 2013; Shalev et al. 2013) and 45 (e.g. Barrett-Young et al. 2022); along with the potential of 3-D oral health scans (Olliver et al., 2022) whereas others have returned less, for example, measures of cardiovascular reactivity to stress (Bourassa et al. 2021) and endothelial function (Williams et al. 2017).

When considering the right balance between safe and high-risk research, the wellbeing of Study members remains uppermost in our thinking. Constant monitoring of the overall ‘burden’ of a busy assessment day (8.5 h) is necessary because we try to avoid leaving our Study members wiped out, having responded to hundreds of questions and undertaken a variety of physiological assessments. In this regard, the Director (as well as senior team members) always put themselves through the entire assessment day before the phase launch, after adopting a persona with multiple challenges, to gauge first-hand the level of demand and its tolerability.

### Horizon scanning

When planning an assessment phase, we strive to anticipate future opportunities and needs. We push ourselves past the first and most obvious horizon, whilst all the time looking back at the data already held to maximise its value. Simultaneously looking forward and back seems to us to be an important element in successful lifecourse research. The art of effective horizon scanning relies upon broad experience, both within the research world and beyond such that community, policy, practice and political perspectives should inform the process. In concrete terms, this involves getting out of a ‘protected’ academic mindset into the real world. Finally, having a diversity of perspectives around the table when horizon scanning can add great value.

## Impact

Despite numerous attempts to measure the impact of research it is surprisingly hard to operationalise. Established metrics (e.g. citation indices) are useful for judging impact in the academic world but tells us little about whether the research has informed better policy formation or practice, and/or whether this has improved the wellbeing of the citizenry. Opportunities to serve as conduits for research beyond academic networks do exist, for example, the Director worked part-time as a Chief Science Advisor to several branches of the New Zealand government between 2014 and 2021. Following up on published work (i.e. it’s the beginning not the end of the process) to ensure correct understanding and uptake is likely to become even more important in the future.

Lifecourse research is ideally positioned to contribute in this space, but there are some prerequisites. First, academic hubris must be left at the door, its presence guarantees failure. Second, policy-making is a highly skilled profession, requiring skills foreign to most researchers. It will take time to learn, which will be made easier if there is respect for policy-making colleagues and the unique pressures they face. Third, developing high trust is essential. Never hype or misrepresent what the research says, and always point out the confidence intervals around your advice. Fourth, and a corollary of the above, always stick to the data no matter how unpopular, but do so respectfully. Fifth, be mindful of the context, so for example, if you are in a political environment know that politics will always trump evidence when the heat is on. Don’t get despondent when things don’t go your way, be resilient instead. Sixth, timing is everything. All of the above seem obvious, so the above borders on the banal, however moving research results into policy and practice is far harder to achieve in reality, hence the importance of persistence.

## Lessons learned, the hard way

Despite our best efforts we have not always got it right. Here we present three broad categories of potential error based on our experience, in the hope of informing others so they might avoid these pitfalls. Here we discuss them separately, but if the categories compound, life can become very difficult.

### Don’t believe the hype – even from experts

As our research interests and horizons have broadened over time, so has our desire to undertake more novel data collection, but often in areas where we lack sufficient knowledge. Our approach to this situation has been, on the face of it, sensible. We reach out to acknowledged experts in the field for advice. To ensure the best input we typically invite the experts to become collaborators on the project, which serves to elicit maximum technological input, guidance and support. However, as the old adage goes ‘it’s hard to know what you don’t know’. We have encountered this problem several times over the years and here we describe how easy it is to fall into this trap. Assume our expert is someone who built their career on measuring cardiovascular reactivity, initially by using animal models then moving to testing in analogue samples. Proof of principle for the use of the approach was strong in the literature. The paradigm was implemented faithfully. The results were significant but in the opposite direction to that expected, and no amount of checking and reflection could shed light on why this was. This confounded us at the time, and we have only recently had a glimmer of insight into what might explain this result (Bourassa et al. 2021). In the simplest terms our general population sample has different characteristics from analogue (tertiary student) samples which were magnified using this paradigm. In hindsight it makes sense, but this was not apparent to any of the players (including the expert) at the time.

### Biting off more than you can chew

In more recent assessments we have added several innovative measures, for example, gaitrite (Rasmussen et al. 2019), Optical Coherence Tomography (OCT, Barrett-Young et al., 2022), urine collection (e.g. Guiney et al. submitted), DXA scanning (e.g. Meredith-Jones et al. 2021), and 3-D dental imaging (Olliver et al. 2022) to increase the breadth and/or depth of data gathered. This expansion has also seen the re-introduction (from childhood) of vision and hearing assessments, plus the seeding of new programmes of research on musculoskeletal health, kidney function and oxidative stress. The inclusion for the first time at phase 45 of MRI scanning required an additional one-third of a day to complete.

Unsurprisingly this has added significant complexity to the overall operation and on reflection, it seems we did not make sufficient allowance for this in our timeline and schedule of deliverables in the lead up to the launch of the most recent assessment phase. This resulted in steep learning curves for our intake of research interviewers/assessors (approximately *n* = 30 new, part-time staff were taken on at age 45). Not all gremlins had been ironed out ahead of time for some of the new, more complex physiological assessments. The time allocated to piloting and dress rehearsal phase had remained similar over the years, despite added complexity. While it is true that we managed to pull off a very successful age 45 assessment it did involve some avoidable stress for staff and management, especially early on. Thankfully, the fix is straightforward here: better, more advanced planning, setting of milestones, and enforcement of deadlines. For example, in the lead up to phase 52 investigators who have not fully piloted their protocols six months prior to launch (i.e. feasibility, acceptability, timing, quality assurance process, test-retest reliability, and data transfer procedures) will become ineligible for Phase 52. Naturally, this will increase costs so this also needs to be factored in.

### Measurement continuity

For a variety of reasons, measurement methods change from time to time. The reasons for this are manifold, for example, new and better assessment equipment becomes available, suppliers of bioassays develop new products and medical companies providing high-cost equipment (e.g. MRI scanners) choose to upgrade machines. We first ran into this challenge when reviewing our age-38 bioassay plans due to notification from manufacturers that some assays were going to change during the assessment period. We sought external advice from those working in the biomarker research field about standard ways of dealing with such changes. It proved difficult to divine a consensus view on this, leaving the impression that this is an issue that is often swept under the rug, the opposite of what is required. We invested in developing novel methods of calibrating to harmonise old with new assays. Maintaining a heightened sensitivity to the importance of these types of methodological issues is critical going forward and strategies are required for transitioning from past to new approaches.

## Future studies – reframing extant data

In addition to advancing the Dunedin Study’s major programme of research focused on geroscience, the future also provides the opportunity to reframe the extant data to investigate new and topical issues of the day and to use findings from the Dunedin Study to inform interventions aimed at improving health and social outcomes.

## Social cohesion

In lay terms social cohesion refers to members of a society feeling and acting in solidarity, or ‘sticking together’ to achieve mutually beneficial goals, and is essential for the effective functioning and wellbeing of society (Chan et al. 2006; Fonseca et al. 2019). More formally, scholars define social cohesion as a product of the horizontal (among fellow citizens) and vertical (with government or other institutions) relationships between members of a society, which can be assessed through people’s attitudes and behaviours that reflect those relationships. The concepts of sense of belonging, trust in fellow citizens, willingness to co-operate and help others, social participation, inclusion and recognition of diverse members, trust in public figures, trust and confidence in institutions, and political participation all reflect social cohesion (Spoonley et al. 2005; Chan et al. 2006).

Given the importance of social cohesion for a well-functioning and adaptable society, governance organisations around the world view it as an important policy goal, and seek to foster societal conditions that help to maintain and promote it (OECD 2011a; New Zealand Treasury 2021). To properly inform such work, it is essential to empirically test the factors that promote or inhibit social cohesion (Chan et al. 2006; Gluckman et al. 2021). To date, such work has focused predominantly on examining concurrent associations between socio-political conditions (e.g. environmental conditions, inequality, crime, economic insecurity, immigration) and adults’ endorsement of socially cohesive attitudes and/or engagement in socially cohesive behaviours at a particular point in time (e.g. Laurence 2011; OECD 2011b; Vergolini 2011). However, proximal socio-political conditions may not fully account for variations in social cohesion. As we have learned from the Dunedin Study, people’s experiences and attributes in childhood are important predictors of health and psychosocial outcomes in adulthood, and those same factors are likely to also influence the perceptions, values, and behaviours that comprise socially cohesive behaviour. Indeed, recent Dunedin Study work has identified several developmental antecedents of a specific socially cohesive behaviour in adulthood: COVID-19 vaccine acceptance (Moffitt et al. 2022).

We plan to test this hypothesis further in the Dunedin Study cohort by examining the developmental origins of social cohesion, as indicated by people’s socially cohesive attitudes and behaviours. We will first explore the longitudinal links between childhood experiences and indicators of the horizontal aspect of social cohesion measured in adulthood, including volunteering in the community, charitable donations, social participation, and willingness to help people in their community. In future phases, we plan to broaden our social cohesion measures to include a wider set of horizontal indicators as well as indicators of the vertical aspect, such as trust in government and other institutions. By examining the developmental origins of both the horizontal and vertical aspects of socially cohesive attitudes and behaviour in the same population, we aim to make a unique contribution to the literature on social cohesion (Fonseca et al. 2019) and ultimately to provide insight into policy settings that can be implemented early in the life course to help foster the development of healthy, reliable, resilient, communities that are best able to cope with rapid and unpredictable societal change.

## From knowledge generation to knowledge application

Using Dunedin Study findings to inform the focus and design of multiple randomised controlled trials (RCTs) has been an emerging trend over the last 10 years. At the time of writing a number of intervention projects are informally connected to Dunedin Study researchers. In the future we plan to formalise these linkages in order to cover more of the research pipeline. Horizon scanning suggests this will become more attractive to funders in the future. Intervention partnership projects include:

1. Māori immersion preschool RCT supported by Te Pou Tiringa Incorporated. Funded by the Health Research Council and Ministry of Business, Innovation and Employment ($2.7M), to begin July 2022 through to 2025. The percentage of Māori within the Dunedin Study (approximately 7.5%) is lower than the national average. This means we have limited power to detect meaningful differences between Māori and non-Māori. However, there is ample opportunity for Study findings to be applied and tested by Māori in kaupapa Māori research and other contexts to ensure maximum benefits for Māori. This RCT is the instantiation of this approach (Edwards et al. In press).
2. Language and self-regulation RCT. Funded by philanthropy, 2021–2026. Partner is the Best Start early childhood education service. Measurement from 18 months to age 6 years, with repeated measurement every six months.
3. RCT of e-based intervention for mental health ‘Just a Thought’. $2 million already invested into development by a major non-governmental organisation.
4. Dunedin North Community Project He Ao Hou. Bringing together all social services covering one-quarter of the Dunedin population to optimise child development. Funded by the Otago Community Trust.

## Future challenges

### Sustainability

The perennial challenge for lifecourse research is financial sustainability. The travails encountered by the UK longitudinal studies testify to the universality of this challenge (e.g. Pearson 2016). Lifecourse studies are expensive, resource intensive, and time-hungry. They make funders nervous. They should be embarked upon with eyes-wide-open, and after confirming that no other simpler design is fit-for-purpose. The funding mechanisms available in New Zealand are time-limited (five-year cycles are the norm) and contestable. Philanthropy remains an option albeit one that is still maturing in New Zealand. Big business and/or commercial support does not fit with the ethos of science for public good and hence is not an option. The bottom line is that we must remain relevant and agile in a capricious funding environment. Ideally, established longitudinal studies will come to be seen as essential science infrastructure.

### Replication and reproducibility – seek out partner studies

The most straightforward way to address this big-picture issue is to team up with other lifecourse studies to address the same question in two or more cohorts, ideally in different locations around the world. This provides confidence regarding the robustness of the findings. In recent years, the Dunedin Study has made a concerted effort to work with others in pursuit of this model (Copeland et al. 2013; Belsky et al. 2018; Becker et al. 2021; Demange et al. 2021; Rocha et al. 2021). Linked to this is also our desire to be good academic citizens and contribute to large consortia while still protecting our contract with Study members to protect their confidentiality and ensure controlled access.

### Leadership – stability and fresh blood

As confirmed by many Study members over the years, the high trust in the Study is in part attributable to the stability of the leadership. Phil Silva founded the study in 1972 and retired from the directorship in 1999. Current director Richie Poulton has been in this role since 2000 and prior to that was deputy director from 1995. Associate director Terrie Moffitt joined the study when the Study members were aged 13 and first employed Poulton as research interviewer in 1985–1986. Their relationship has survived for almost four decades – an unusually long period of continuity. The transition from Poulton to the next director, whomever that may be, will ideally see an early’ish career appointment so that person has the potential to serve for a quarter-century, initially supported by senior players. This strategy will help maintain high levels of trust with Study members. The desirability of this continuity is often unrecognised. However, it is a fundamental feature of long-term success, and will help to ensure the Dunedin Study can keep moving with the times and stay at the cutting edge of health and development science.

## Supplementary Material

Supplemental Material

## References

[CIT0001] Anderson J, Williams SM, McGee R, Silva PA. 1987. DSM III disorders in preadolescent children: prevalence in a large sample from the general population. Arch Gen Psychiatry. 44:69–76. 2432848 10.1001/archpsyc.1987.01800130081010

[CIT0002] Arnett JJ. 2000. Emerging adulthood: a theory of development from the late teens through the twenties. Am Psychol. 55:469–480. 10842426

[CIT0003] Barrett-Young A, Cheyne K, Guiney H, Kokaua J, Steptoe B, Tham YC, Wilson GA, Wong TY, Poulton R. 2022. Associations between retinal nerve layer and ganglion cell layer in middle age and cognition from childhood to adulthood. JAMA Opthalmol. Published online February 10, 2022. doi:10.1001/jamaophthalmol.2021.6082. PMC8832305 35142821

[CIT0004] Becker J, Burik CAP, Goldman G, Wang N, Jayashankar H, Bennett M, Belsky DW, Karlsson Linner R, Ahlskog R, Kleinman A, et al. 2021. Resource profile and user guide of the polygenic index repository. Nat Hum Behav. 5:1744–1758. 34140656 10.1038/s41562-021-01119-3 PMC8678380

[CIT0005] Belsky DW, Caspi A, Arseneault L, Baccarelli A, Corcoran DL, Gao X, Hannon E, Harrington HL, Rasmussen LJ, Houts R, et al. 2020. Quantification of the pace of biological aging in humans through a blood test, the DunedinPoAm DNA methylation algorithm. eLife – Epidemiology and Global Health. 9:e54870. 10.7554/eLife.54870 PMC7282814 32367804

[CIT0006] Belsky DW, Caspi A, Corcoran DL, Sugden K, Poulton R, Arseneault L, Baccarelli A, Chamarti K, Gao X, Hannon E, et al. 2022. DunedinPACE: a DNA methylation biomarker of the pace of aging. eLife. 11:e73420:1–26. 10.7554/eLife.73420 PMC8853656 35029144

[CIT0007] Belsky DW, Caspi A, Houts R, Corcoran DL, Danese A, Harrington HL, Israel S, Levine M, Schaefer J, Sugden K, et al. 2015. Quantification of biological aging in young adults. PNAS. 112:E4104–E4110. 26150497 10.1073/pnas.1506264112 PMC4522793

[CIT0008] Belsky DW, Domingue BW, Wedow R, Arseneault L, Boardman J, Caspi A, Conley D, Fletcher JM, Freese J, Herd P, et al. 2018. Genetic analysis of social-class mobility in five longitudinal studies. PNAS. 115:E7275–E7284. 29987013 10.1073/pnas.1801238115 PMC6077729

[CIT0009] Belsky DW, Moffitt TE, Baker TB, Biddle AK, Evans JP, Harrington HL, Houts R, Meier MH, Sugden K, Williams BS, et al. 2013. Polygenic risk and the developmental progression to heavy, persistent smoking and nicotine dependence: evidence from a 4-decade longitudinal study. JAMA Psychiatry. 70:534–542. 23536134 10.1001/jamapsychiatry.2013.736 PMC3644004

[CIT0010] Belsky DW, Sears MR, Hancox RJ, Harrington HL, Houts R, Moffitt TE, Sugden K, Williams BS, Poulton R, Caspi A. 2013. Polygenic risk and the development and course of asthma: an analysis of data from a four-decade longitudinal study. Lancet Respir Med. 1:453–461. 24429243 10.1016/S2213-2600(13)70101-2 PMC3899706

[CIT0011] Belsky J, Caspi A, Moffitt TE, Poulton R. 2020. The origins of you: how childhood shapes later life. Cambridge (MA): Harvard University Press.

[CIT0012] Belsky J, Jaffee SR, Hsieh KH, Silva PA. 2001. Childrearing antecedents of intergenerational relations in young adulthood: a prospective study. Dev Psychol. 37:801–813. 11699754 10.1037//0012-1649.37.6.801

[CIT0013] Bourassa KJ, Caspi A, Harrington H, Houts R, Poulton R, Ramrakha S, Moffitt TE. 2020. Intimate partner violence and lower relationship quality are associated with faster biological aging. Psychol Aging. 35:1127–1139. 33211513 10.1037/pag0000581 PMC7712579

[CIT0014] Bourassa KJ, Moffitt TE, Harrington HL, Houts RM, Poulton R, Ramrakha S, Caspi A. 2021. Lower cardiovascular reactivity is associated with more childhood adversity and poorer midlife health: replicated findings from the Dunedin and MIDUS cohorts. Clin Psychol Sci. 59:1–18. 10.1177/2167702621993900 PMC8547549 34707918

[CIT0015] Broadbent JM, Foster-Page LA, Thomson WM, Poulton R. 2013. Permanent dentition caries through the first half of life. Br Dent J. 215:E12. 24113990 10.1038/sj.bdj.2013.991

[CIT0016] Broadbent JM, Thomson WM, Poulton R. 2008. Trajectory patterns of dental caries experience in the permanent dentition to the fourth decade of life. J Dent Res. 87:69–72. 18096897 10.1177/154405910808700112 PMC2254448

[CIT0017] Buckfield PM. 1978. Perinatal events in the Dunedin city population 1967–73. N Z Med J. 88:244–246. 281622

[CIT0018] Cannon M, Caspi A, Moffitt TE, Harrington HL, Taylor A, Murray RM, Poulton R. 2002. Evidence for early-childhood, pan-developmental impairment specific to schizophreniform disorder: results from a longitudinal birth cohort. Arch Gen Psychiatry. 59:449–456. 11982449 10.1001/archpsyc.59.5.449

[CIT0019] Caspi A, Harrington HL, Moffitt TE, Milne BJ, Poulton R. 2006. Socially isolated children 20 years later: risk of cardiovascular disease. Arch Pediatr Adolesc Med. 160:805–811. 16894079 10.1001/archpedi.160.8.805

[CIT0020] Caspi A, Houts R, Belsky DW, Goldman-Mellor S, Harrington HL, Israel S, Meier MH, Ramrakha S, Shalev I, Poulton R, et al. 2014. The p factor: one general psychopathology factor in the structure of psychiatric disorders? Clin Psychol Sci. 2:119–137. 25360393 10.1177/2167702613497473 PMC4209412

[CIT0021] Caspi A, Houts RM, Ambler A, Danese A, Elliott ML, Hariri A, Harrington H, Hogan S, Poulton R, Ramrakha S, et al. 2020. Longitudinal assessment of mental health disorders and comorbidities across 4 decades among participants in the Dunedin birth cohort study. JAMA Network Open. 3:e203221–e203221. 32315069 10.1001/jamanetworkopen.2020.3221 PMC7175086

[CIT0022] Caspi A, McClay J, Moffitt TE, Mill JS, Martin J, Craig I, Taylor A, Poulton R. 2002. Role of genotype in the cycle of violence in maltreated children. Science. 297:851–854. 12161658 10.1126/science.1072290

[CIT0023] Caspi A, Moffitt TE, Silva PA, Stouthamer-Loeber M, Krueger RF, Schmutte PS. 1994. Are some people crime-prone? Replications of the personality-crime relationship across countries, genders, races and methods. Criminology. 32:163–196.

[CIT0024] Caspi A, Sugden K, Moffitt TE, Taylor A, Craig I, Harrington HL, McClay J, Mill JS, Martin J, Braithwaite A, et al. 2003. Influence of life stress on depression: moderation by a polymorphism in the 5-HTT gene. Science. 301:386–389. 12869766 10.1126/science.1083968

[CIT0025] Chalmers D, Stewart I, Silva P, Mulvena A. 1989. Otitis media with effusion in children – the Dunedin study. Oxford: Mac Keith Press.

[CIT0026] Chan J, To H-P, Chan E. 2006. Reconsidering social cohesion: developing a definition and analytical framework for empirical research. Social Indicators Research. 75(2):273–302.

[CIT0027] Copeland WE, Adair CE, Smetanin P, Stiff D, Briante C, Colman I, Fergusson DM, Horwood LJ, Poulton R, Costello J, et al. 2013. Diagnostic transitions from childhood to adolescence to early adulthood. J Child Psychol Psychiatry. 54:791–799. 23451804 10.1111/jcpp.12062 PMC3674149

[CIT0028] d'Arbeloff T, Cooke M, Knodt AR, Sison M, Melzer TR, Ireland D, Poulton R, Ramrakha S, Moffitt TE, Caspi A, et al. 2021. Is cardiovascular fitness associated with structural brain integrity in midlife? Evidence from a population-representative birth cohort study. Aging. 13:1–11. 33082296 10.18632/aging.104112 PMC7655208

[CIT0029] Demange P, Malanchini M, Mallard T, Biroli P, Cox S, Grotzinger A, Tucker-Drob E, Abdellaoui A, Arseneault L, van Bergen E, et al. 2021. Investigating the genetic architecture of non-cognitive skills using GWAS-by-subtraction. Nat Genet. 53:35–44. 33414549 10.1038/s41588-020-00754-2 PMC7116735

[CIT0030] Dickson N, Paul C, Herbison GP, Silva PA. 1998. First sexual intercourse: age, coercion, and later regrets reported by a birth cohort. BMJ. 316:29–33. 9451263 10.1136/bmj.316.7124.29 PMC2665316

[CIT0031] Edwards W, Hond R, Ratima R, Tamati A, Treharnee GJ, Hond-Flavell E, Theodore R, Carrington SD, Poulton R. In Press. Tawhiti nui, tawhiti roa: Tawhiti tūāuriuri, tawhiti tūāhekeheke: A Māori lifecourse approach and its application to longitudinal research.

[CIT0032] Ehrensaft MK, Moffitt TE, Caspi A. 2004. Clinically abusive relationships in an unselected birth cohort: men's and women's participation and developmental antecedents. J Abnorm Psychol. 113:258–270. 15122946 10.1037/0021-843X.113.2.258

[CIT0033] Elliott ML, Belsky DW, Knodt AR, Ireland D, Melzer TR, Poulton R, Ramrakha S, Caspi A, Moffitt TE, Hariri A. 2021. Brain-age in midlife is associated with accelerated biological aging and cognitive decline in a longitudinal birth-cohort. Mol Psychiatry. 26:3829–3838. 31822815 10.1038/s41380-019-0626-7 PMC7282987

[CIT0034] Elliott ML, Caspi A, Houts RM, Ambler A, Broadbent JM, Hancox RJ, Harrington H, Hogan S, Keenan R, Knodt A, et al. 2021. Disparities in the pace of biological aging among midlife adults of the same chronological age have implications for future frailty risk and policy. Nat Aging. 1:295–308. 33796868 10.1038/s43587-021-00044-4 PMC8009092

[CIT0035] Elliott ML, Knodt AR, Ireland D, Morris ML, Poulton R, Ramrakha S, Sison ML, Moffitt TE, Caspi A, Hariri AR. 2020. What is the test-retest reliability of common task-fMRI measures? New empirical evidence and a meta-analysis. Psychol Sci. 31:792–806. 32489141 10.1177/0956797620916786 PMC7370246

[CIT0036] Ferri S, Bynner J, Wadsworth M. 2003. Changing Britain, changing lives: three generations at the turn of the century. London: Institute of Education, University of London.

[CIT0037] Fonseca X, Lukosch S, Brazier F. 2019. Social cohesion revisited: A new definition and how to characterize it. Innovation: The European Journal of Social Science Research. 32(2):231–253.

[CIT0038] Gluckman P, Bardsley A, Spoonley P, Royal C, Simon-Kumar N, Chen A. 2021. Sustaining aotearoa New Zealand as a socially cohesive society. Auckland: Koi tū: The Centre for Informed Futures.

[CIT0039] Goldman-Mellor S, Caspi A, Gregory AM, Poulton R, Moffitt TE. 2015. Is insomnia associated with deficits in neuropsychological functioning? Evidence from a population-based study. Sleep. 38:623–631. 25348123 10.5665/sleep.4584 PMC4355902

[CIT0040] Guiney H, Walker R, Broadbent J, Caspi A, Goodin E, Kokaua J, Moffitt TE, Robertson S, Theodore R, Poulton R, et al. Kidney-function trajectories from young adulthood to midlife: identifying risk strata and opportunities for intervention. Submitted to Kidney International Reports. 10.1016/j.ekir.2022.10.005 PMC9831942 36644353

[CIT0041] Hancox RJ, Milne BJ, Poulton R. 2004. Association between child and adolescent television viewing and adult health: a longitudinal birth cohort study. Lancet. 364:257–262. 15262103 10.1016/S0140-6736(04)16675-0

[CIT0042] Hancox RJ, Xian Zhang ARG, Poulton R, Moffitt TE, Caspi A, Sears MR. 2022. Differential effects of cannabis and tobacco on lung function in mid-adult life. Am J Respir Crit Care. doi:10.1164/rccm.202109-2058OC. PMC9872808 35073503

[CIT0043] Jaffee SR, Moffitt TE, Caspi A, Fombonne E, Poulton R, Martin J. 2002. Differences in early childhood risk factors for juvenile-onset and adult-onset depression. Arch Gen Psychiatry. 59:215–222. 11879158 10.1001/archpsyc.59.3.215

[CIT0044] Kim-Cohen J, Caspi A, Moffitt TE, Harrington HL, Milne BJ, Poulton R. 2003. Prior juvenile diagnoses in adults with mental disorder: developmental follow-back of a prospective-longitudinal cohort. Arch Gen Psychiatry. 60:709–719. 12860775 10.1001/archpsyc.60.7.709

[CIT0045] Koenen K, Moffitt TE, Poulton R, Martin J, Caspi A. 2007. Early childhood factors associated with the development of post-traumatic stress disorder: results from a longitudinal birth cohort. Psychol Med. 37:181–192. 17052377 10.1017/S0033291706009019 PMC2254221

[CIT0046] Laurence J. 2011. The effect of ethnic diversity and community disadvantage on social cohesion: A multi-level analysis of social capital and interethnic relations in UK communities. European Sociological Review. 27:70–89.

[CIT0047] Mandhane PJ, Sears MR, Poulton R, Greene JM, Lou WY, Taylor DR, Hancox RJ. 2009. Cats and dogs and the risk of atopy in childhood and adulthood. J Allergy Clin Immunol. 124:745–750.e744. 19703709 10.1016/j.jaci.2009.06.038

[CIT0048] Meier M, Caspi A, Knodt A, Hall W, Ambler A, Harrington H, Hogan S, Houts R, Poulton R, Ramrakha S, et al. 2022. Long-term cannabis users show lower cognitive reserves and smaller hippocampal volume in mid-life. Am J Psychiatry. 179:362–374. 35255711 10.1176/appi.ajp.2021.21060664 PMC9426660

[CIT0049] Meier MH, Caspi A, Ambler A, Harrington HL, Houts R, Keefe R, McDonald K, Ward A, Poulton R, Moffitt TE. 2012. Persistent cannabis users chow neuropsychological decline from childhood to midlife. PNAS. 109:E2657–E2664. 22927402 10.1073/pnas.1206820109 PMC3479587

[CIT0050] Meier MH, Shalev I, Moffitt TE, Kapur S, Keefe R, Wong TY, Belsky DW, Harrington HL, Hogan S, Houts R, et al. 2013. Microvascular abnormality in schizophrenia as shown by retinal imaging. Am J Psychiatry. 170:1451–1459. 24030514 10.1176/appi.ajp.2013.13020234 PMC3857729

[CIT0051] Meredith-Jones K, Taylor R, Brown R, McLay-Cooke R, Vlietstra L, Manning P, Poulton R, Haszard J. 2021. Age- and sex-specific visceral fat reference cutoffs and their association with cardio-metabolic risk. IJO. 45:808–817. 10.1038/s41366-021-00743-3 33473174

[CIT0052] Milne BJ, Poulton R, Caspi A, Moffitt TE. 2001. Brain drain or OE? Characteristics of young New Zealanders who leave. NZ Med J. 114:450–453. 11700773

[CIT0053] Moffitt TE. 1993. Adolescent-limited and life-course-persistent antisocial behavior: A developmental taxonomy. Psychol Rev. 100:674–701. 8255953

[CIT0054] Moffitt TE. 2018. Male antisocial behaviour in adolescence and beyond. Nat Hum Behav. 2:177–186. 30271880 PMC6157602

[CIT0055] Moffitt TE, Arseneault L, Belsky DW, Dickson N, Hancox RJ, Harrington HL, Houts R, Poulton R, Roberts BW, Ross S, et al. 2011. A gradient of childhood self-control predicts health, wealth, and public safety. PNAS. 108:2693–2698. 21262822 10.1073/pnas.1010076108 PMC3041102

[CIT0056] Moffitt TE, Belsky DW, Danese A, Poulton R, Caspi A. 2017. The longitudinal study of aging in human young adults: knowledge gaps and research agenda. J Gerontol A Biol Sci Med Sci. 72:210–215. 28087676 10.1093/gerona/glw191 PMC5233916

[CIT0057] Moffitt TE, Caspi A, Ambler A, Bourassa K, Harrington H, Hogan S, Houts R, Ramrakha S, Wood SL, Poulton R. 2022. Deep-seated psychological histories of COVID-19 vaccine hesitance and resistance. PNAS Nexus. 0:1–11. 10.1093/pnasnexus/pgac034 PMC9245853 35783503

[CIT0058] New Zealand Treasury. 2021. The living standards framework 2021. Wellington, New Zealand: The Treasury.

[CIT0059] Odgers CL, Caspi A, Broadbent JM, Dickson N, Hancox RJ, Harrington HL, Poulton R, Sears MR, Thomson WM, Moffitt TE. 2007. Prediction of differential adult health burden by conduct problem subtypes in males. Arch Gen Psych. 64:476–484. 10.1001/archpsyc.64.4.476 17404124

[CIT0060] Odgers CL, Caspi A, Poulton R, Harrington HL, Thomson WM, Broadbent JM, Hancox RJ, Dickson N, Paul C, Moffitt TE. 2008. Female and male antisocial trajectories: from childhood origins to adult outcomes. Dev Psychopathol. 20:673–716. 18423100 10.1017/S0954579408000333

[CIT0061] OECD. 2011a. Perspectives on global development 2012: social cohesion in a shifting world. Paris: OECD Publishing.

[CIT0062] OECD. 2011b. Society at a glance: Asia/pacific 2011. Paris: OECD Publishing.

[CIT0063] Olliver SJ, Broadbent JM, Prasad S, Cai C, Thomson WM, Farella M. 2022. Changes in incisor relationship over the life course – findings from a cohort study. J. Dent. 117:103919. 34896441 10.1016/j.jdent.2021.103919

[CIT0064] Pearson H. 2016. The life project: The untold story of how a group of mavericks, midwives and pioneers changed the lives of everyone in Britain. United Kingdom: Penguin, Random House.

[CIT0065] Poulton R, Caspi A, Milne BJ, Thomson WM, Taylor A, Sears MR, Moffitt TE. 2002. Association between children's experience of socioeconomic disadvantage and adult health: a life-course study. Lancet. 360:1640–1645. 12457787 10.1016/S0140-6736(02)11602-3 PMC3752775

[CIT0066] Poulton R, Caspi A, Moffitt TE, Cannon M, Murray R, Harrington HL. 2000. Children's self-reported psychotic symptoms and adult schizophreniform disorder: a 15-year longitudinal study. Arch Gen Psych. 57:1053–1058. 10.1001/archpsyc.57.11.1053 11074871

[CIT0067] Poulton R, Hancox RJ, Milne BJ, Baxter J, Scott K, Wilson N. 2006. The Dunedin Multidisciplinary Health and Development Study: are its findings consistent with the overall New Zealand population? NZ Med J. 119:U2002. 16751825

[CIT0068] Poulton R, Moffitt TE, Silva PA. 2015. The Dunedin Multidisciplinary Health and Development Study: overview of the first 40 years, with an eye to the future. Soc Psychiatry Psychiatr Epidemiol. 50:679–693. 25835958 10.1007/s00127-015-1048-8 PMC4412685

[CIT0069] Poulton R, Robertson K, Boden J, Horwood J, Theodore R, Potiki T, Ambler A. 2020. Patterns of recreational cannabis use in Aotearoa New Zealand and their consequences: evidence to inform voters in the 2020 referendum. J R Soc N Z. 50:348–365.

[CIT0070] Rasmussen LJH, Caspi A, Ambler A, Broadbent JM, Cohen HJ, d’Arbeloff T, Elliott M, Hancox RJ, Harrington H, Hogan S, Houts R, et al. 2019. Association of neurocognitive and physical function with gait speed in midlife. JAMA Network Open. 2:e1913123. 31603488 10.1001/jamanetworkopen.2019.13123 PMC6804027

[CIT0071] Reeves J, Treharne GJ, Ratima M, Theodore R, Edwards W, Poulton R. 2022. Understanding the privacy concerns of participants in longitudinal studies: A summary of findings from interviews with members of the Dunedin study. Dunedin: University of Otago.

[CIT0072] Reeves J, Treharne GJ, Theodore R, Edwards W, Ratima M, Poulton R. 2021. Understanding the data-sharing debate in the context of Aotearoa/New Zealand: A narrative review on the perspectives of funders, publishers/journals, researchers, participants, and Maori collectives. Kotuitui: N J Soc Sci Online. 17:1–23.

[CIT0073] Reuben A, Caspi A, Belsky DW, Broadbent J, Harrington H, Sugden K, Houts RM, Ramrakha S, Poulton R, Moffitt TE. 2017. Association of childhood blood lead levels with cognitive function and socioeconomic status at age 38 years and with IQ change and socioeconomic mobility between childhood and adulthood. JAMA. 317:1244–1251. 28350927 10.1001/jama.2017.1712 PMC5490376

[CIT0074] Reuben A, Elliott ML, Abraham C, Broadbent J, Houts RM, Ireland D, Knodt AR, Poulton R, Ramrakha S, Hariri AR, et al. 2020. Association of childhood lead exposure with MRI measurements of structural brain integrity in midlife. JAMA. 324:1970–1979. 33201203 10.1001/jama.2020.19998 PMC7672511

[CIT0075] Reuben A, Moffitt TE, Abraham CW, Ambler A, Elliott ML, Hariri AR, Harrington H, Hogan S, Houts RM, Ireland D, et al. In Press. Risk indices for Alzheimer’s disease and related dementias are informative about brain health in midlife. 10.1093/braincomms/fcac223 PMC9535507 36213312

[CIT0076] Reuben A, Moffitt TE, Caspi A, Belsky DW, Harrington H, Schroeder F, Hogan S, Ramrakha S, Poulton R, Danese A. 2016. Lest we forget: comparing retrospective and prospective assessments of adverse childhood experiences in the prediction of adult health. J Child Psychol Psychiatry. 57:1103–1112. 27647050 10.1111/jcpp.12621 PMC5234278

[CIT0077] Reuben A, Schaefer J, Moffitt T, Broadbent J, Harrington H, Houts R, Ramrakha S, Poulton R, Caspi A. 2019. Association of childhood lead exposure with adult personality traits and lifelong mental health. JAMA Psychiatry. 76:418–425. 30673063 10.1001/jamapsychiatry.2018.4192 PMC6450277

[CIT0078] Richards R, Poulton R, Reeder AI, Williams SM. 2009. Childhood and contemporaneous correlates of adolescent physical inactivity: a longitudinal study. J Adolesc Health. 44:260–267. 19237112 10.1016/j.jadohealth.2008.08.005 PMC2728043

[CIT0079] Richmond-Rakerd LS, D’Souza S, Andersen SH, Hogan S, Houts RM, Poulton R, Ramrakha S, Caspi A, Milne BJ, Moffitt TE. 2020. Clustering of health, crime and social-welfare inequality in 4 million citizens from two nations. Nat Hum Behav. 4:255–264. 31959926 10.1038/s41562-019-0810-4 PMC7082196

[CIT0080] Rocha TB, Fisher HL, Caye A, Anselmi L, Arseneault L, Barros FC, Caspi A, Danese A, Goncalves H, Harrington H, et al. 2021. Identifying adolescents at risk for depression: a prediction score performance in cohorts based in three different continents. J Am Acad Child Adolesc Psychiatry. 60:262–273. 31953186 10.1016/j.jaac.2019.12.004 PMC8215370

[CIT0081] Romer A, Elliott ML, Knodt A, Sison M, Ireland D, Houts R, Ramrakha S, Poulton R, Keenan R, Melzer T, et al. 2021. Pervasively thinner neocortex as a transdiagnostic feature of psychopathology. Am J Psychiatry. 178:174–182. 32600153 10.1176/appi.ajp.2020.19090934 PMC7772268

[CIT0082] Schaefer JD, Caspi A, Belsky DW, Harrington H, Houts R, Horwood LJ, Hussong A, Ramrakha S, Poulton R, Moffitt TE. 2017. Enduring mental health: prevalence and prediction. J Abnorm Psychol. 126:212–224. 27929304 10.1037/abn0000232 PMC5304549

[CIT0083] Science News. 2022. Century of Science: 100 years of psychology studies that have tried to make sense of the mind. https://www.sciencenews.org/century/psychology-mind-humans-mental-health

[CIT0084] Sears MR, Greene JM, Willan A, Taylor DR, Flannery EM, Cowan JO, Herbison GP, Poulton R. 2002. Long-term relation between breast-feeding and development of atopy and asthma in children and young adults: a longitudinal study. Lancet. 360:901–907. 12354471 10.1016/S0140-6736(02)11025-7

[CIT0085] Sears MR, Greene JM, Willan A, Wiecek E, Taylor DR, Flannery EM, Cowan JO, Herbison GP, Silva PA, Poulton R. 2003. A longitudinal population-based cohort study of childhood asthma followed to adulthood. NEJM. 349:1414–1422. 14534334 10.1056/NEJMoa022363

[CIT0086] Shalev I, Moffitt TE, Wong TY, Meier MH, Houts R, Ding J, Cheung CYL, Ikram MK, Caspi A, Poulton R. 2013. Retinal vessel caliber and lifelong neuropsychological functioning: retinal imaging as an investigative tool for cognitive epidemiology. Psychol Sci. 24:1198–1207. 23678508 10.1177/0956797612470959 PMC3713191

[CIT0087] Simpson A, Kirkland C, Silva PA. 1984. Vision and eye problems in seven year olds: A report from the Dunedin Multidisciplinary Health and Development Research unit. N Z Med J. 97:445–449. 6589540

[CIT0088] Singh A, Gale J, Cheyne K, Ambler A, Ramrakha S, Poulton R, Wilson G. 2022. The prevalence of glaucoma in 45-year-old New Zealanders. NZ Med Journal. 135:35–42. 35728203

[CIT0089] Spoonley P, Peace R, Butcher A, O’Neill D. 2005. Social cohesion: A policy and indicator framework for assessing immigrant and host outcomes. Social Policy Journal of New Zealand. 24:85–110.

[CIT0090] Sugden K, Hannon EJ, Arseneault L, Belsky DW, Corcoran DL, Fisher HL, Houts RM, Kandaswamy R, Moffitt TE, Poulton R, et al. 2020. Patterns of reliability: assessing the reproducibility and integrity of DNA methylation measurement. Patterns. 1:100014. 32885222 10.1016/j.patter.2020.100014 PMC7467214

[CIT0091] Talley NJ, Howell S, Poulton R. 2001. The irritable bowel syndrome and psychiatric disorders in the community: is there a link? Am J Gastroenterology. 96:1072–1079. 10.1111/j.1572-0241.2001.03741.x 11316149

[CIT0092] Talley NJ, Howell S, Poulton R. 2004. Obesity and chronic gastrointestinal tract symptoms in young adults: a birth cohort study. Am J Gastroenterology. 99:1807–1814. 10.1111/j.1572-0241.2004.30388.x 15330923

[CIT0093] Theodore R, Broadbent JM, Nagin DS, Ambler A, Hogan S, Ramrakha S, Cutfield W, Williams MJA, Harrington HL, Moffitt TE, et al. 2015. Systolic blood pressure trajectories from childhood to early mid-life: early life predictors, effect modifiers, and adult cardiovascular outcomes. Hypertension. 66:1108–1115. 26558818 10.1161/HYPERTENSIONAHA.115.05831 PMC4646716

[CIT0094] Thomson WM, Broadbent JM, Locker D, Poulton R. 2009. Trajectories of dental anxiety in a birth cohort. Community Dent Oral Epidemiol. 37:209–219. 19508269 10.1111/j.1600-0528.2009.00473.x PMC2817941

[CIT0095] Thomson WM, Poulton R, Broadbent JM, Moffitt TE, Caspi A, Beck JD, Welch D, Hancox RJ. 2008. Cannabis smoking and periodontal disease among young adults. JAMA. 299:525–531. 18252882 10.1001/jama.299.5.525 PMC2823391

[CIT0096] Thomson WM, Poulton R, Kruger E, Boyd D. 2000. Socio–economic and behavioural risk factors for tooth loss from age 18 to 26 among participants in the Dunedin Multidisciplinary Health and Development Study. Caries Res. 34:361–366. 11014902 10.1159/000016610

[CIT0097] Thomson WM, Poulton R, Milne BJ, Caspi A, Broughton JR, Ayers KMS. 2004. Socio-economic inequalities in oral health in childhood and adulthood in a birth cohort. Community Dent Oral Epidemiol. 32:345–353. 15341619 10.1111/j.1600-0528.2004.00173.x

[CIT0098] van Roode T, Dickson N, Righarts A, Gillett W. 2015. Cumulative incidence of infertility in a New Zealand birth cohort to age 38 by sex, and the relationship with family formation. Fertil Steril. 103:1053–1058.e2. 25637476 10.1016/j.fertnstert.2014.12.121

[CIT0099] Vergolini L. 2011. Social cohesion in Europe: How do the different dimensions of inequality affect social cohesion? Int J Comp Sociol. 52:197–214.

[CIT0100] Welch D, Dawes PJ. 2007. Variation in the normal hearing threshold predicts childhood IQ, linguistic and behavioural outcomes. Pediatr Res. 61:737–744. 17426656 10.1203/pdr.0b013e31805341c1

[CIT0101] Williams MJA, Milne BJ, Ambler A, Theodore R, Ramrakha S, Caspi A, Moffitt TE, Poulton R. 2017. Childhood body mass index and endothelial dysfunction evaluated by peripheral arterial tonometry in early midlife. IJ O. 41:1355–1360. 10.1038/ijo.2017.108 PMC5585033 28465609

[CIT0102] Wilson GA, Cheyne K, Ramrakha S, Ambler A, Tan GSW, Caspi A, Williams B, Sugden K, Houts R, Niederer RL, et al. 2021. Are macular drusen in midlife a marker of accelerated biological ageing? Clin Exp Optom. 1–6. 34902293 10.1080/08164622.2021.2012428

