# Supplementary material for: The Dunedin study after half a century: reflections on the past, and course for the future
Source: J R Soc N Z. 2022 Sep 7;53(4):446–65. doi: 10.1080/03036758.2022.2114508 (PMC11459797; doi:10.1080/03036758.2022.2114508)
Supplement: Supplemental Material [file TNZR_A_2114508_SM8133.docx]

**The Dunedin Study after half a century:**

**reflections on the past, and course for the future**

**Supplementary Materials**

**1. Dunedin Study data overview**

To provide an overview of the data collected over the first 45 years of the Dunedin Study, Table S1a summarises the observations, tests, administrative records, and parent, teacher, self, and informant-reported measures used at each phase; Table S1b summarises the specific blood biomarkers assessed at each phase.

**Table S1a. Summary of data collected to date in the Dunedin Study, 1972-2022**

| Measure | Method | Age at which domain was assessed | | | | | | | | | | | | | |
| --- | --- | --- | --- | --- | --- | --- | --- | --- | --- | --- | --- | --- | --- | --- | --- |
|  |  | Birth | 3 | 5 | 7 | 9 | 11 | 13 | 15 | 18 | 21 | 26 | 32 | 38 | 45 |
| **Psychosocial measures** |  |  |  |  |  |  |  |  |  |  |  |  |  |  |  |
| Childhood socioeconomic background | 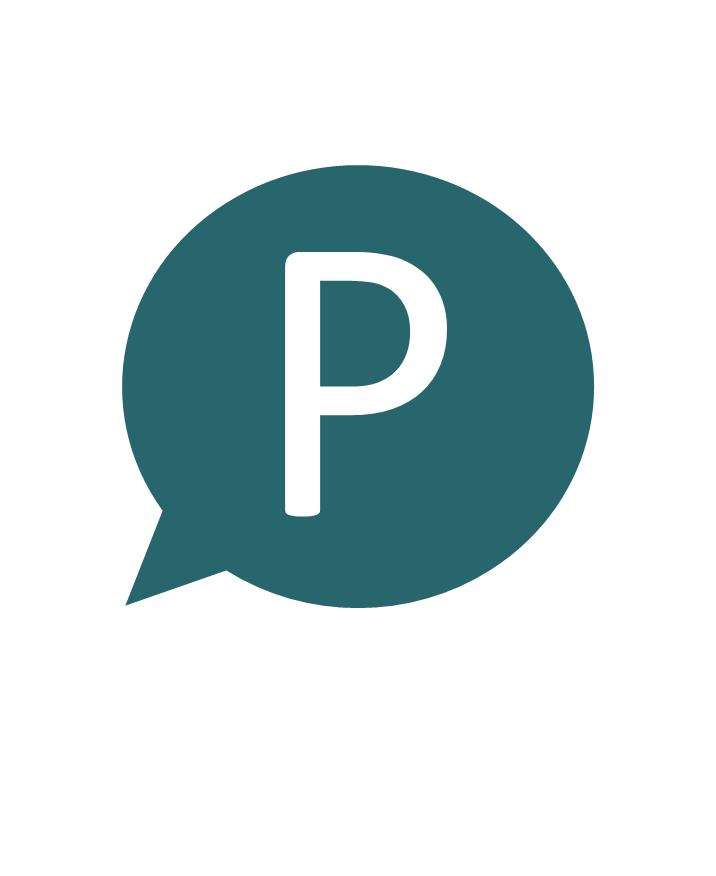 | 0 | 3 | 5 | 7 | 9 | 11 | 13 | 15 |  |  |  |  |  |  |
| Family structure, stability | 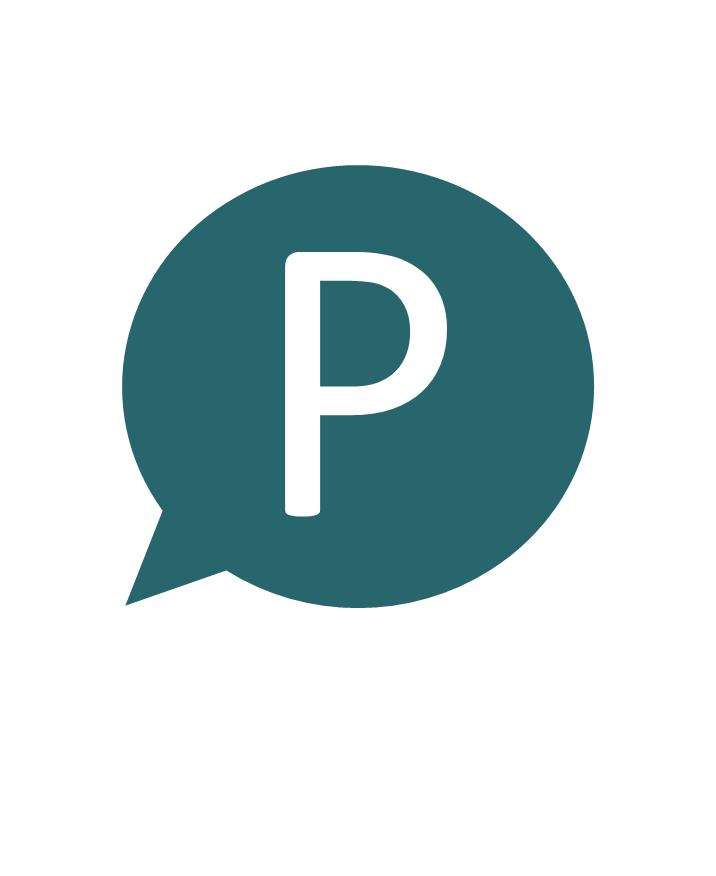 | 0 | 3 | 5 | 7 | 9 | 11 | 13 | 15 |  |  |  |  |  |  |
| Paediatric neurological examinations and motor tests | 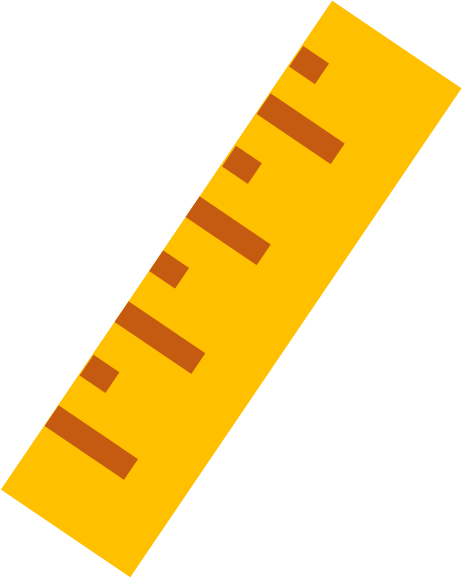 |  | 3 | 5 | 7 | 9 |  |  |  |  |  |  |  |  |  |
| Child temperament | 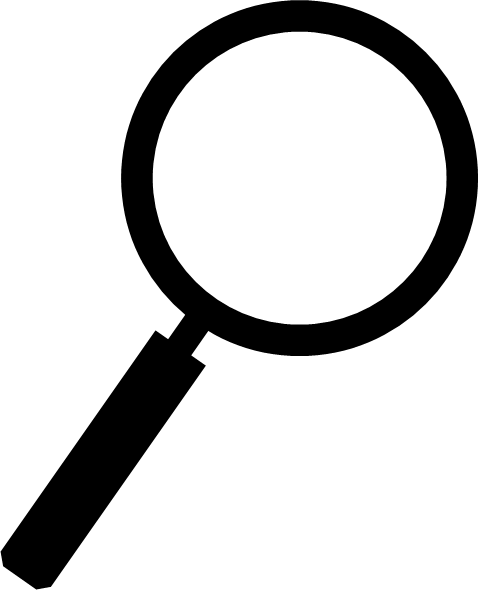 |  | 3 | 5 | 7 | 9 | 11 |  |  |  |  |  |  |  |  |
| Ratings of child’s health, ill-health checklists | 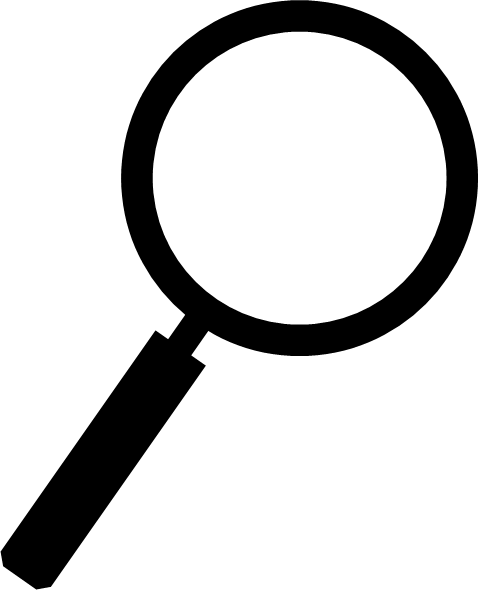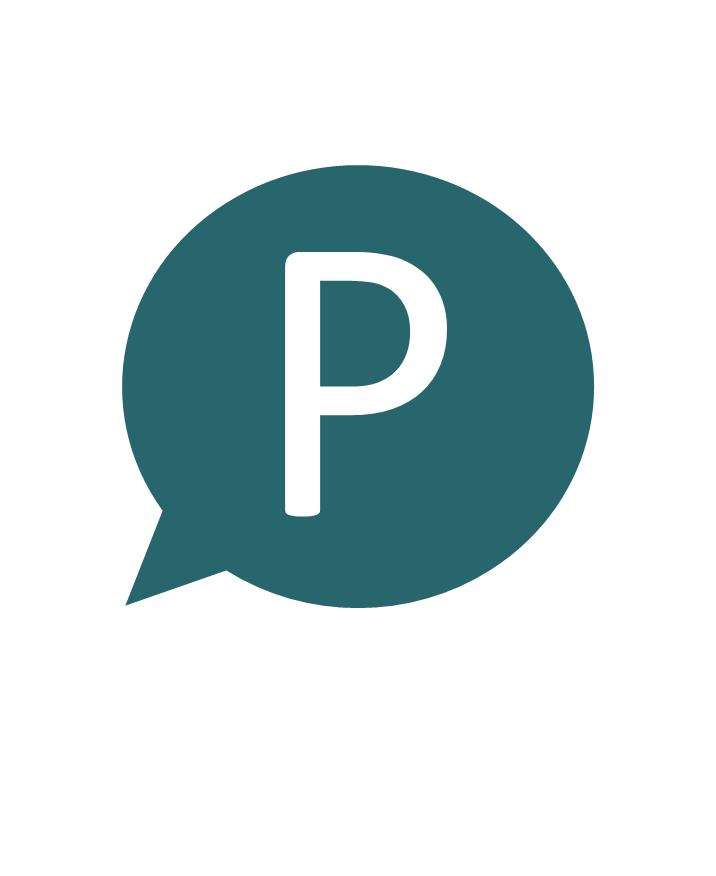 |  | 3 | 5 | 7 | 9 | 11 | 13 | 15 |  |  |  |  |  |  |
| Parental mental health | 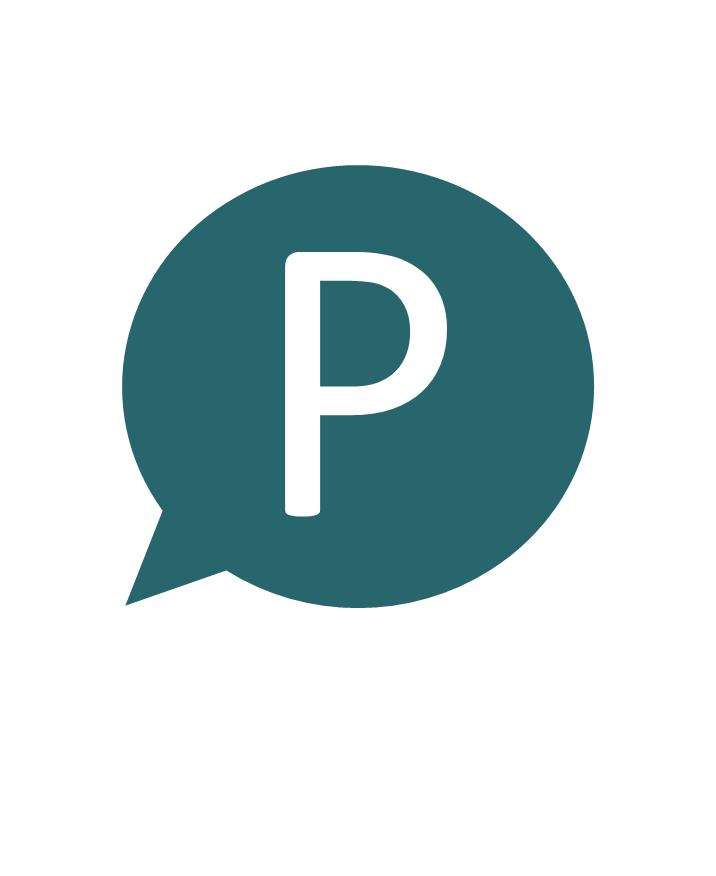 |  | 3 | 5 | 7 | 9 | 11 | 13 | 15 |  |  |  |  |  |  |
| Family functioning, parent–child relationship | 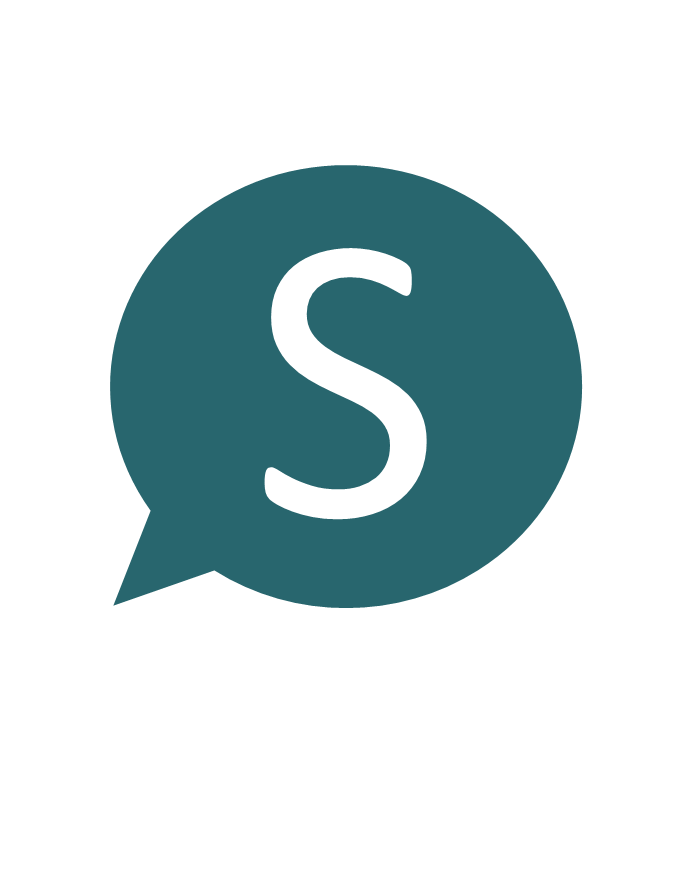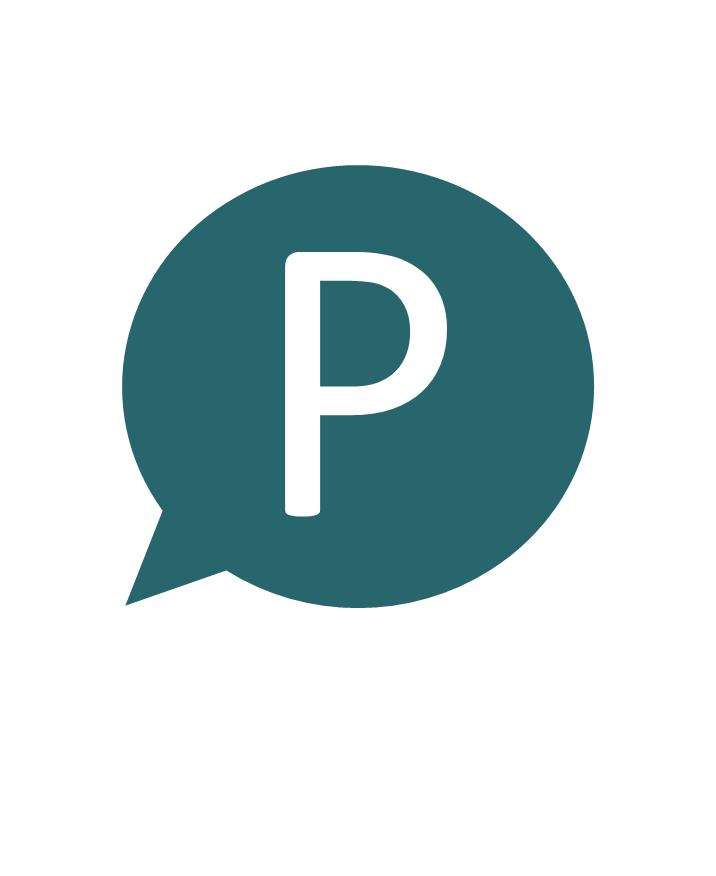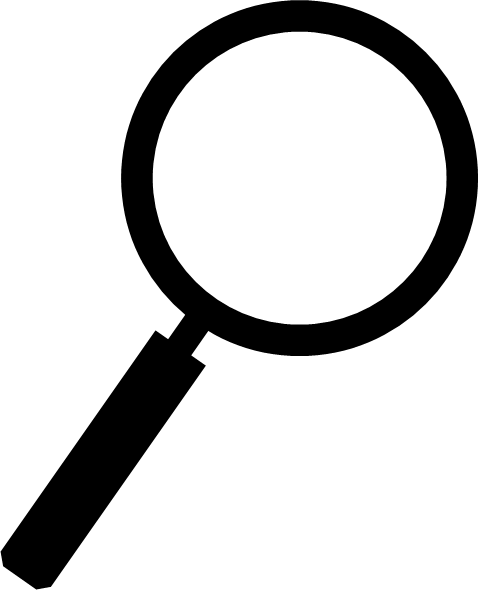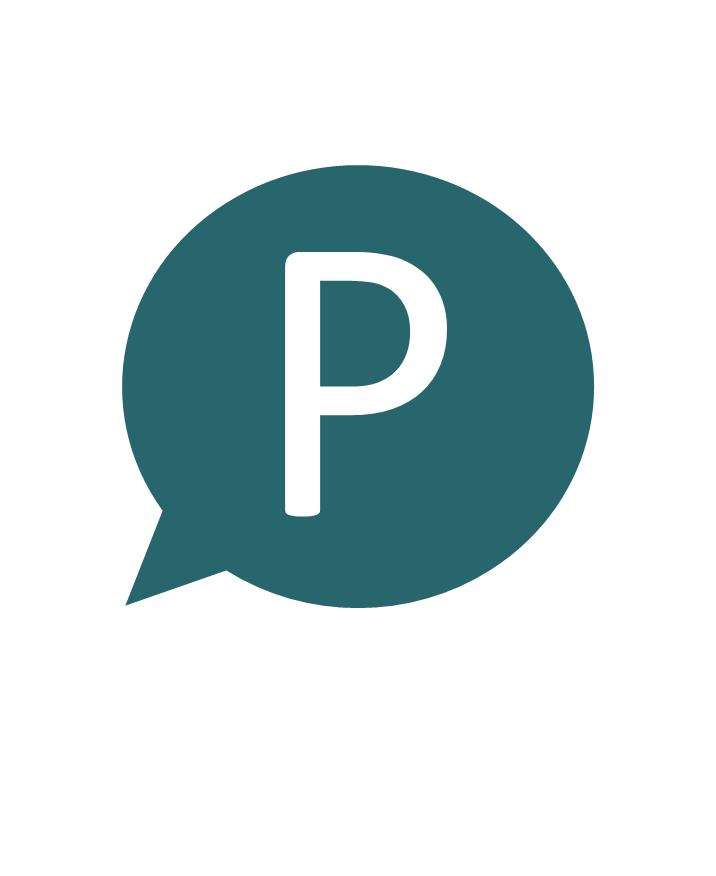 |  | 3 | 5 | 7 | 9 | 11 | 13 | 15 |  |  |  |  |  |  |
| Adverse experiences: abuse, neglect, household dysfunction | 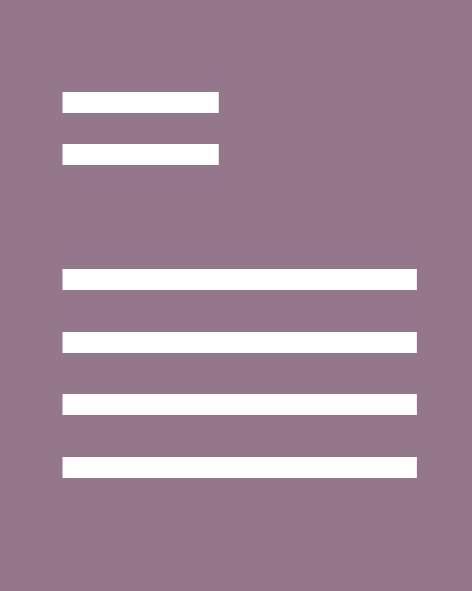 |  | 3 | 5 | 7 | 9 | 11 | 13 | 15 |  |  | 26 |  | 38 |  |
| Teacher/school reports | 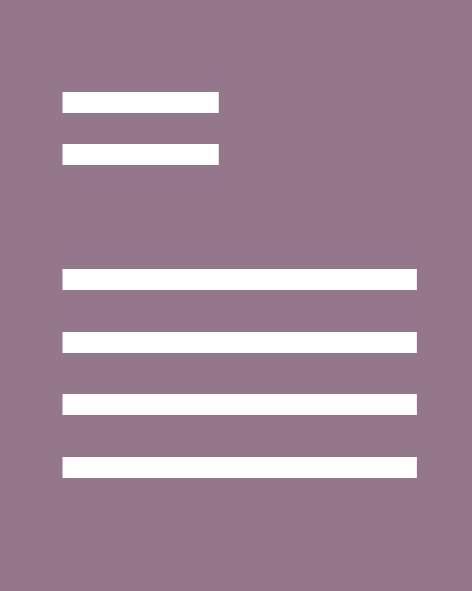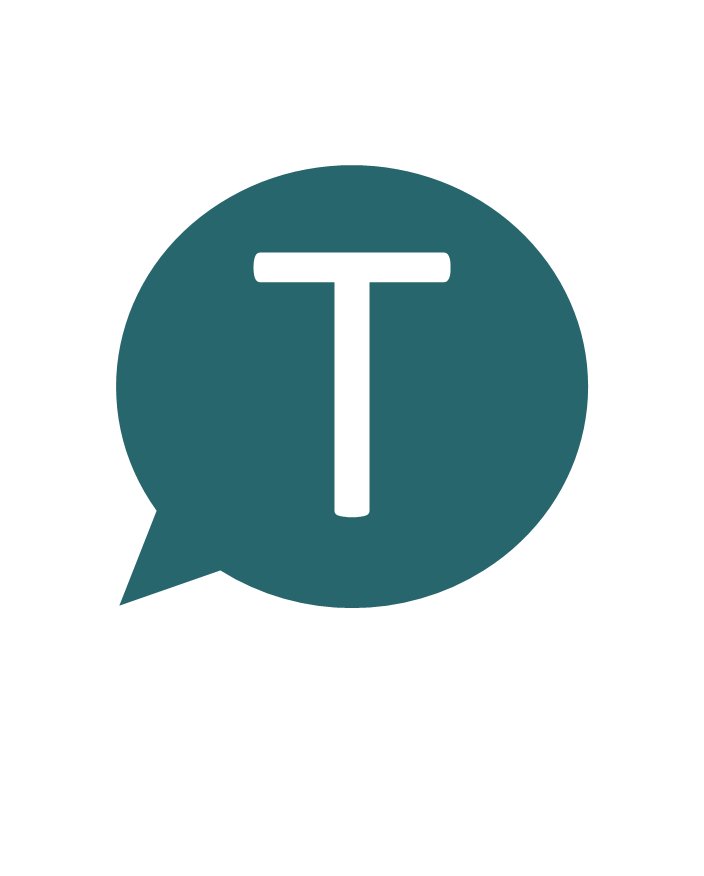 |  |  | 5 | 7 | 9 | 11 | 13 | 15 |  |  |  |  |  |  |
| Peers: rejection by, attachment to, activities with | 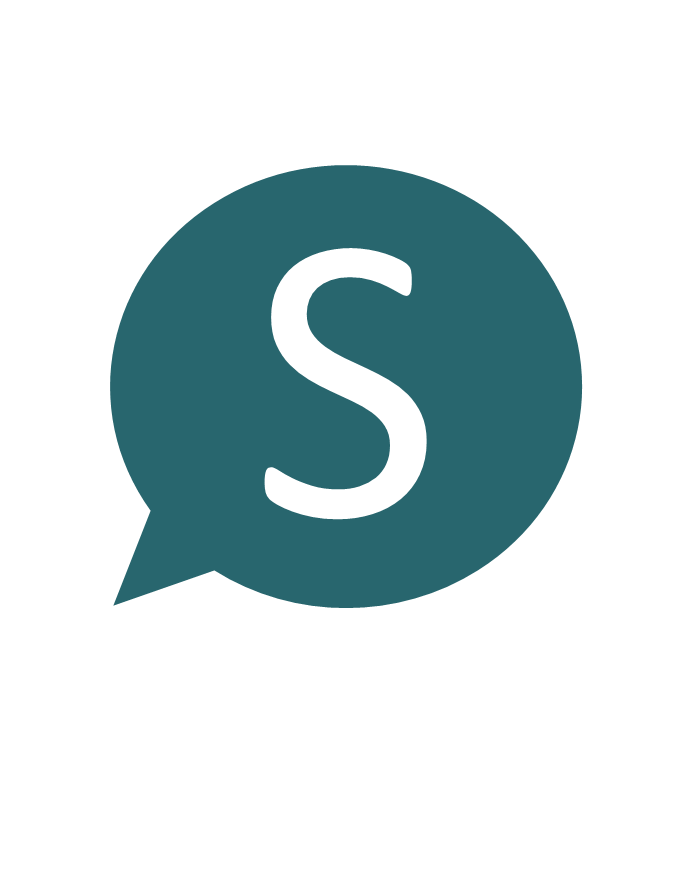 |  |  | 5 | 7 | 9 | 11 | 13 | 15 |  |  |  |  |  |  |
| Pubertal timing: girls’ menarche, boys’ height spurt | 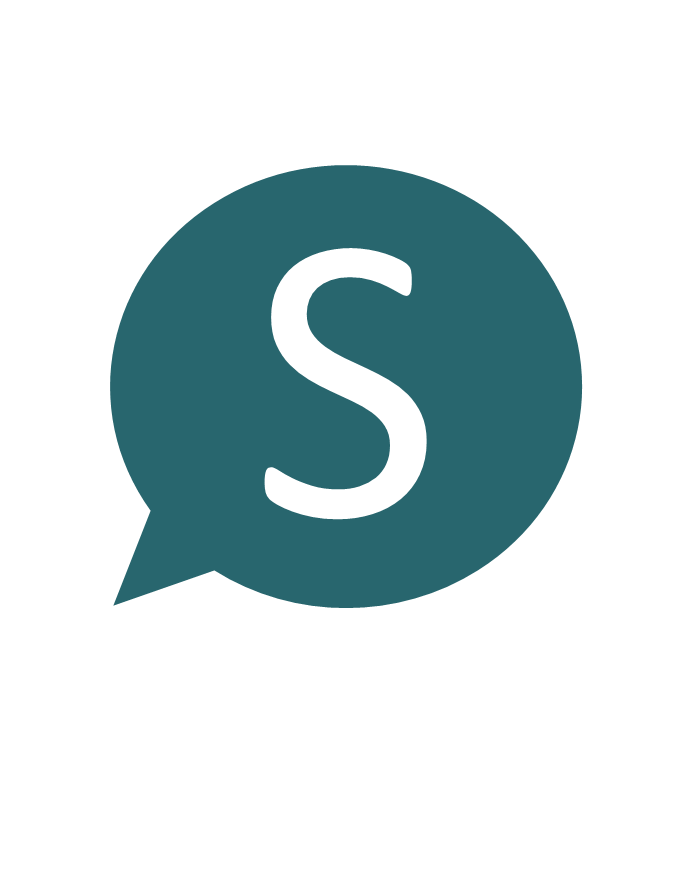 |  |  |  |  | 9 | 11 | 13 | 15 | 18 |  |  |  |  |  |
| Neuropsychological testing, IQ testing, language | 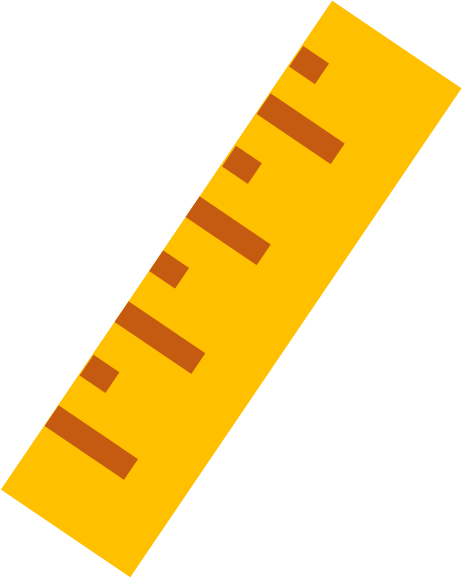 |  | 3 | 5 | 7 | 9 | 11 | 13 |  |  |  |  |  | 38 | 45 |
| Parental loss, familial death, divorce | 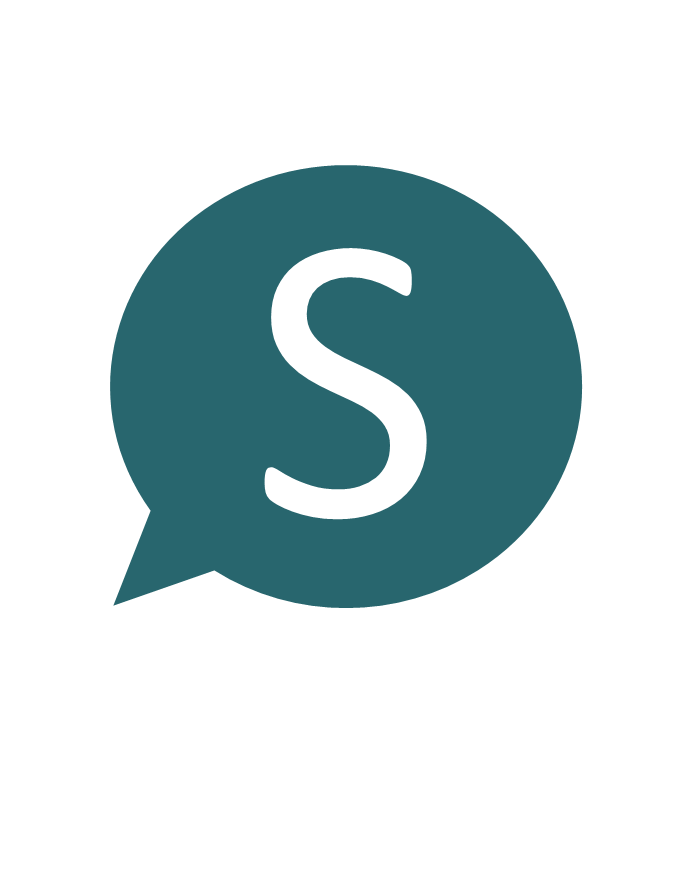 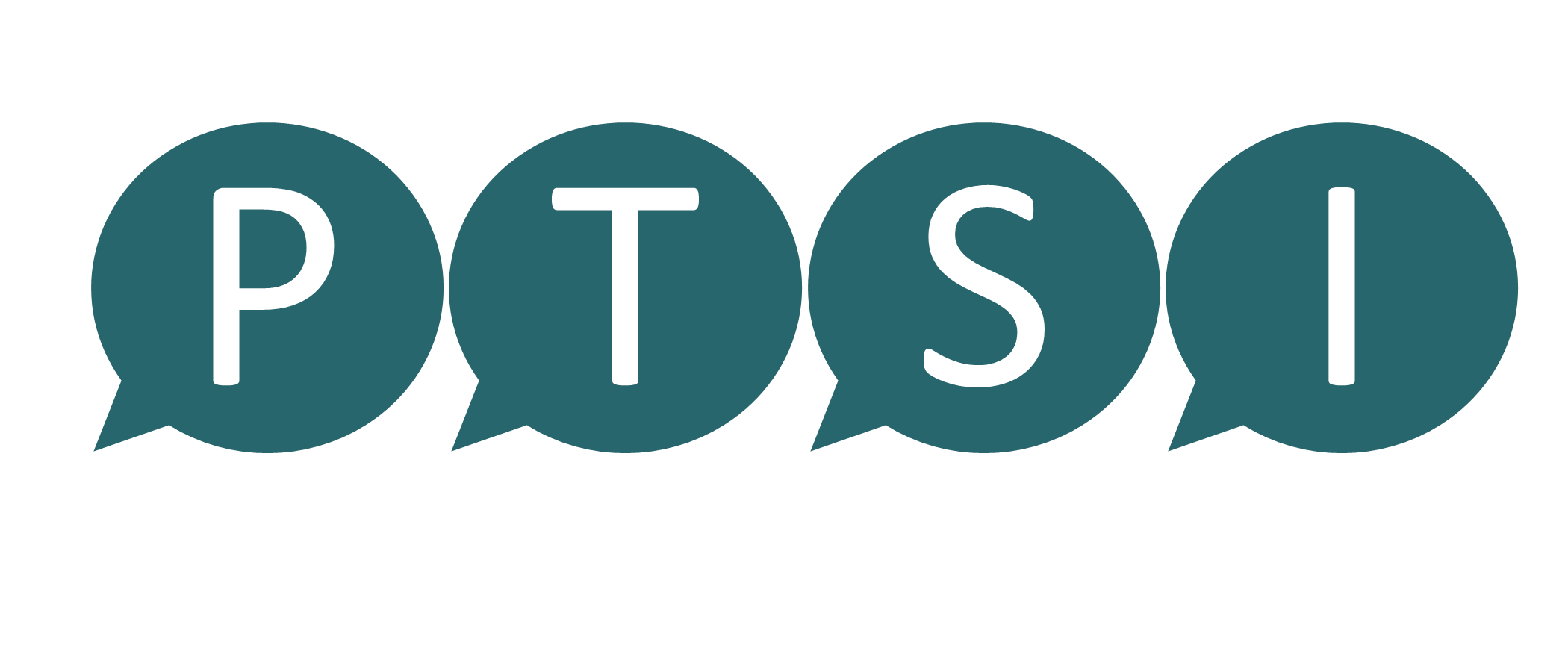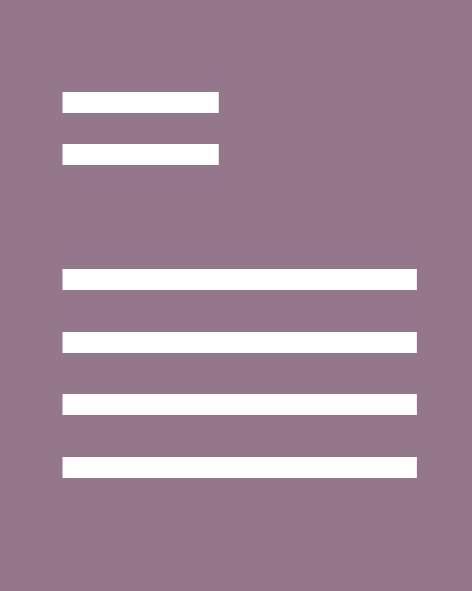 |  | 3 | 5 | 7 | 9 | 11 | 13 | 15 |  |  | 26 |  | 38 | 45 |
| Mental disorders and psychopathology |  |  |  | 5 | 7 | 9 | 11 | 13 | 15 | 18 | 21 | 26 | 32 | 38 | 45 |
| Self-harm and suicide attempts | 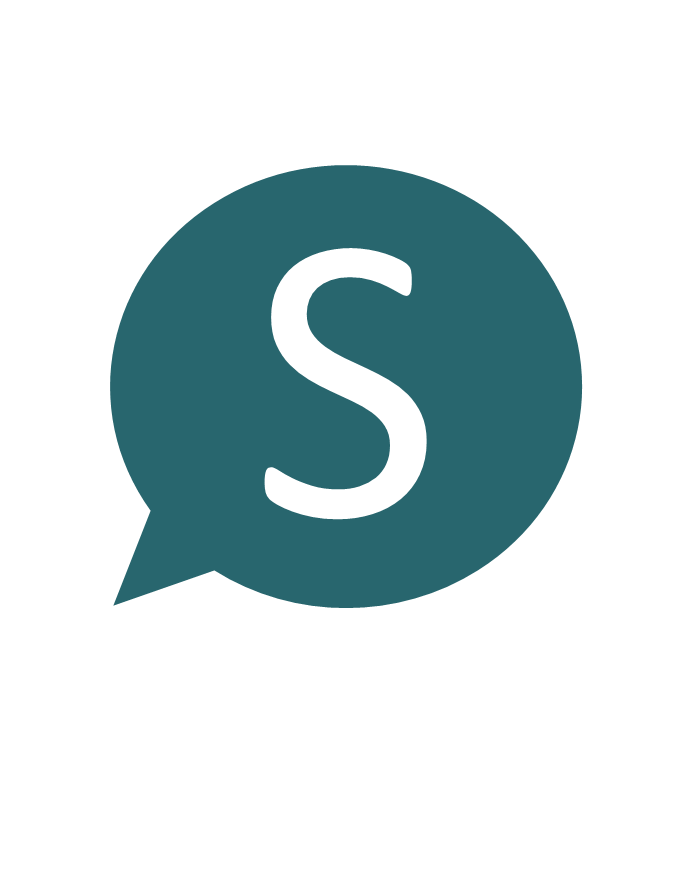 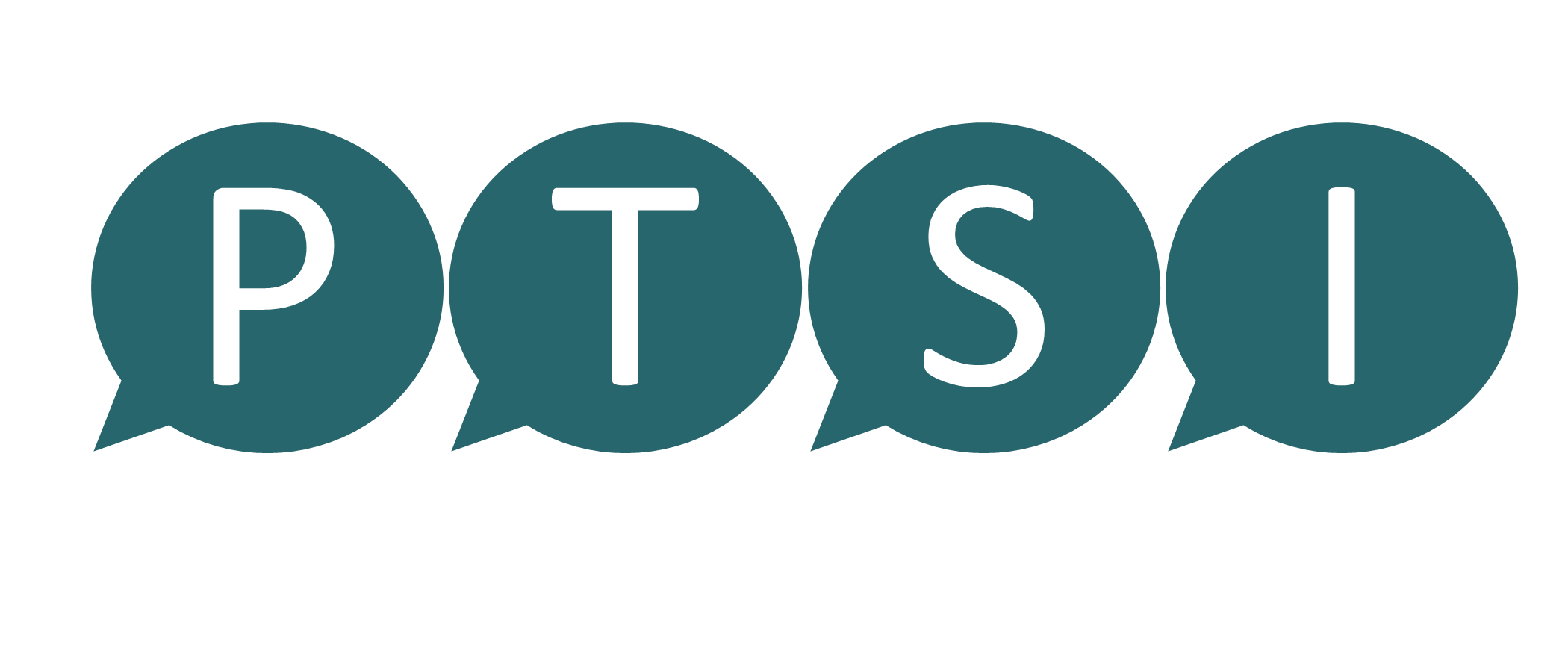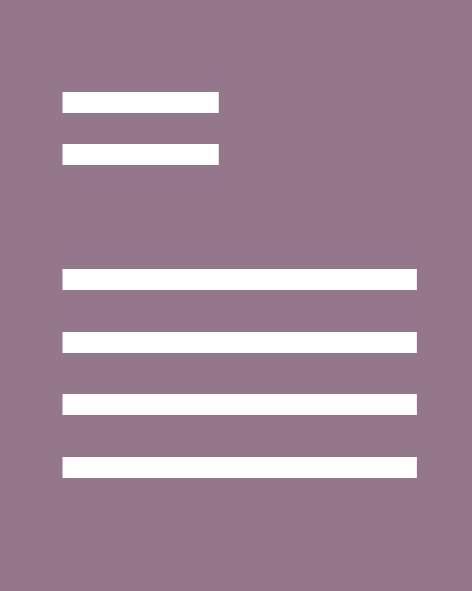 |  |  |  |  |  |  |  | 15 | 18 | 21 | 26 | 32 | 38 | 45 |
| Antisocial behaviours |  |  |  | 5 | 7 | 9 | 11 | 13 | 15 | 18 | 21 | 26 | 32 | 38 | 45 |
| Academic attainment, literacy tests, degrees earned, skills | 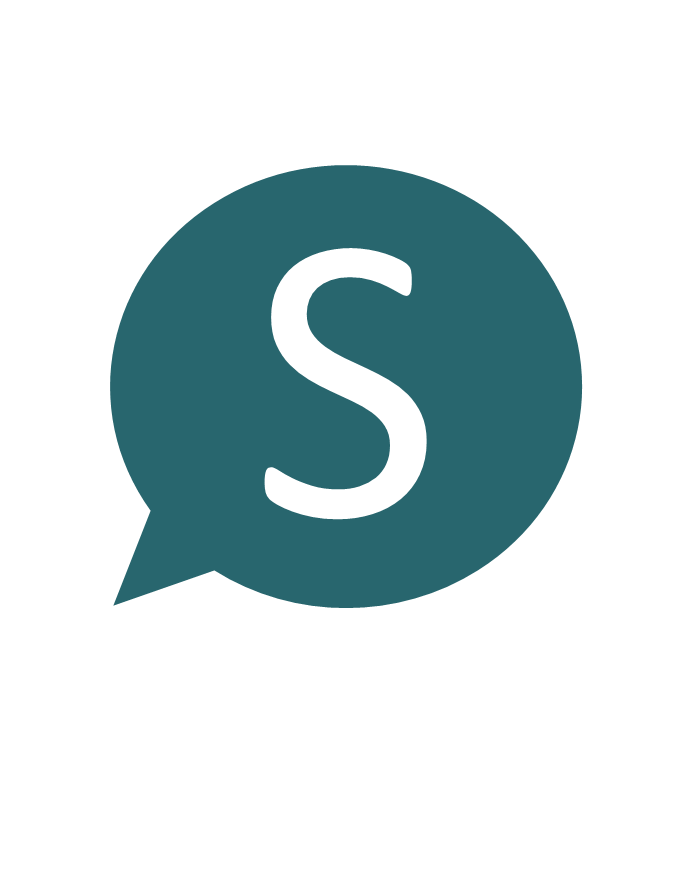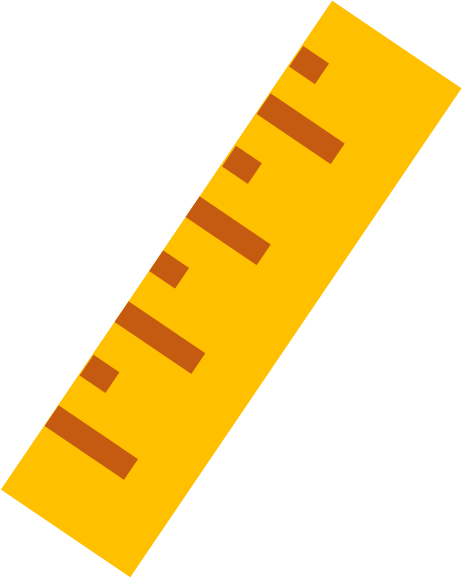 |  |  |  | 7 | 9 | 11 | 13 | 15 | 18 | 21 | 26 | 32 | 38 | 45 |
| Physical exercise/activity | **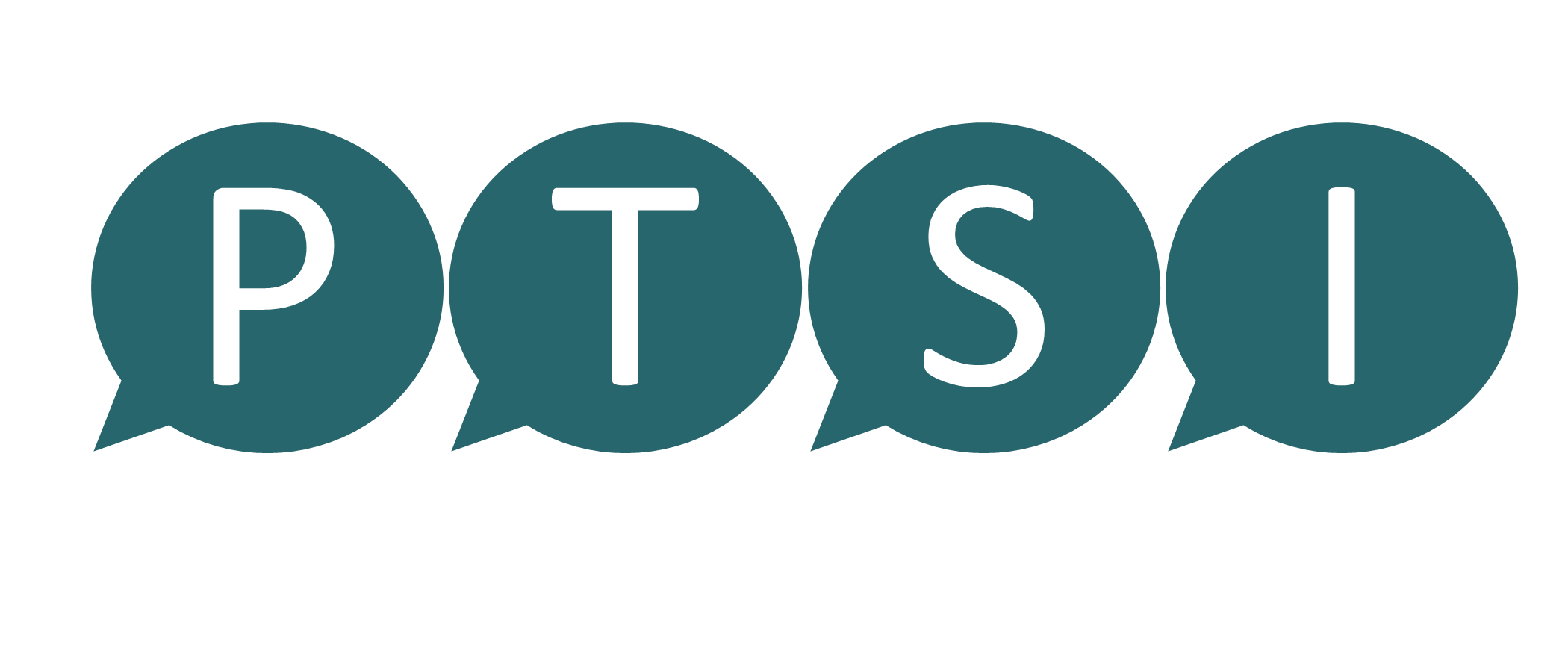**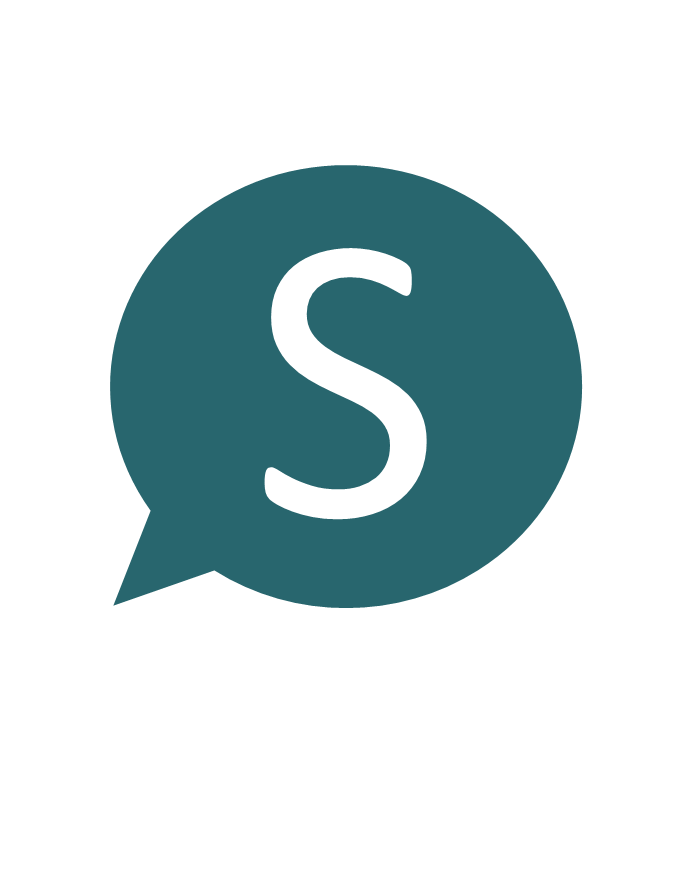 |  |  |  | 7 | 9 | 11 | 13 | 15 | 18 | 21 | 26 | 32 | 38 | 45 |
| Substance abuse, dependence: smoking, alcohol, cannabis, narcotics |  |  |  |  |  |  | 11 | 13 | 15 | 18 | 21 | 26 | 32 | 38 | 45 |
| Personality: Multidimensional Personality Questionnaire and big-5 | 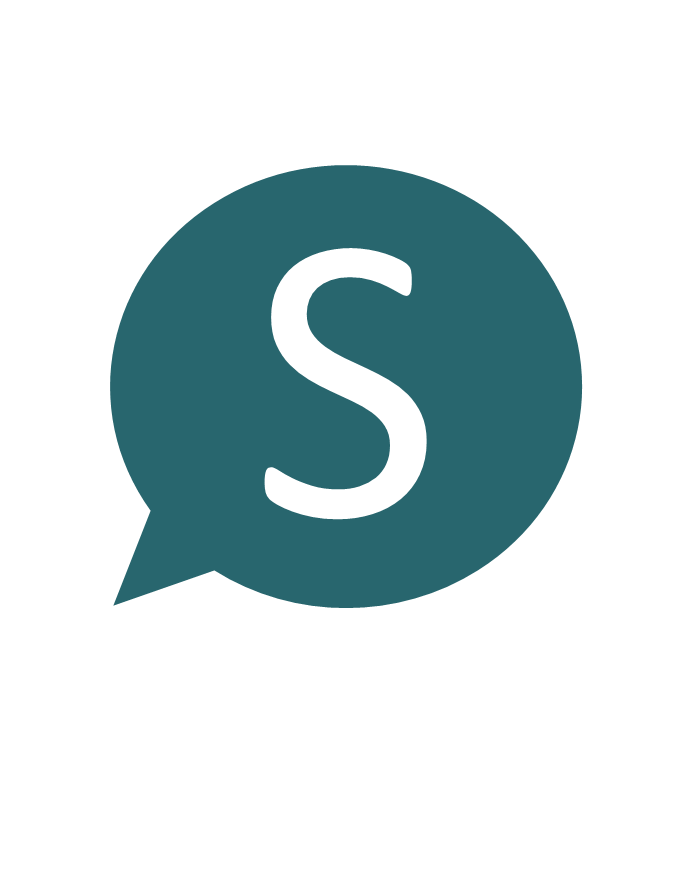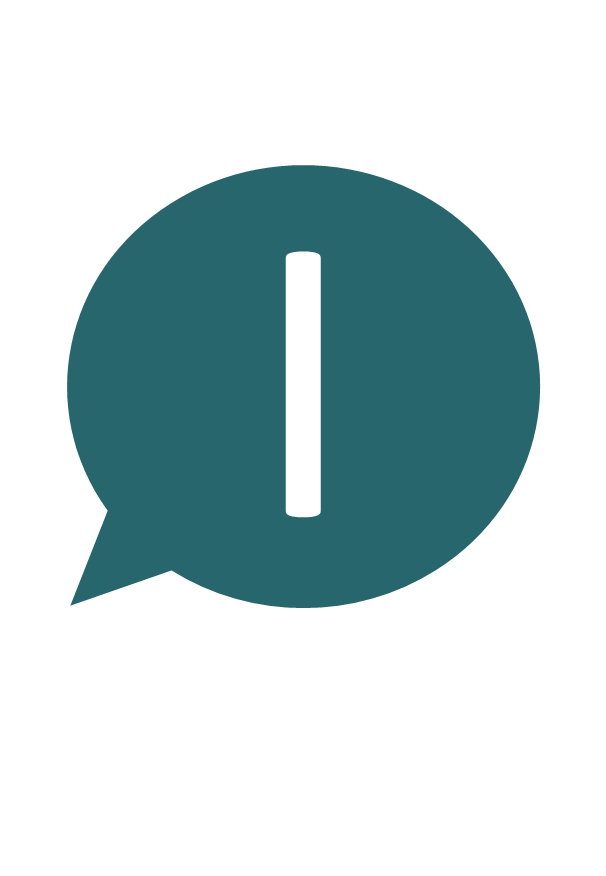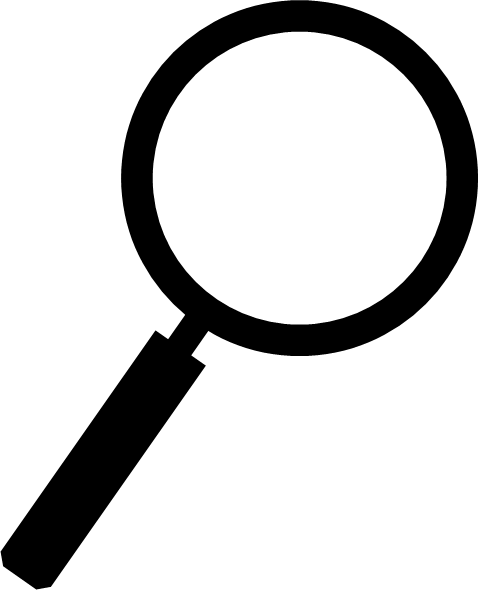 |  |  |  |  |  |  |  |  | 18 |  | 26 | 32 | 38 |  |
| Sexual and reproductive health, behaviour, attitudes | 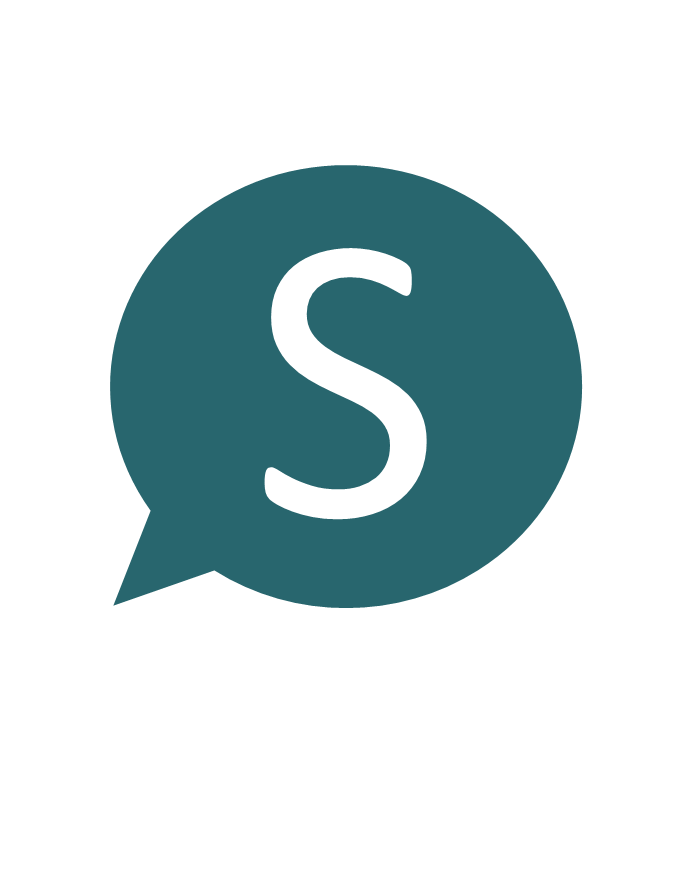 |  |  |  |  |  |  |  |  | 18 | 21 | 26 | 32 | 38 | 45 |
| Intimate partner relationship status, quality, commitment, children | 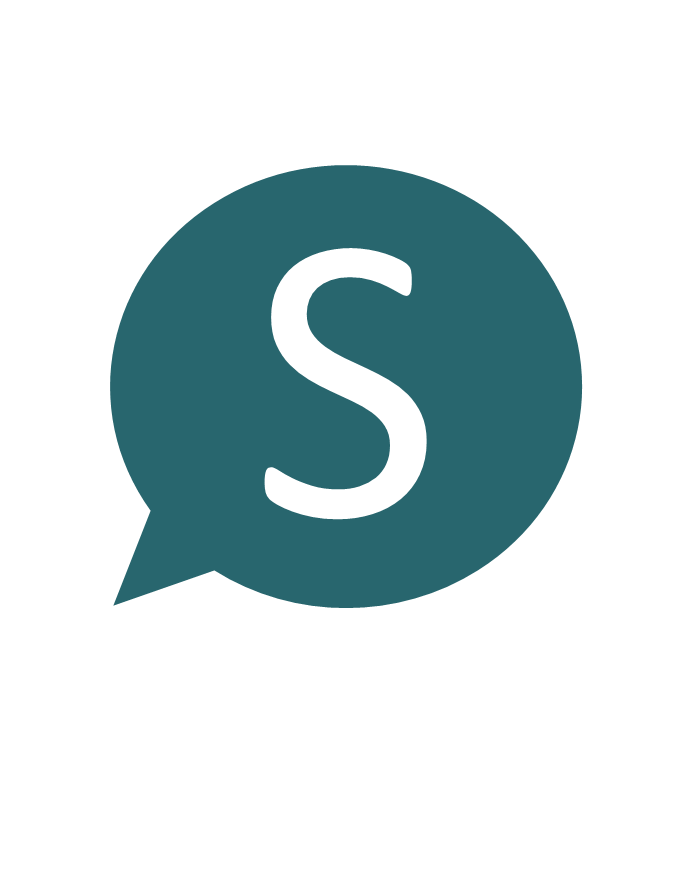 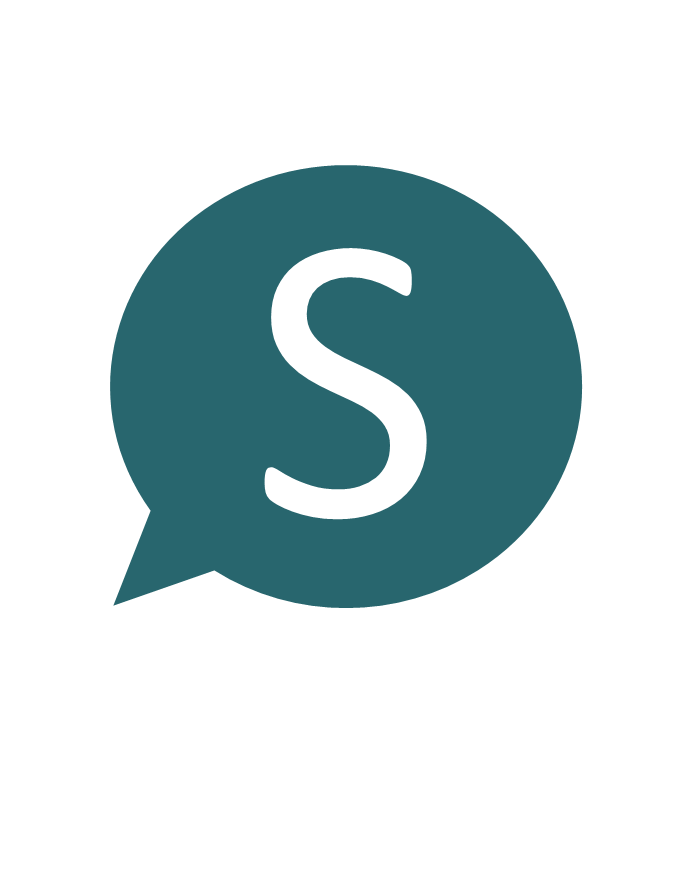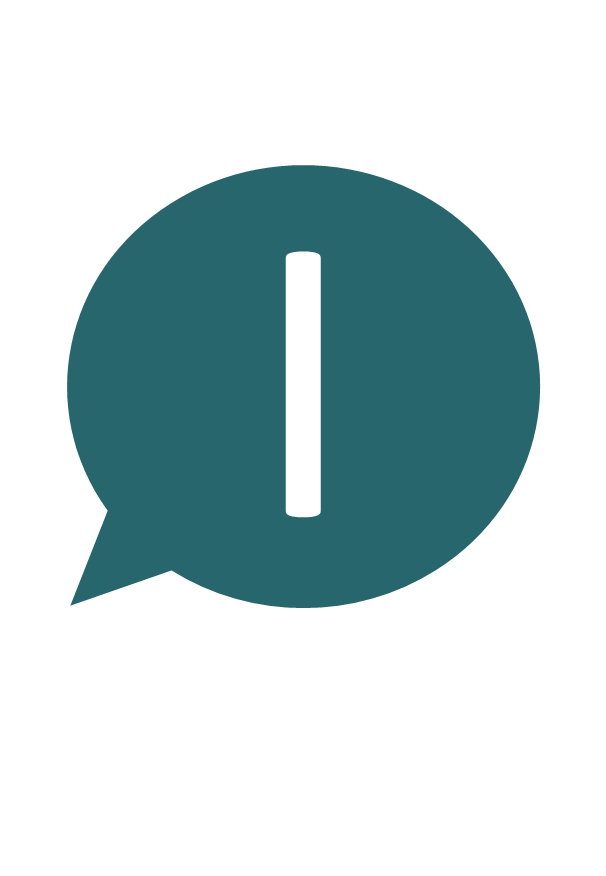 |  |  |  |  |  |  |  |  |  | 21 | 26 | 32 | 38 | 45 |
| Intimate partner violence: psychological and physical |  |  |  |  |  |  |  |  |  |  | 21 | 26 | 32 | 38 | 45 |
| Parent relationship quality | 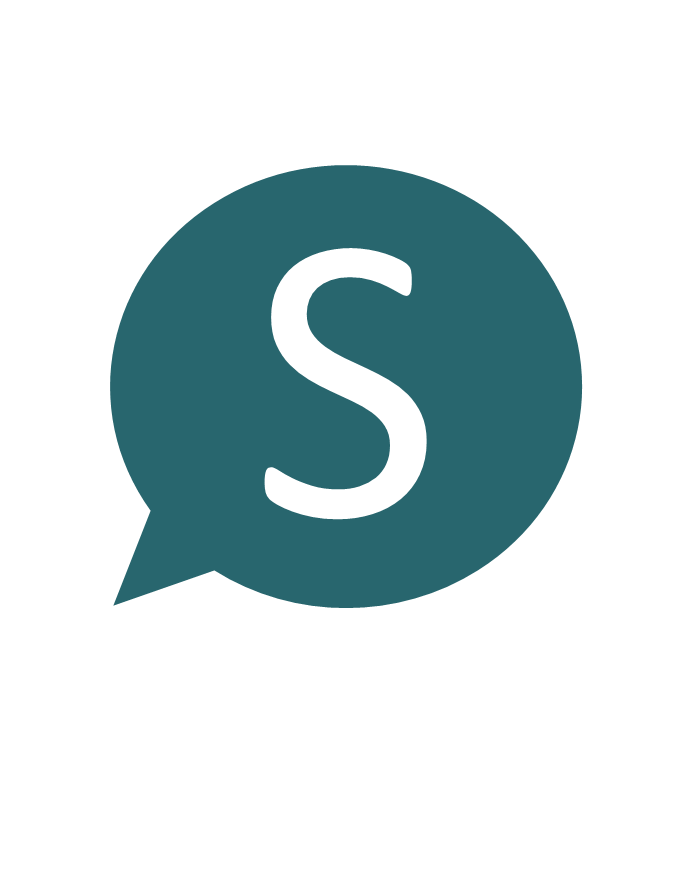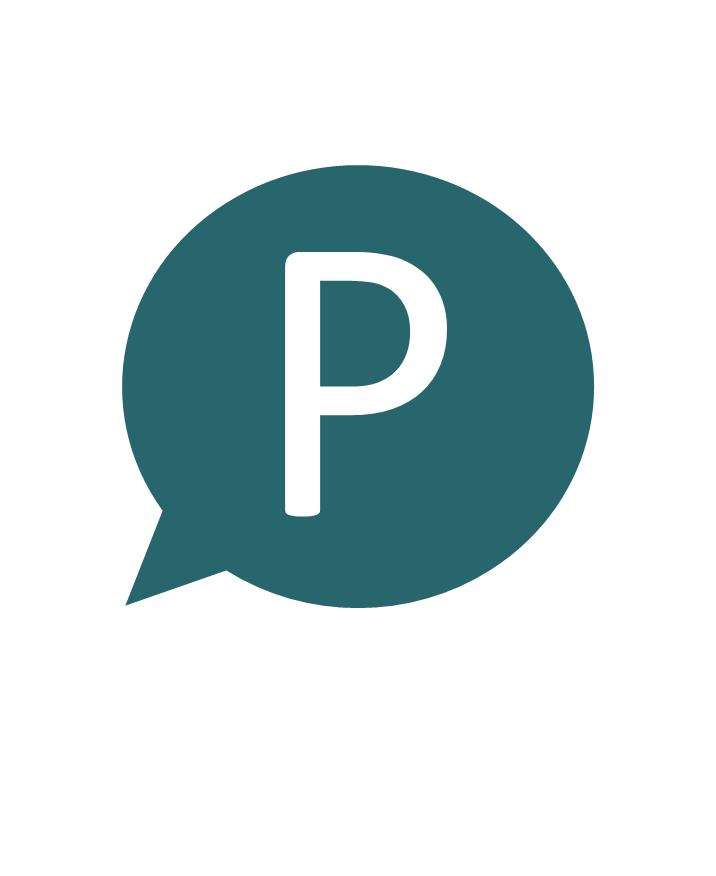 |  |  |  |  |  |  |  |  |  |  | 26 | 32 |  |  |
| Work status, type, characteristics, and experiences | 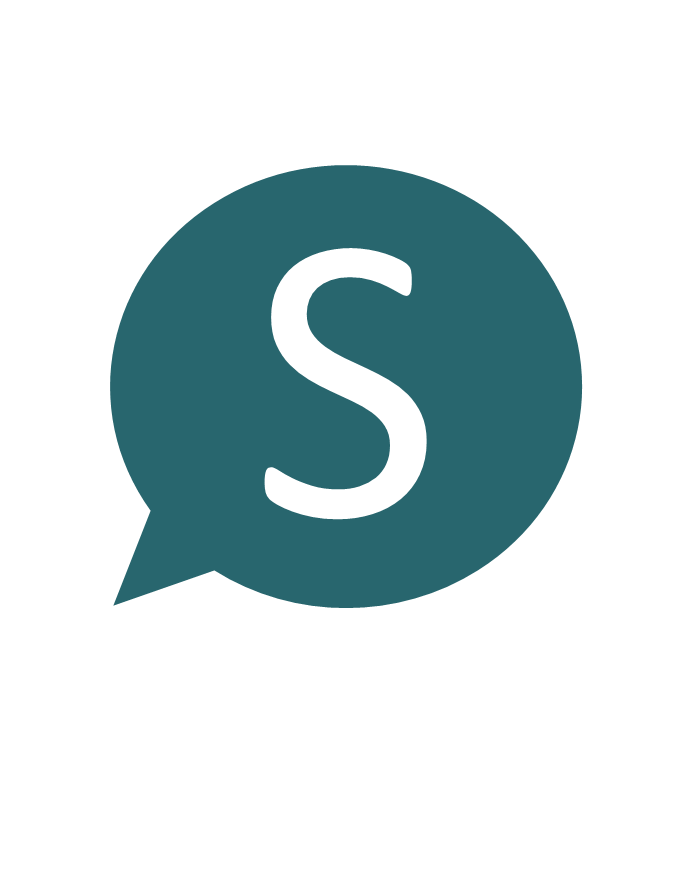 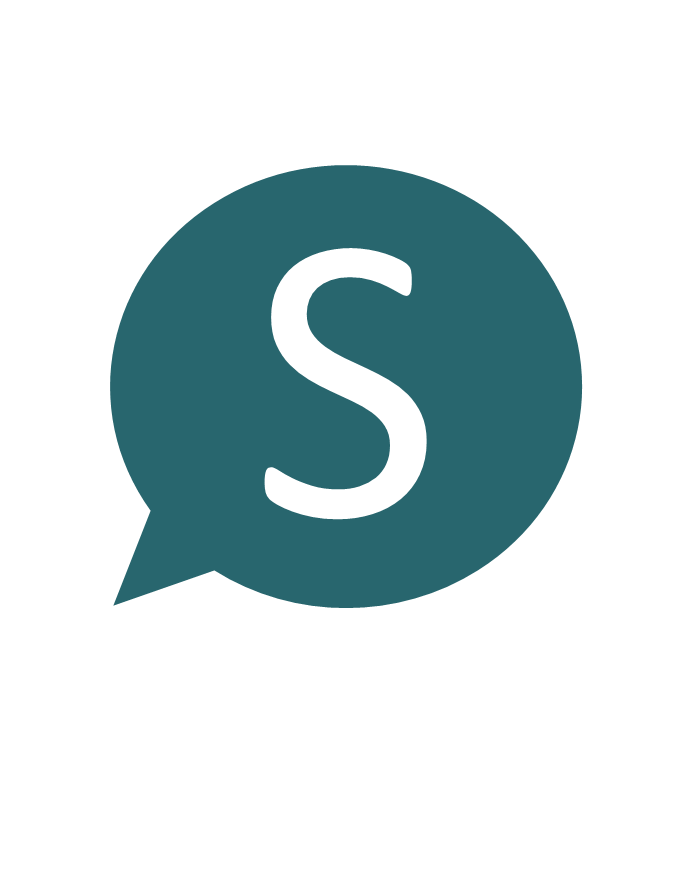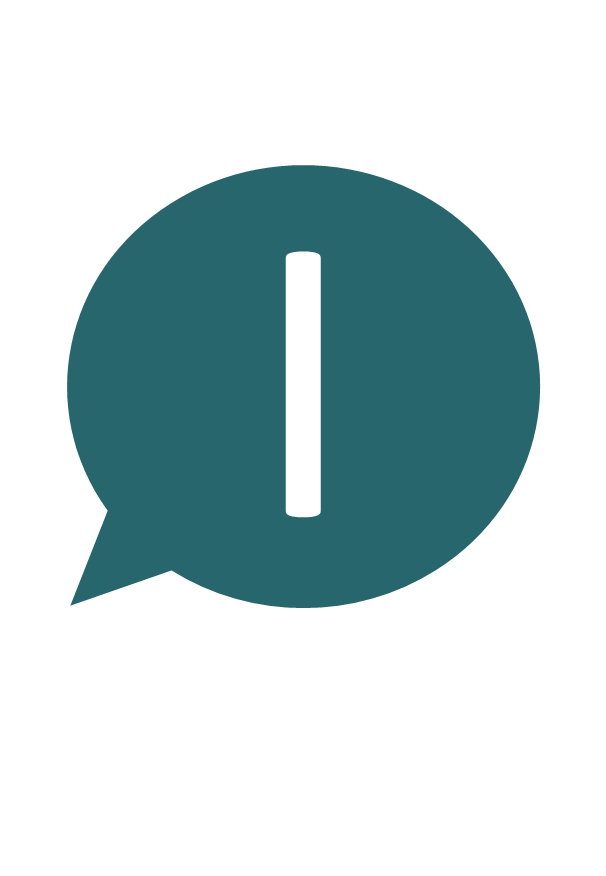 |  |  |  |  |  |  |  |  |  | 21 | 26 | 32 | 38 | 45 |
| Financial status, credit rating, knowledge, attitudes, behaviour | 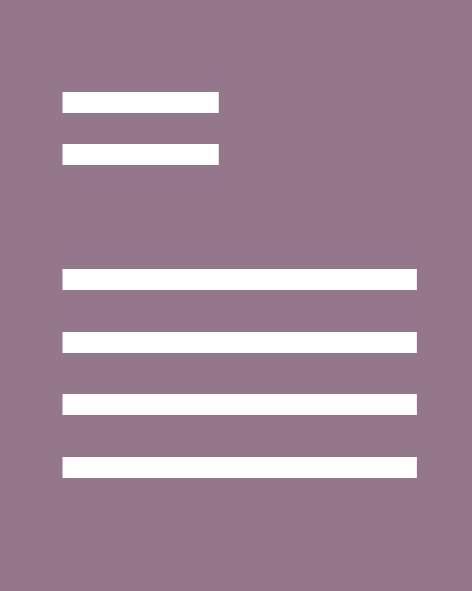 |  |  |  |  |  |  |  |  |  | 21 | 26 | 32 | 38 | 45 |
| Diet | 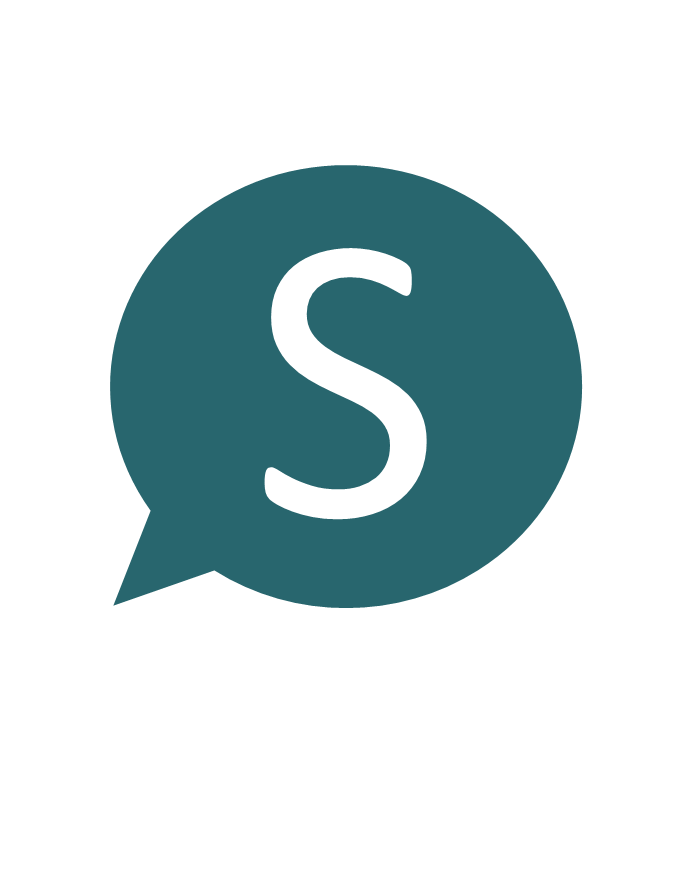 |  |  |  |  |  |  |  |  |  |  | 26 | 32 | 38 | 45 |
| Sleep | 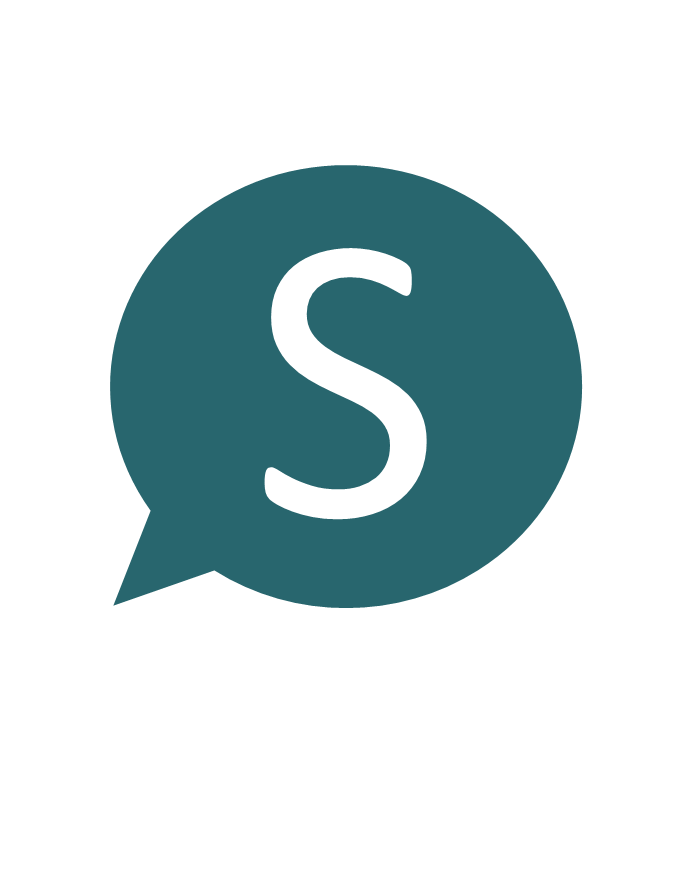 |  |  |  |  |  |  |  |  |  |  |  |  | 38 | 45 |
| New Zealand identity and society | 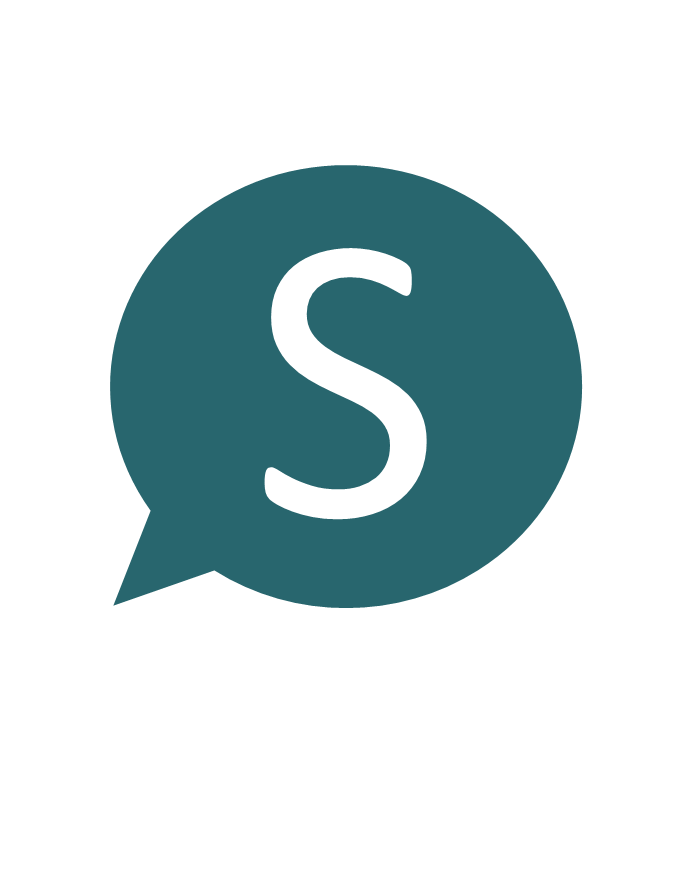 |  |  |  |  |  |  |  |  |  |  |  | 32 | 38 | 45 |
| **Physical health measures** |  |  |  |  |  |  |  |  |  |  |  |  |  |  |  |
| Foetal/newborn perinatal health | 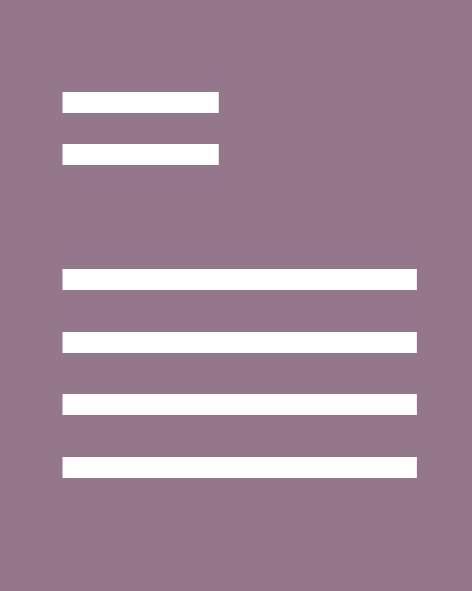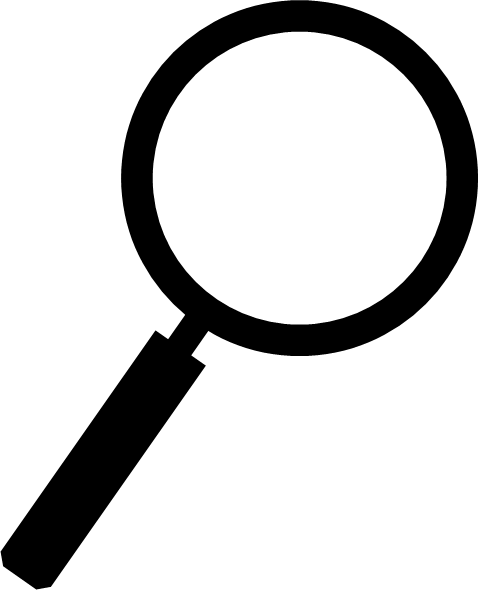 | 0 |  |  |  |  |  |  |  |  |  |  |  |  |  |
| Adiposity and anthropometrics | 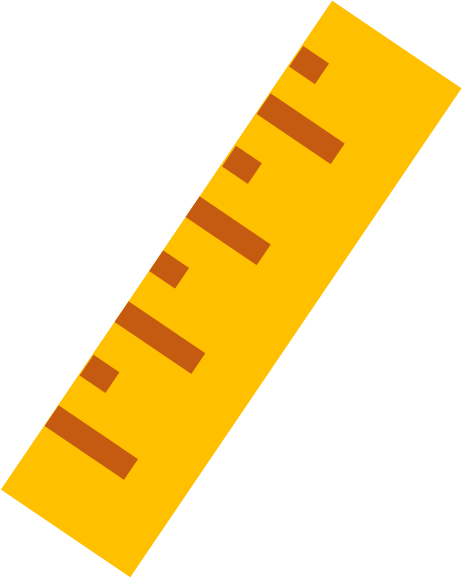 | 0 | 3 | 5 | 7 | 9 | 11 | 13 | 15 | 18 | 21 | 26 | 32 | 38 | 45 |
| Blood pressure, heart rate | 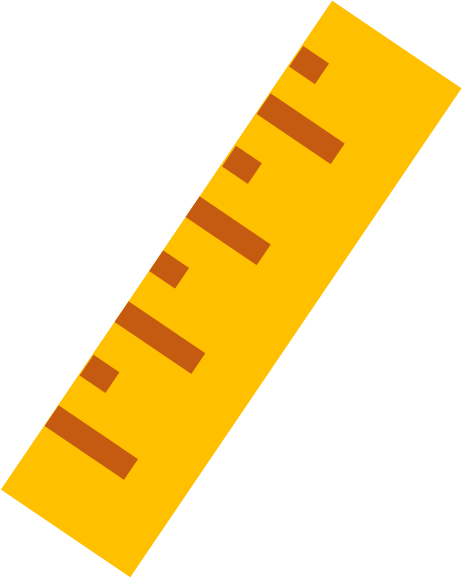 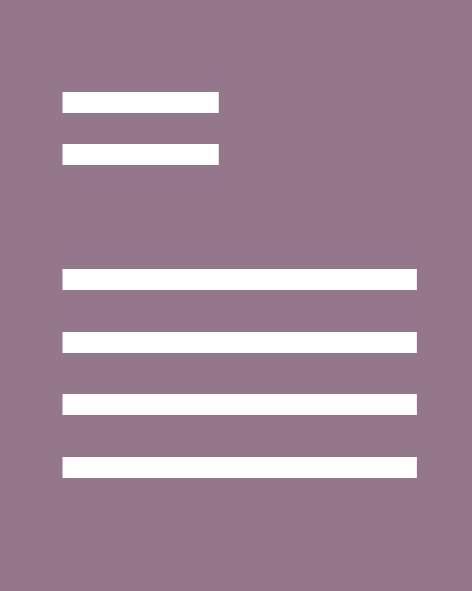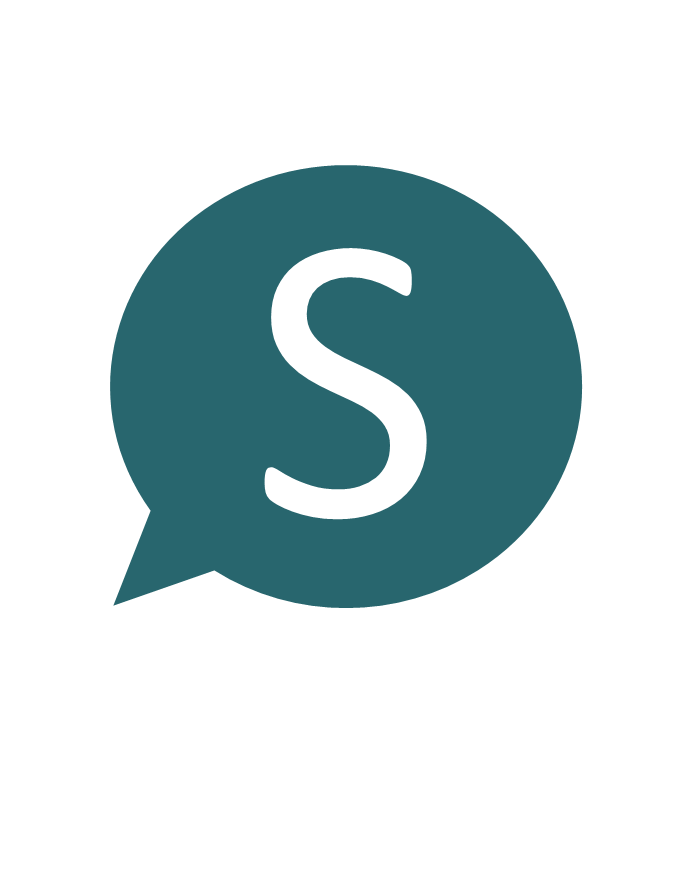 |  | 3 | 5 | 7 | 9 | 11 | 13 | 15 | 18 | 21 | 26 | 32 | 38 | 45 |
| Injuries | 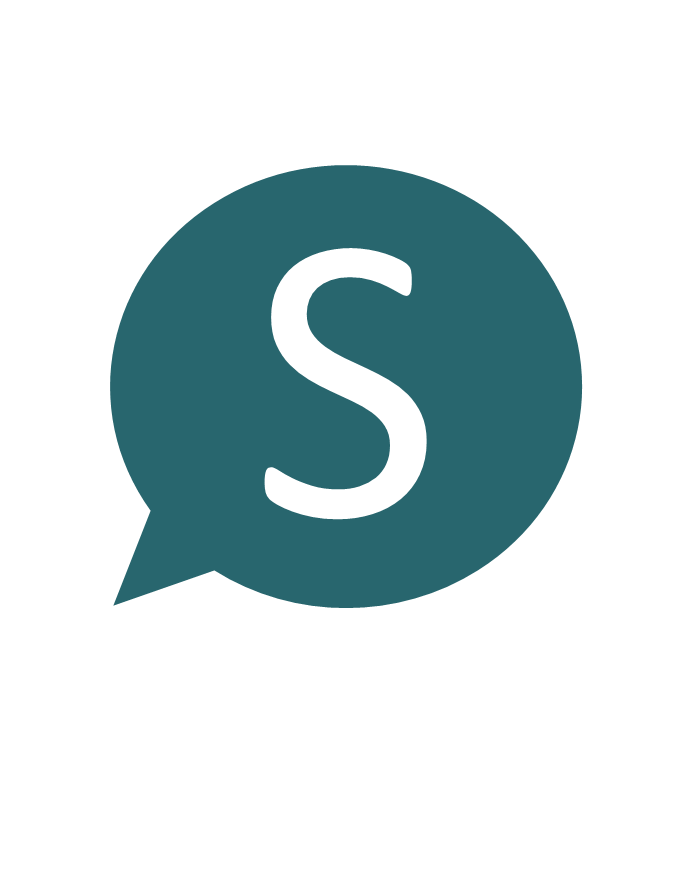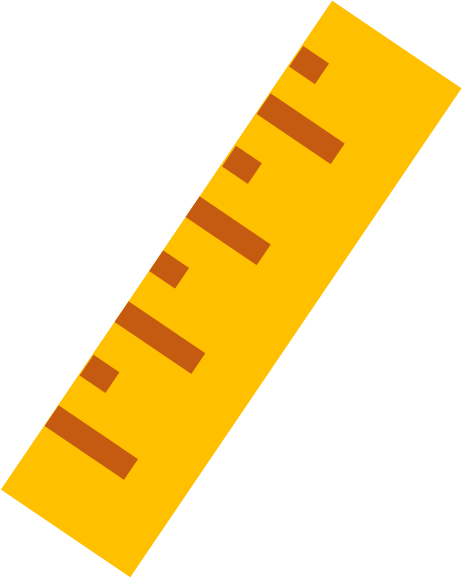 |  | 3 | 5 | 7 | 9 | 11 | 13 | 15 | 18 | 21 | 26 | 32 | 38 | 45 |
| Dental: periodontal disease, caries, alignment, attitudes | 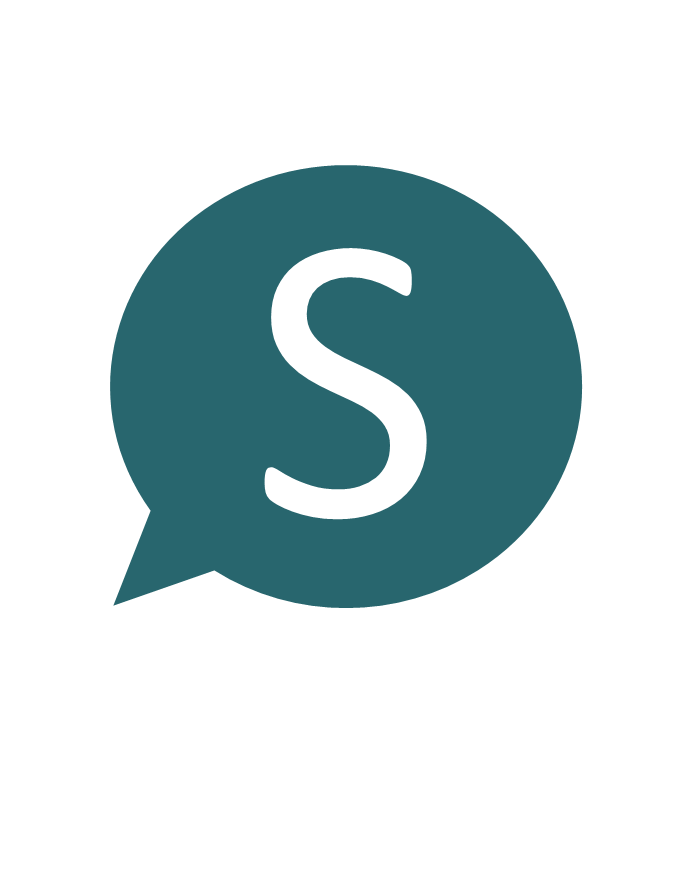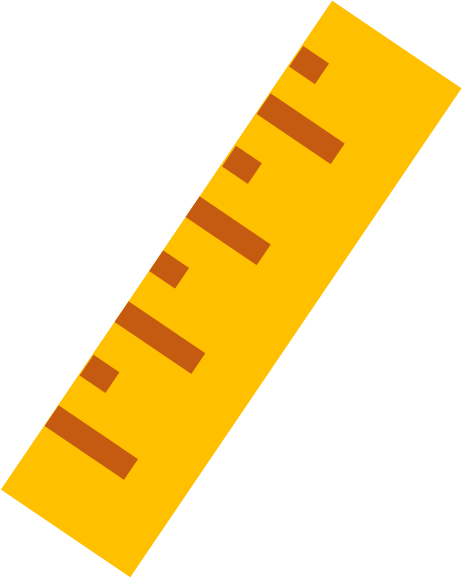 |  |  | 5 |  | 9 | 11 | 13 | 15 | 18 | 21 | 26 | 32 | 38 | 45 |
| Respiratory: asthma, allergy, and lung function tests |  |  |  |  |  | 9 | 11 | 13 | 15 | 18 | 21 | 26 | 32 | 38 | 45 |
| Aerobic fitness | 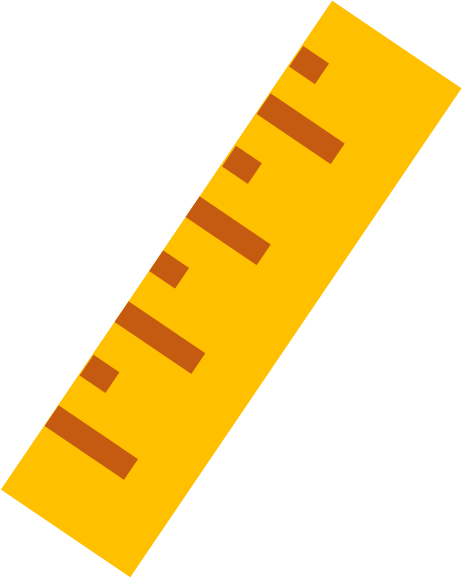 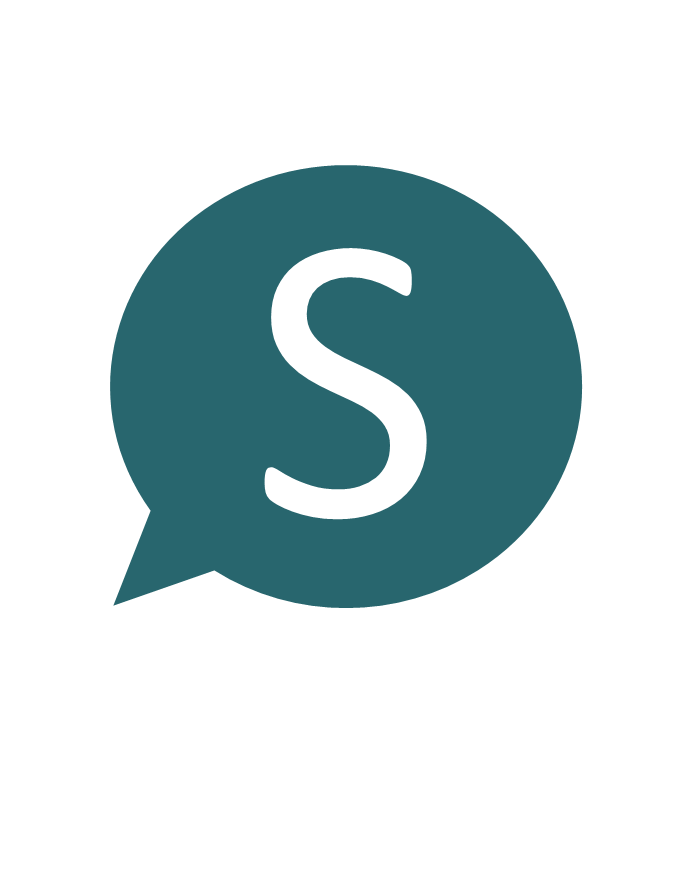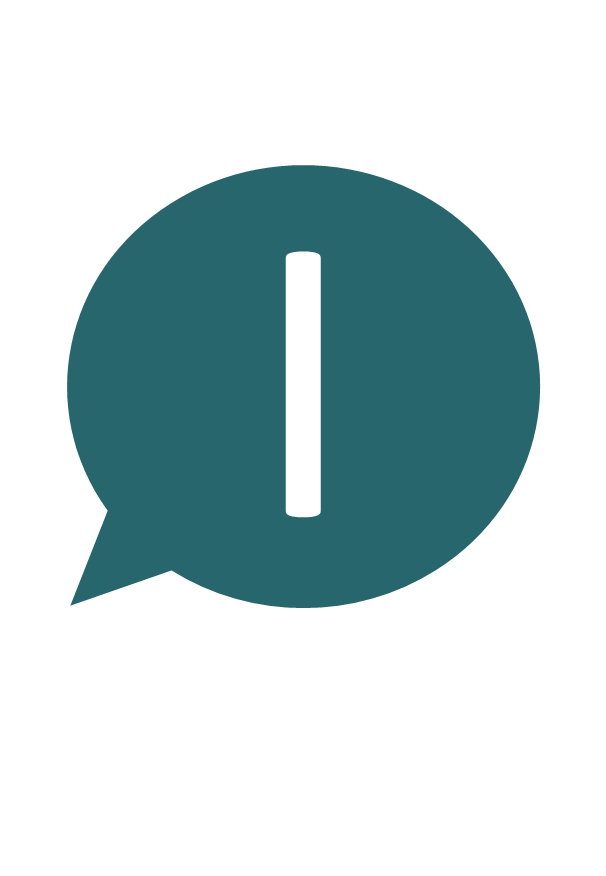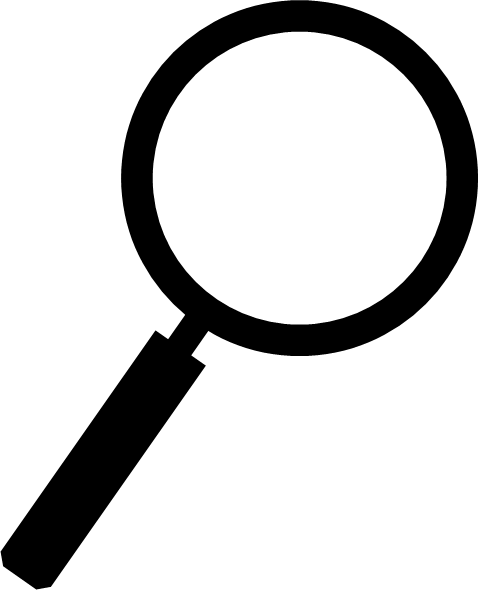 |  |  |  |  |  |  |  | 15 |  |  | 26 | 32 | 38 | 45 |
| Overall health status, health problems, diagnoses | 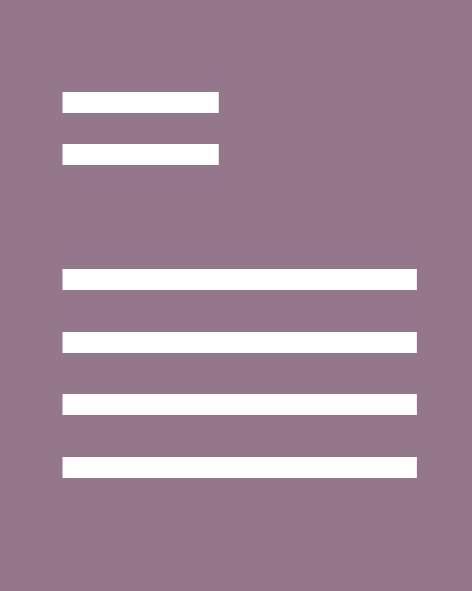 |  |  |  |  |  |  |  |  |  | 21 | 26 | 32 | 38 | 45 |
| Medications |  |  |  |  |  |  |  |  |  |  |  | 26 | 32 | 38 | 45 |
| Grip strength, balance | 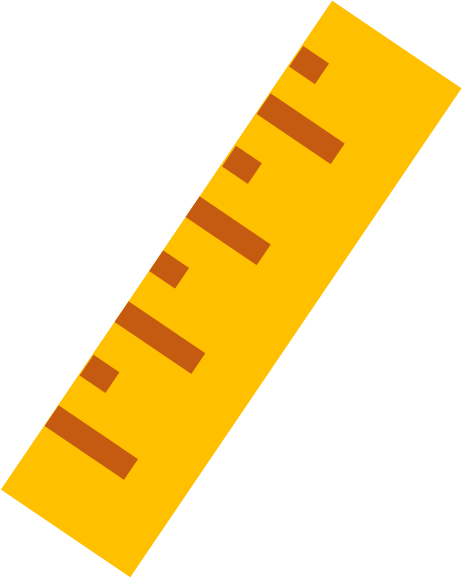 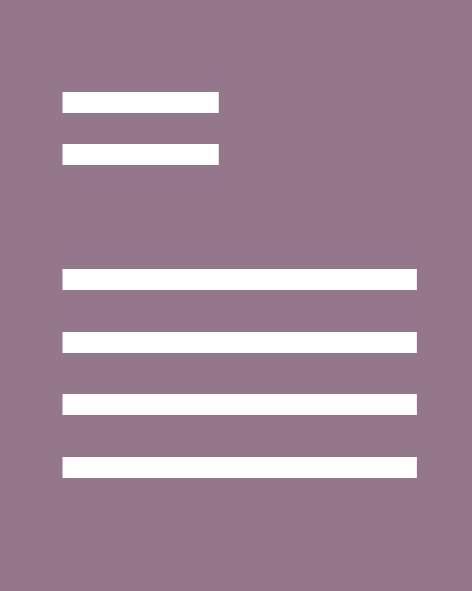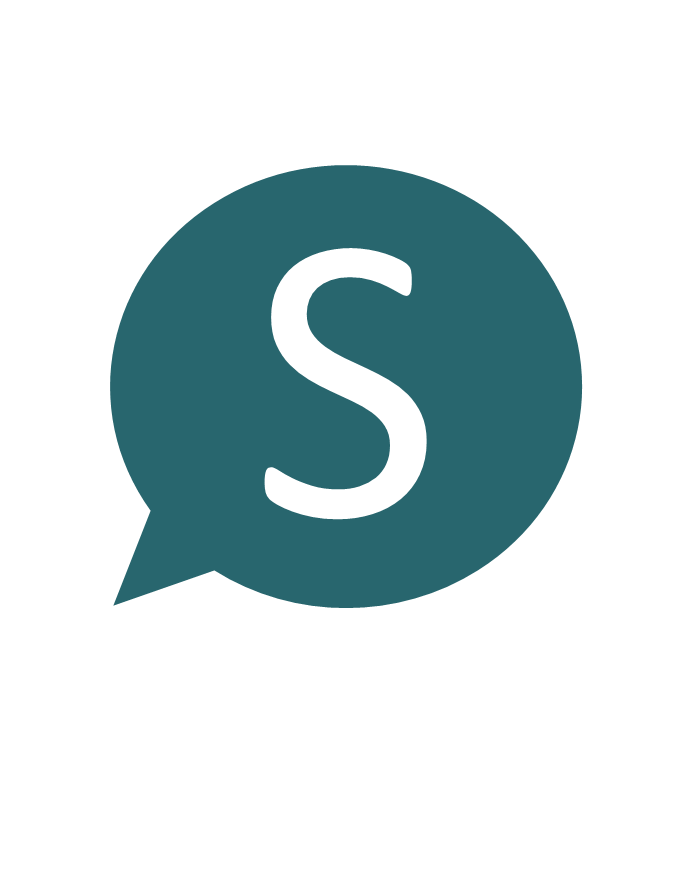 |  |  |  |  |  |  |  |  |  |  |  |  | 38 | 45 |
| Step-in-place, chair-stands, Gaitrite walking tests | 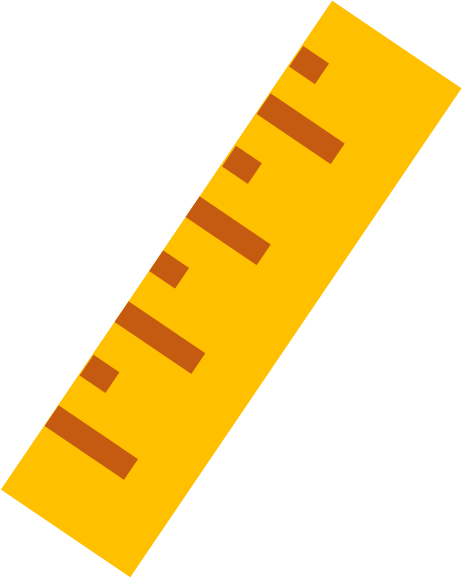 |  |  |  |  |  |  |  |  |  |  |  |  |  | 45 |
| Endothelial function | 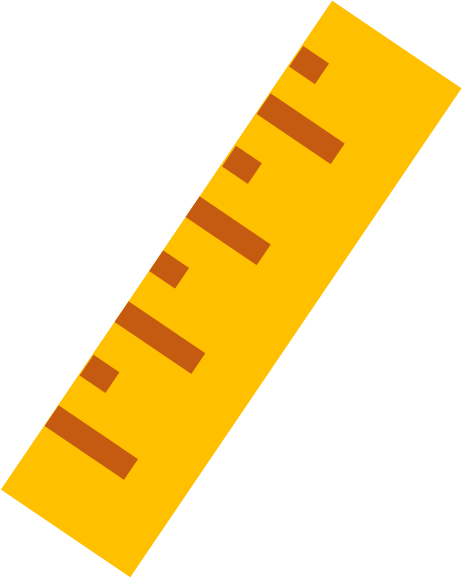 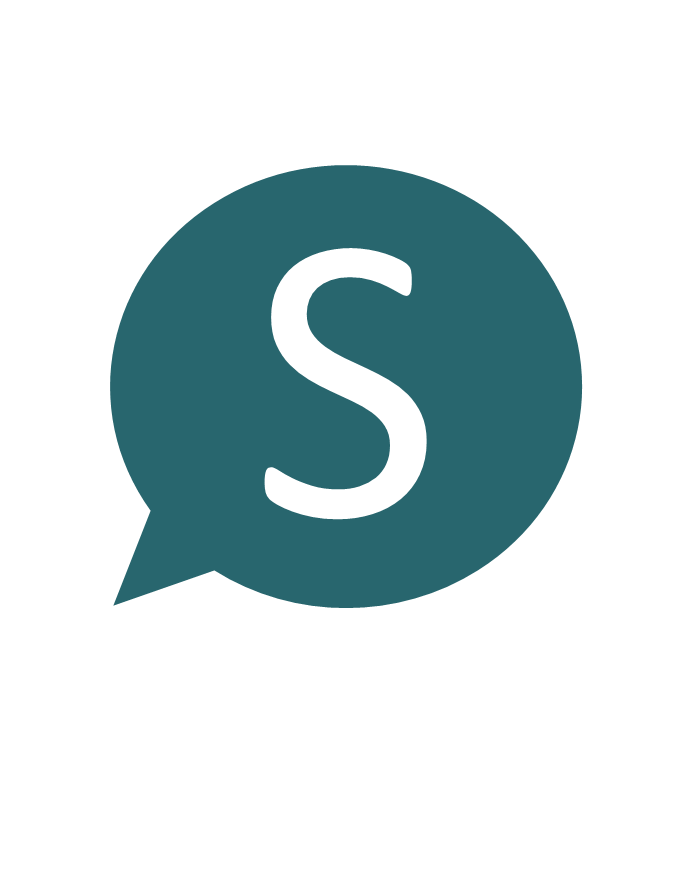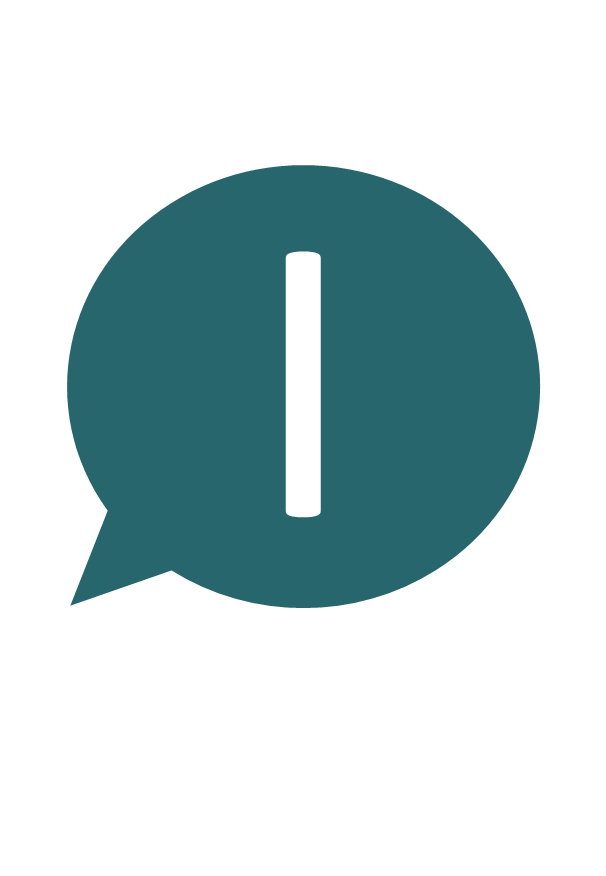 |  |  |  |  |  |  |  |  |  |  |  |  | 38 |  |
| Facial photography, ratings of facial aging | 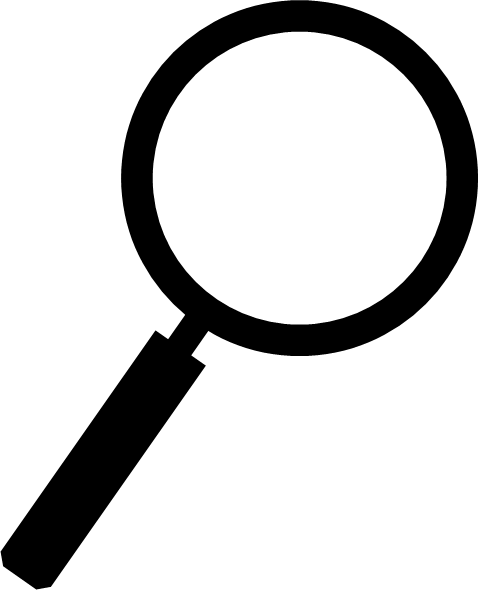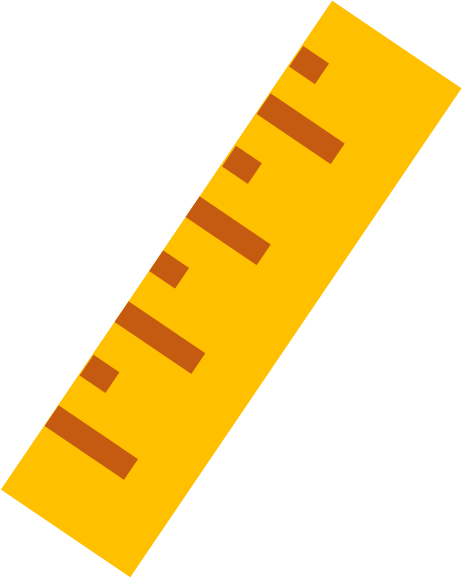 |  |  |  |  |  |  |  |  |  |  |  |  | 38 | 45 |
| Digital imaging of retinal micro-vasculature | 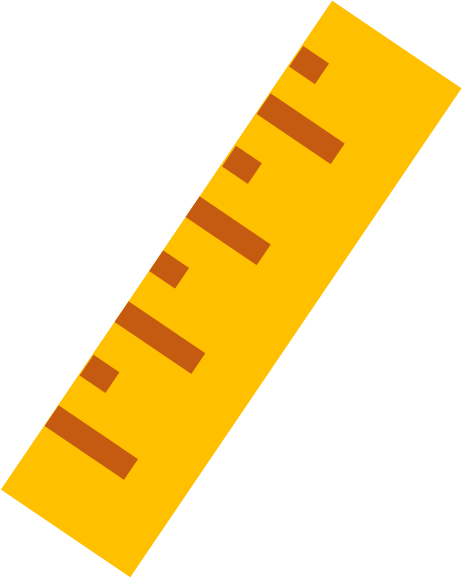 |  |  |  |  |  |  |  |  |  |  |  |  | 38 | 45 |
| Optical coherence tomography (OCT): optical nerve scan | 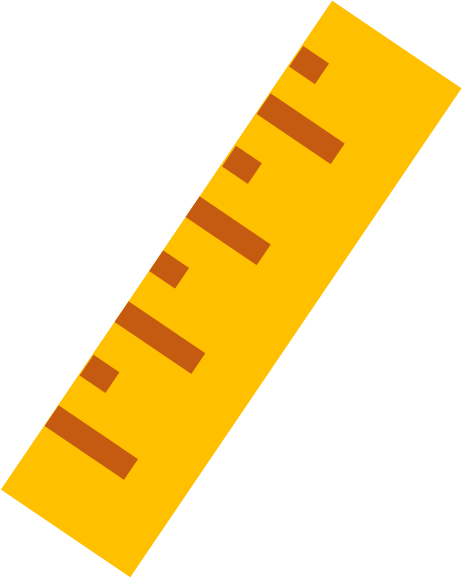 |  |  |  |  |  |  |  |  |  |  |  |  |  | 45 |
| Dry eye: eyelid glands, eye lipid layer | 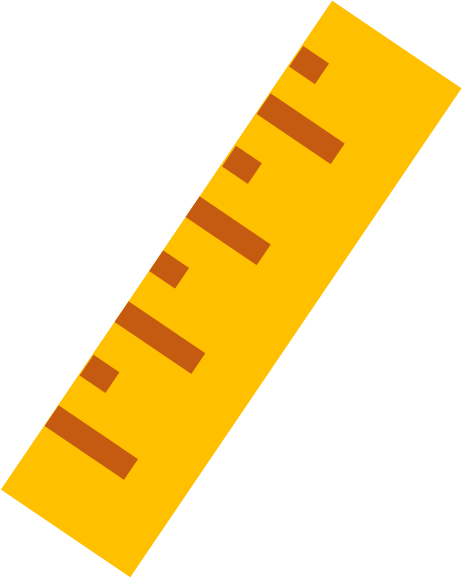 |  |  |  |  |  |  |  |  |  |  |  |  |  | 45 |
| Vision: peripheral visual field testing, acuity, contrast perception | 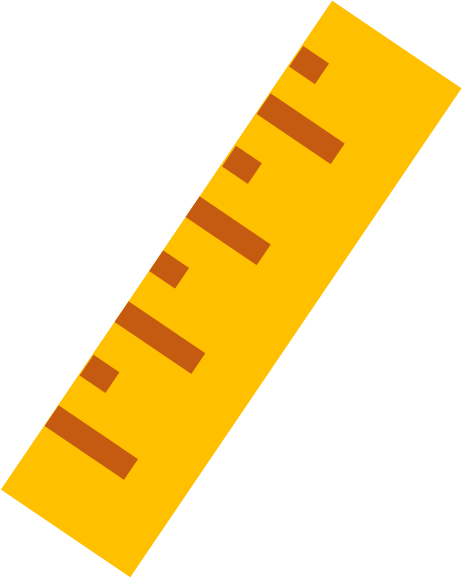 |  |  |  |  |  |  |  |  |  |  |  |  |  | 45 |
| Hearing: speech-in-noise test, hearing tests | 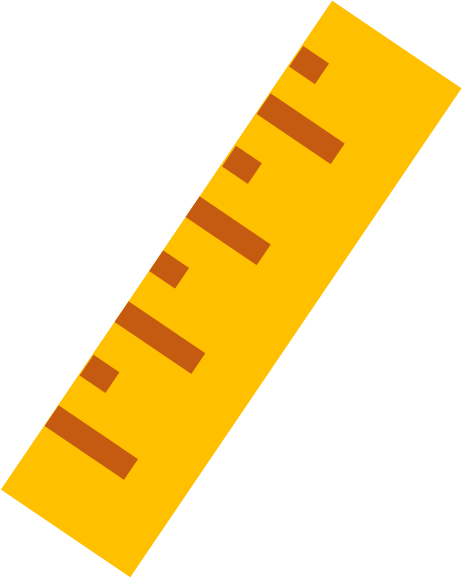 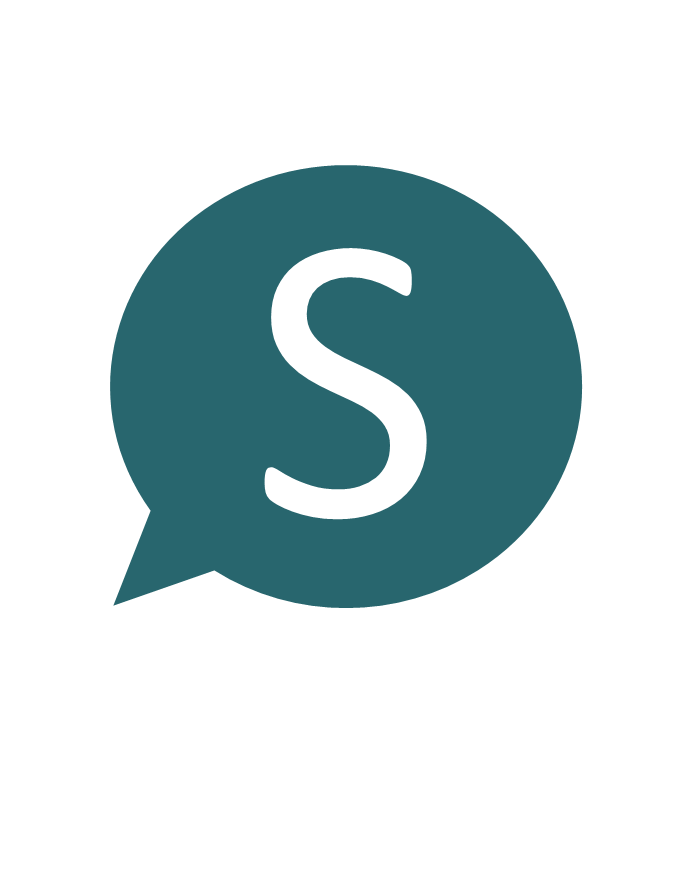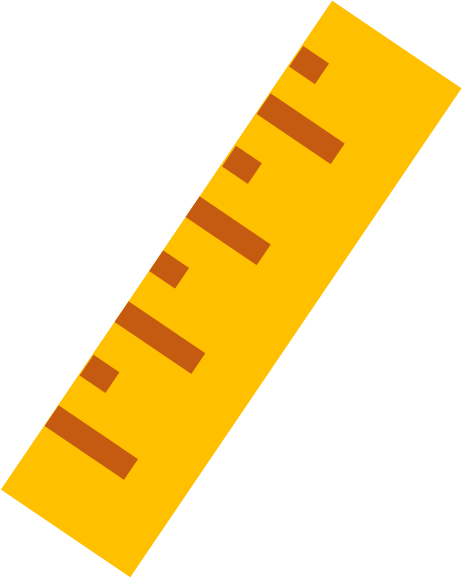 |  |  |  |  |  |  |  |  |  |  |  |  |  | 45 |
| Pain: sensitivity, thresholds, experiences |  |  |  |  |  |  |  |  |  |  |  |  |  |  | 45 |
| Dual-energy X-ray absorptiometry (DXA) scan: bone density | 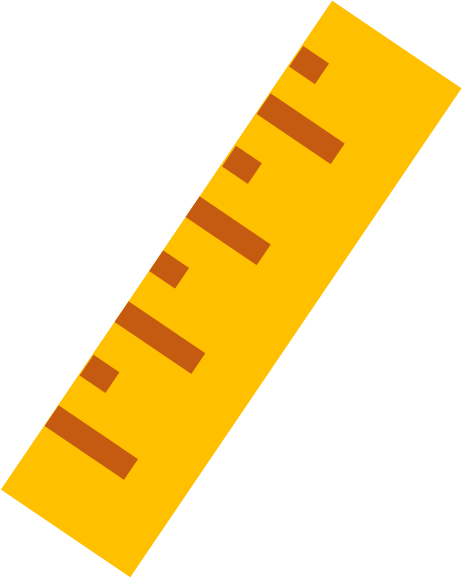 |  |  |  |  |  |  |  |  |  |  |  |  |  | 45 |
| **Biorepository** |  |  |  |  |  |  |  |  |  |  |  |  |  |  |  |
| DNA/RNA bank (from blood) | 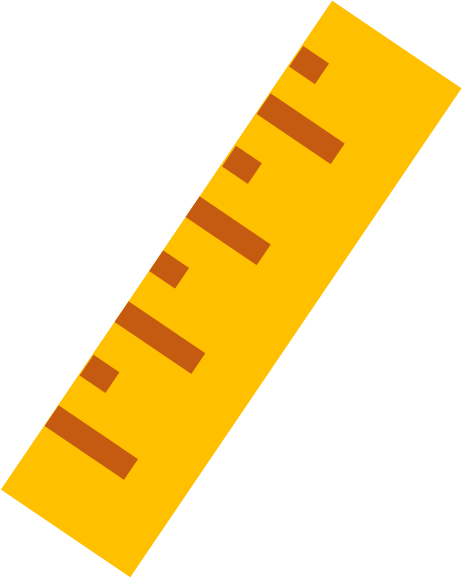 |  |  |  |  |  |  |  |  |  |  | 26 | 32 | 38 | 45 |
| Blood biomarkers (e.g., cholesterol, glycated haemoglobin, c-reactive protein)^1^ | 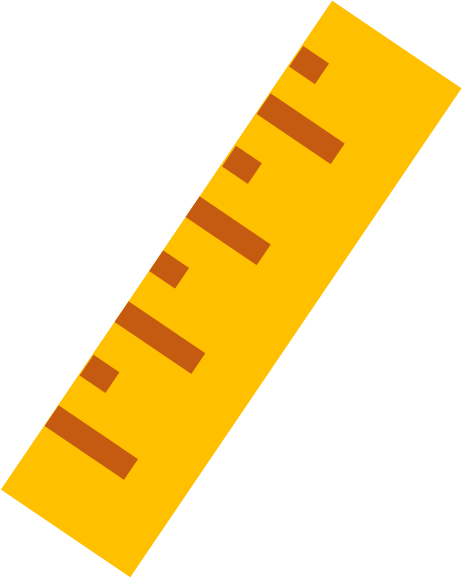 |  |  |  |  |  |  |  |  |  |  | 26 | 32 | 38 | 45 |
| Genome-wide single nucleotide polymorphism scan | 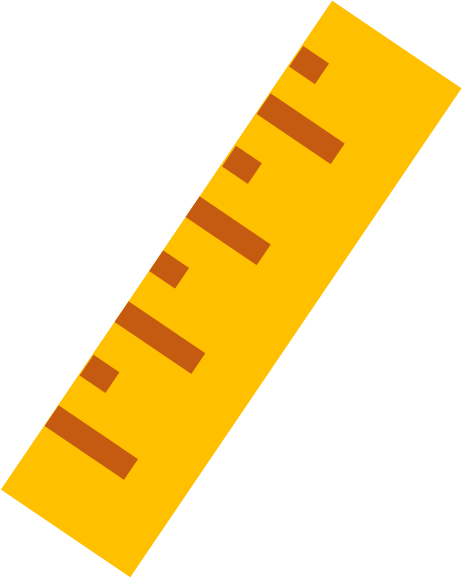 |  |  |  |  |  |  |  |  |  |  |  |  | 38 |  |
| Urinary biomarkers | 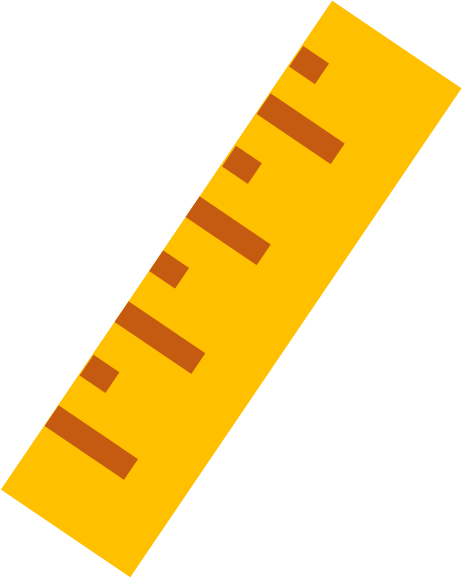 |  |  |  |  |  |  |  |  |  |  |  |  |  | 45 |
| **Brain health** | 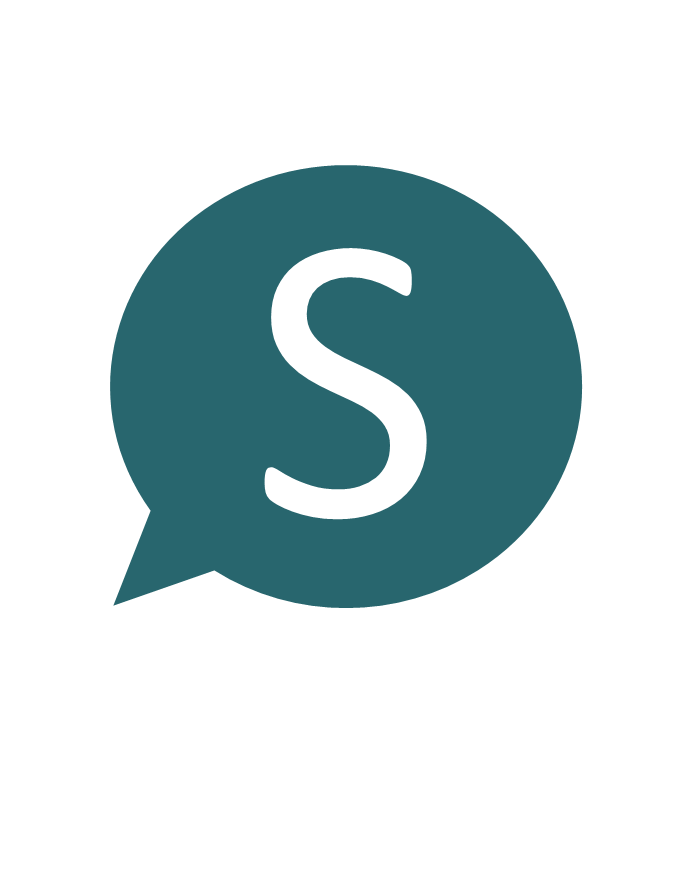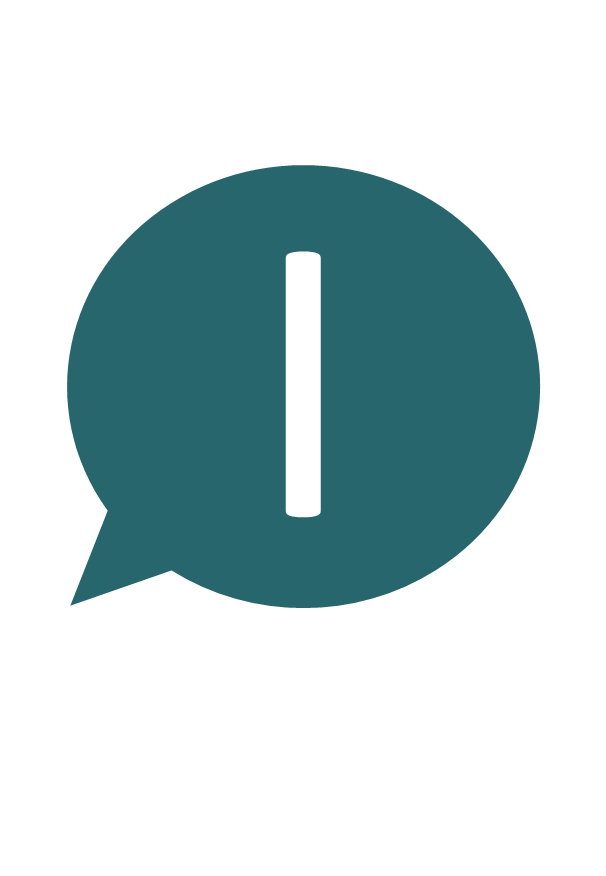 |  |  |  |  |  |  |  |  |  |  |  |  |  |  |
| Perceived cognitive health |  |  |  |  |  |  |  |  |  |  |  |  |  |  | 45 |
| ***Magnetic Resonance Imaging (structural indices)*** |  |  |  |  |  |  |  |  |  |  |  |  |  |  |  |
| Grey matter: cortical thickness, cortical surface area, hippocampal volume | 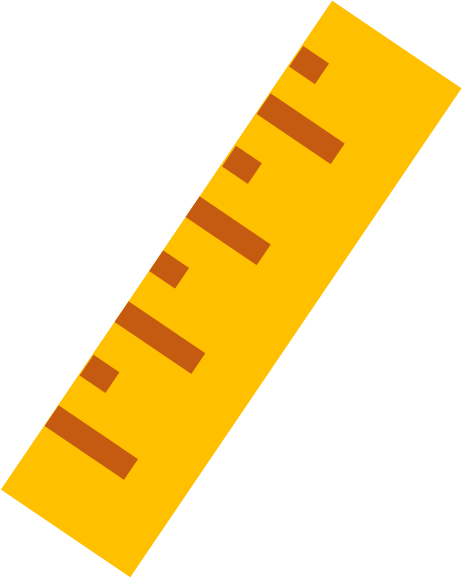 |  |  |  |  |  |  |  |  |  |  |  |  |  | 45 |
| White matter: hyperintensities, fractional anisotropy | 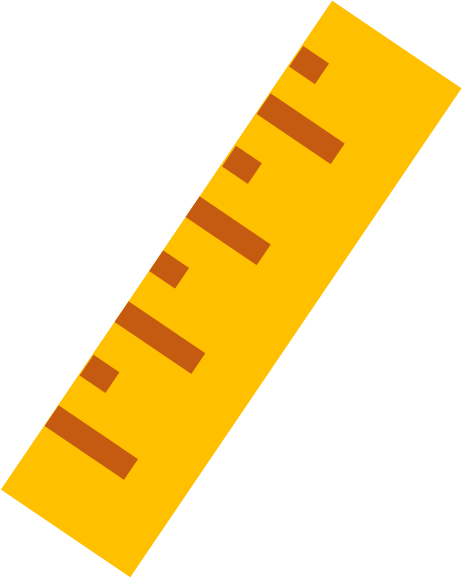 |  |  |  |  |  |  |  |  |  |  |  |  |  | 45 |
| BrainAGE | 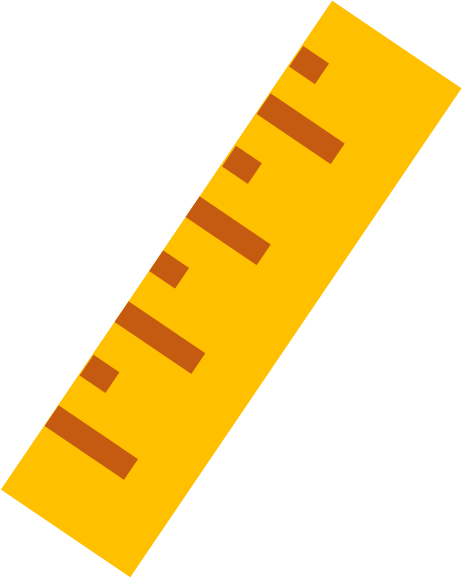 |  |  |  |  |  |  |  |  |  |  |  |  |  | 45 |
| ***Functional Magnetic Resonance Imaging (functional indices)*** |  |  |  |  |  |  |  |  |  |  |  |  |  |  |  |
| Face-matching emotion processing task (amygdala function) | 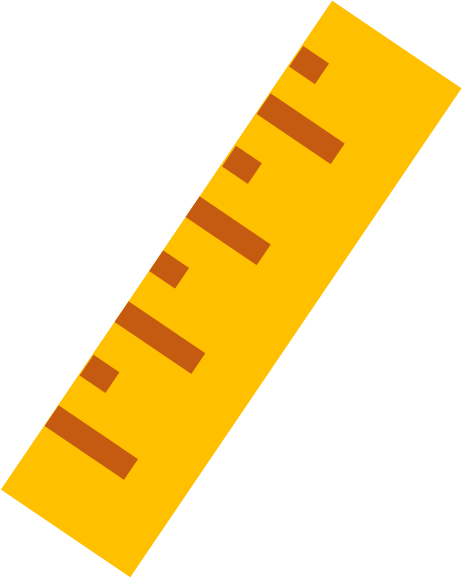 |  |  |  |  |  |  |  |  |  |  |  |  |  | 45 |
| Stroop executive-function task (dlPFC and dorsal anterior cingulate cortex function) | 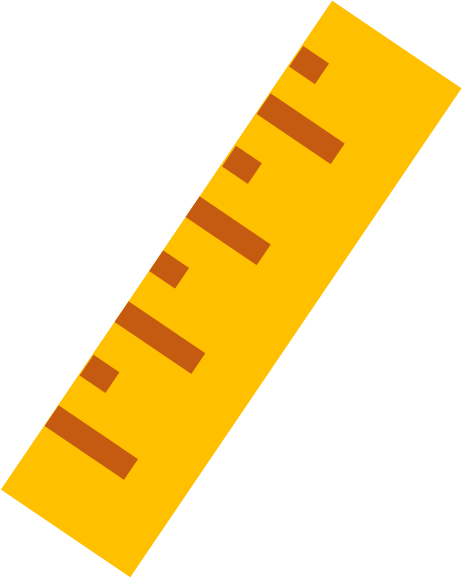 |  |  |  |  |  |  |  |  |  |  |  |  |  | 45 |
| Monetary-incentive delay-rewards task (ventral striatum function) | 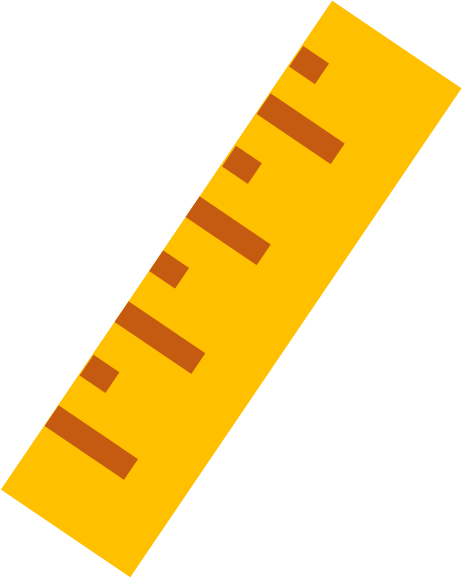 |  |  |  |  |  |  |  |  |  |  |  |  |  | 45 |
| Face-name-encoding episodic memory task (hippocampus function) | 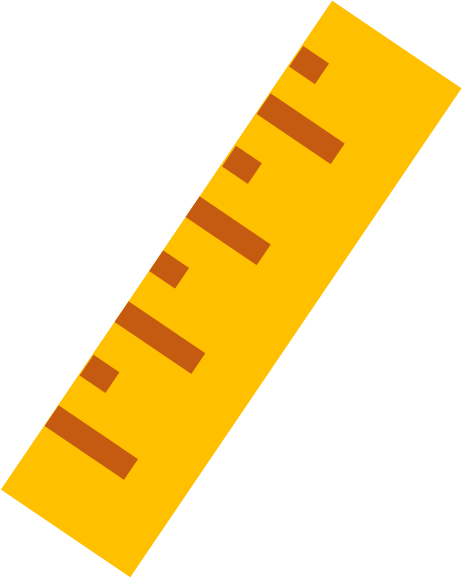 |  |  |  |  |  |  |  |  |  |  |  |  |  | 45 |


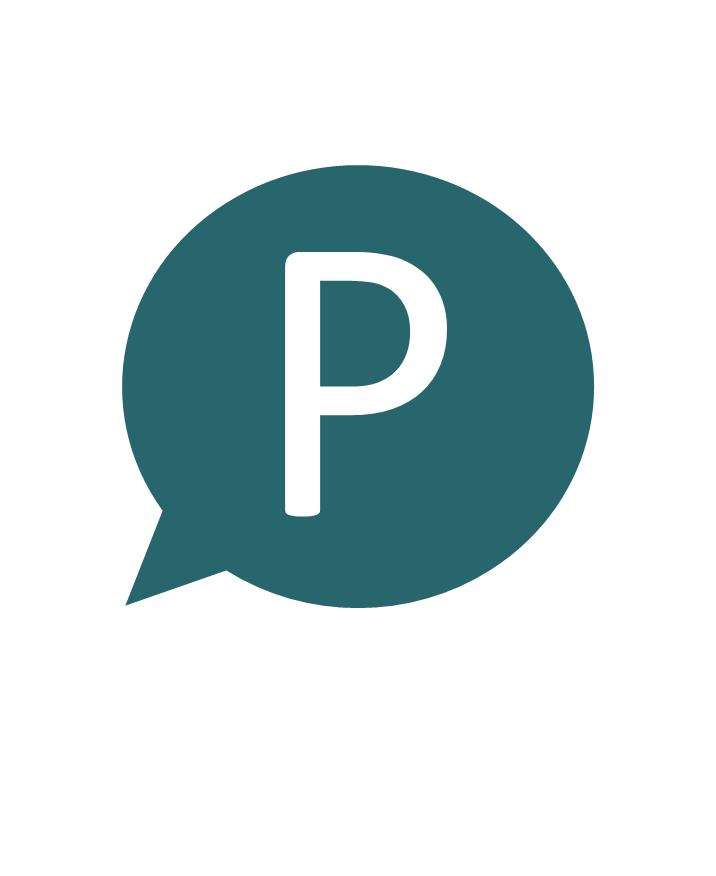
*Notes.*

**
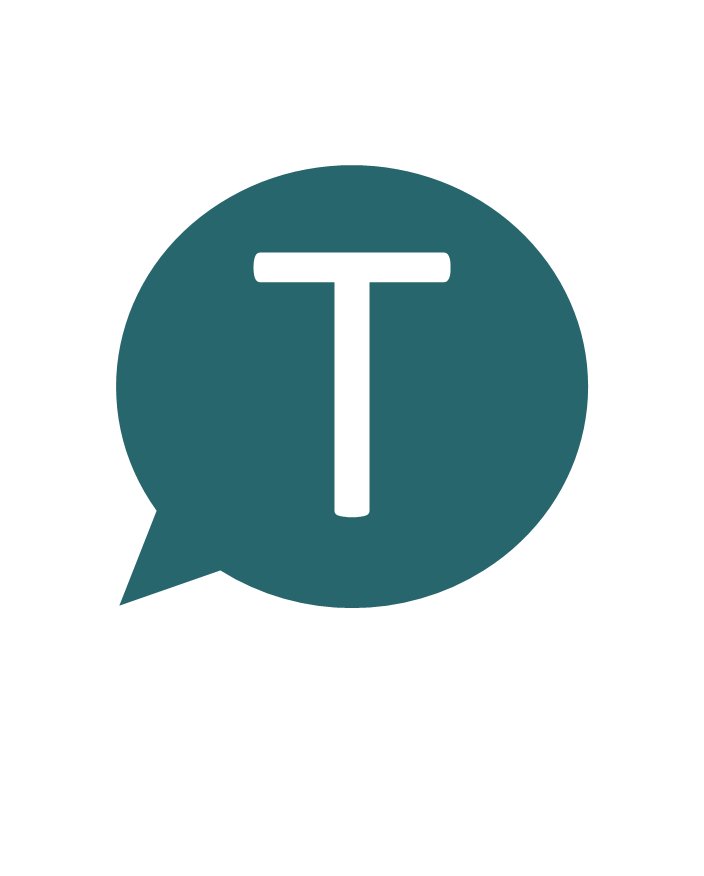
** = parent-reported


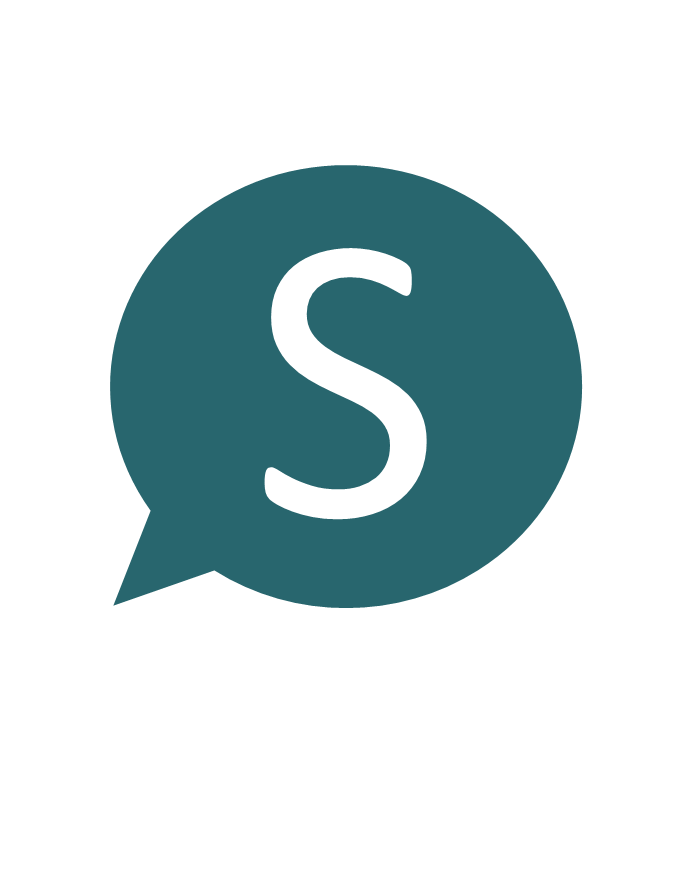
 = teacher-reported


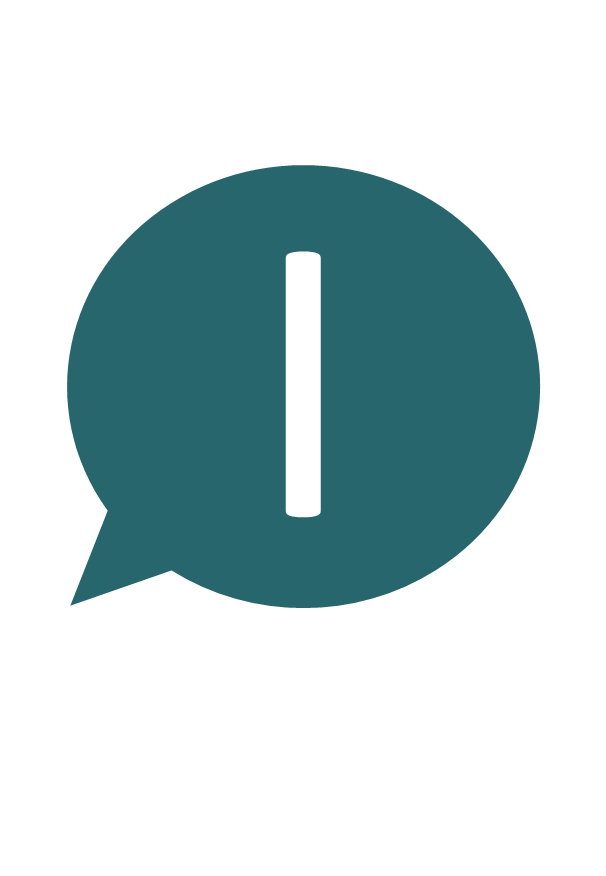
 = self-reported

= informant-reported (close friend or family member)


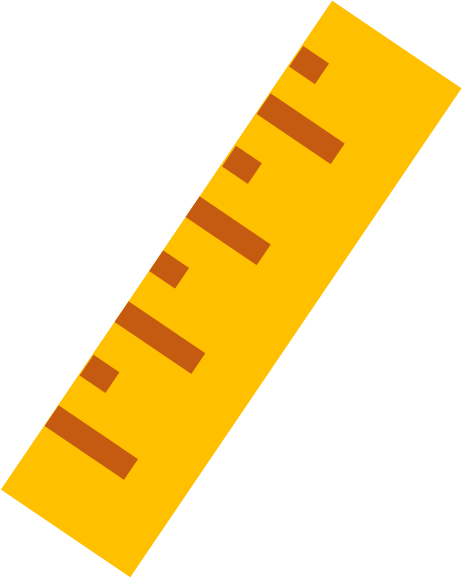

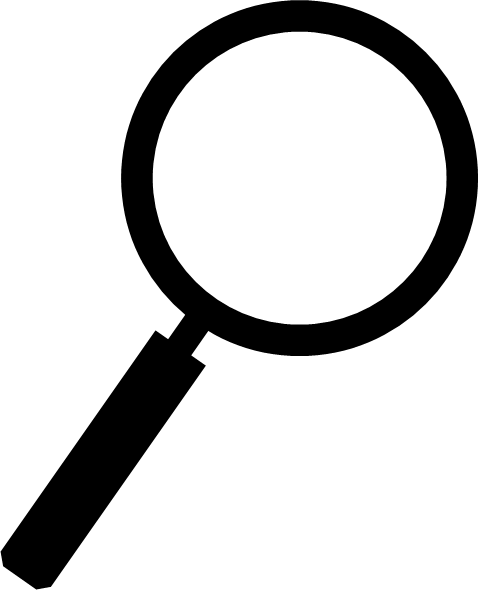
 = observations by hospital or research staff

**
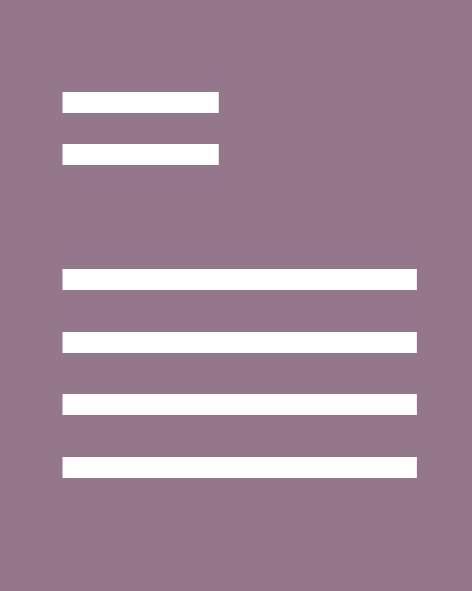
** = objective quantitative test

= hospital or administrative record

dlPFC = dorsolateral prefrontal cortex. ^1^Many blood biomarkers were assessed repeatedly from age 26 to 45, but not all biomarkers were assessed at every phase. For clarity, Table S1b shows the specific blood biomarkers assessed at each phase.

**Table S1b. Blood biomarkers measured in the Dunedin Study, ages 26 to 45y**

*Notes.* ^1^Biomarkers were typically measured from age 26 onwards, but at age 11 a subset of Study members provided blood samples for research into the effects of blood lead levels (e.g., Reuben et al., 2017, 2019, 2020; Beckley et al., 2018).

^2^For example, packed cell volume (haematocrit), mean corpuscular volume, red blood cell counts at specific phases.

^3^Specific cell types and total cell counts.

|  | Age | | | |
| --- | --- | --- | --- | --- |
| Blood biomarker^1^ | 26 | 32 | 38 | 45 |
| Adiponectin |  | 32 | 38 | 45 |
| A1 Antitrypsin |  | 32 |  | 45 |
| Alanine aminotransferase (ALT) |  |  | 38 | 45 |
| Albumin |  |  | 38 | 45 |
| Alkaline phosphatase |  |  | 38 | 45 |
| Alpha-amylase |  |  |  | 45 |
| Androsterone sulfate (AoS) |  |  |  | 45 |
| Antinuclear antibodies |  |  |  | 45 |
| Apolipoprotein A1 | 26 | 32 | 38 | 45 |
| Apolipoprotein B | 26 | 32 | 38 | 45 |
| Aspartate aminotransferase (AST) |  |  | 38 | 45 |
| Asymmetric dimethylarginine (ADMA) |  |  |  | 45 |
| Beta-2-microglobulin |  |  |  | 45 |
| Bilirubin |  |  | 38 | 45 |
| Calcium |  |  |  | 45 |
| Chloride |  |  |  | 45 |
| Cortisol | 26 | 32 | 38 | 45 |
| C-reactive protein | 26 | 32 | 38 | 45 |
| Creatinine | 26 | 32 | 38 | 45 |
| Cystatin C |  | 32 | 38 | 45 |
| Cytomegalovirus antibodies |  |  | 38 | 45 |
| D-dimer |  |  |  | 45 |
| Dehydroepiandrosterone sulfate (DHEAS)  [Dehydroepiandrosterone sulfate (DHEAS)](https://labtestsonline.org/tests/dheas) |  | 32 | 38 | 45 |
| Estradiol | 26 |  | 38 | 45 |
| Ferritin | 26 |  |  | 45 |
| Fibrinogen |  | 32 | 38 | 45 |
| Gamma glutamyltransferase (GGT) |  |  | 38 | 45 |
| Globulin |  |  | 38 | 45 |
| Glycated haemoglobin | 26 | 32 | 38 | 45 |
| Growth differentiation factor-15 |  |  |  | 45 |
| Haemoglobin | 26 | 32 | 38 | 45 |
| Herpes simplex virus Type 1 and 2 | 26 | 32 | 38 | 45 |
| High density lipoprotein (HDL) cholesterol | 26 | 32 | 38 | 45 |
| Immunoglobulin A (IgA) |  |  |  | 45 |
| Immunoglobulin E (IgE) |  | 32 |  | 45 |
| Immunoglobulin G (IgG) |  |  |  | 45 |
| Immunoglobulin M (IgM) |  |  |  | 45 |
| Insulin |  |  | 38 | 45 |
| Insulin-like growth factor 1 |  |  |  | 45 |
| Interleukin-6 (IL6) |  |  | 38 | 45 |
| Iron | 26 |  |  | 45 |
| Lactate dehydrogenase |  |  |  | 45 |
| L-arginine |  |  |  | 45 |
| L-citrulline |  |  |  | 45 |
| Leptin |  | 32 | 38 | 45 |
| Lipoprotein A | 26 | 32 | 38 | 45 |
| Low density lipoprotein (LDL) cholesterol | 26 | 32 | 38 | 45 |
| Platelets | 26 | 32 | 38 | 45 |
| Potassium |  |  |  | 45 |
| Red blood cells (various)^2^ | 26 | 32 | 38 | 45 |
| Sex-hormone binding globulin | 26 |  | 38 | 45 |
| Sodium |  |  |  | 45 |
| Symmetric dimethylarginine (SDMA) |  |  |  | 45 |
| Testosterone | 26 |  | 38 | 45 |
| Total cholesterol | 26 | 32 | 38 | 45 |
| Total protein |  |  | 38 | 45 |
| Toxoplasma IgG, IgM |  |  | 38 | 45 |
| Transferrin | 26 |  |  | 45 |
| Triglycerides | 26 | 32 | 38 | 45 |
| Tumour necrosis factor |  |  |  | 45 |
| Urea | 26 | 32 | 38 | 45 |
| Urea nitrogen (BUN) |  |  |  | 45 |
| Vitamin D |  |  |  | 45 |
| Uric acid |  |  |  | 45 |
| White blood cells (various)^3^ | 26 | 32 | 38 | 45 |

**2. Dunedin Study member retention and follow-up**

A key success of the Dunedin Study its high retention rate, which is in part maintained by keeping in contact with Study members no matter where in the world they live. Table S2 shows the retention rate across the assessment phases to date, and Figure S1 shows where in the world Study members were living at the time of phase 45.

**Table S2. Participant retention in the Dunedin Study, birth to age 45y**

| Age | Year | Number seen | Percent^1^ |
| --- | --- | --- | --- |
| Birth | 1972-73 |  |  |
| 3 | 1975-76 | 1037 | 100% |
| 5 | 1977-78 | 991 | 96% |
| 7 | 1979-80 | 954 | 92% |
| 9 | 1981-82 | 955 | 92% |
| 11 | 1983-84 | 925 | 90% |
| 13 | 1985-86 | 850 | 82% |
| 15 | 1987-88 | 976 | 95% |
| 18 | 1990-91 | 993 | 97% |
| 21 | 1993-94 | 992 | 97% |
| 26 | 1998-99 | 980 | 96% |
| 32 | 2004-05 | 972 | 96% |
| 38 | 2010-12 | 961 | 95% |
| 45 | 2017-19 | 938 | 94% |

^1^Percentage of Study members living at each age

**
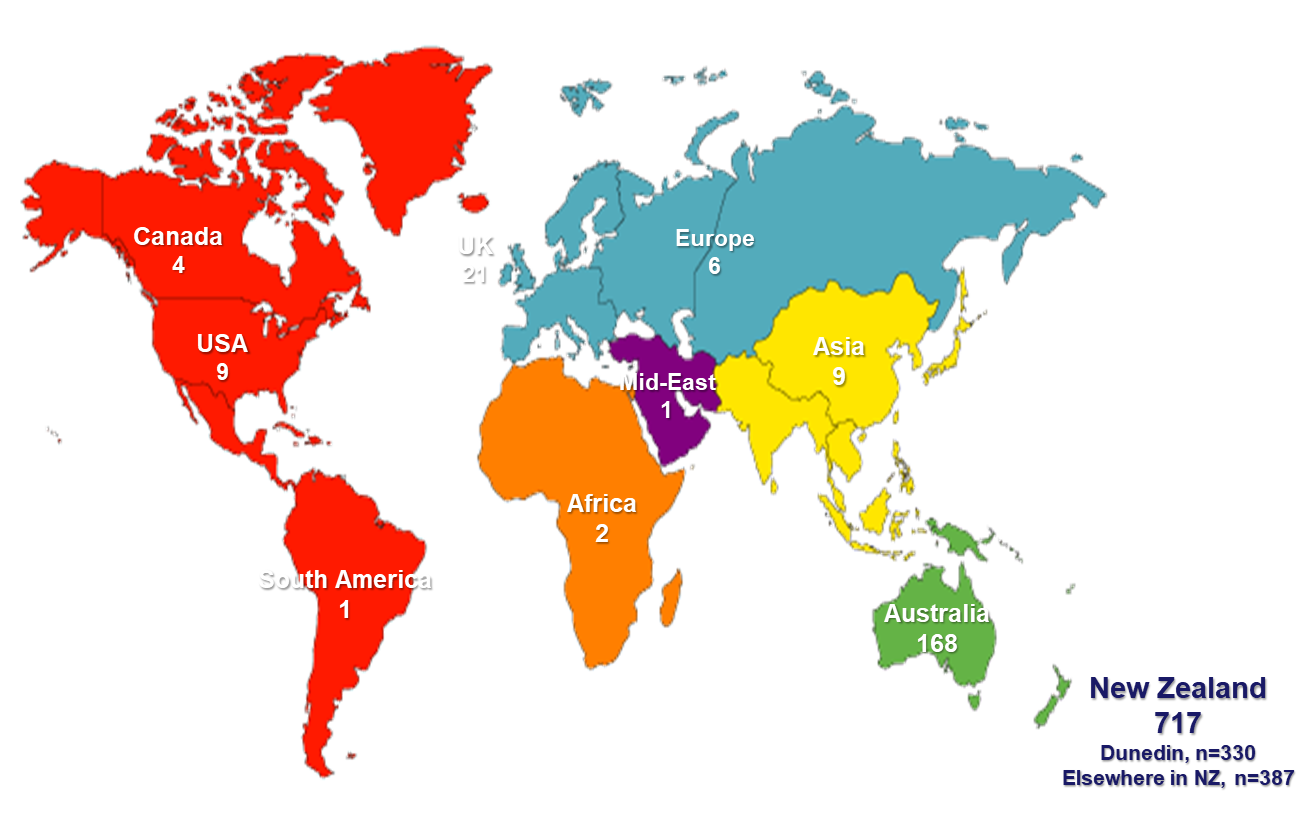
**

*Figure S1.* Study members’ country of residence at the time of the phase 45 assessment

**3. Sub-studies that have evolved out of the Dunedin Study**

The commitment of Dunedin Study members and their families over the years has enabled study investigators to look beyond the bounds of the Dunedin Study itself and to develop several sub-studies that explore intergenerational patterns in health and development with Study members’ parents, grandparents, and children, or to consider topical issues that emerge outside of the normal Dunedin Study assessment cycle. Here we summarise these sub-studies, which include the Family Health History Study, Parenting Study, Next Generation Study, Dementia Study, and COVID-19 Vaccination Study.

***3.1 Family Health History Study***

The Family Health History Study was designed to enable comprehensive investigations of the links between family history and health, which could be used to better understand individual differences in disease risk and severity, treatment efficacy, and prognosis. The Dunedin Study provided the ideal opportunity to investigate family history and health links given its general population sample, rich biopsychosocial dataset that enables examination of links between multiple diseases and pathologies, and strong rapport with Study members and their parents (which promotes honest disclosures about sensitive or stigmatised issues).

Using standardised interview protocols, trained researchers asked Study members and their parents to report the medical and psychiatric histories of their close biological family members: Study members’ siblings, parents, and grandparents. Study members were interviewed at the Dunedin Research Unit when they were aged 32 years; their parents were interviewed in their own home when Study members were 30–33 years old. Family history data were collected on cardiovascular health problems, cancer, arthritis, allergies, asthma, dental health, dementia, substance abuse, and mental health, and are available for 981 Study members.

These comprehensive data have been used to investigate: i) methods of quantifying family disease risk (Milne et al., 2008); ii) the role family history plays in helping to predict risk for, severity of, and outcomes associated with a range of psychopathologies (Milne, Caspi, Crump, et al., 2009; Milne, Caspi, Harrington, et al., 2009; Odgers et al., 2007); iii) factors that contribute to poor health outcomes in children from low socioeconomic backgrounds (Melchior et al., 2007); and iv) links between parent and child oral health (Shearer et al., 2011, 2012). They are also commonly used in Dunedin Study investigations as a control variable where appropriate (e.g., blood pressure trajectories across the life course; Theodore et al., 2015).

***3.2 Parenting Study***

The Parenting Study was designed to investigate how Study members’ upbringing and personal characteristics related to their parenting of their own children. Study members’ parenting was assessed via structured in-home assessments when their first child was around 3 years old. Parent-child interactions were filmed in three semi-structured situations, and standardised scales used to evaluate parental behaviour including sensitive responsiveness, intrusiveness, detachment, stimulation of cognitive development, positive regard for the child, and negative regard for the child. Parenting data were collected from 719 Study members.

Key findings from the Parenting Study include that: i) positive parenting is transmitted intergenerationally, at least for mothers: Study members who received warm parenting were more likely to go on to be warm parents themselves (Belsky et al., 2005); ii) intergenerational transmission of parenting is evident in both younger and older parents (Belsky et al., 2012); iii) psychopathology is associated with less positive parenting (Jaffee et al., 2006); iv) certain genetic factors are associated with more positive parenting, even after taking into account other important psychosocial influences (Wertz et al., 2019); and v) childhood disadvantage has both direct and indirect detrimental effects on positive parenting (McAnally, Iosua et al., 2021).

In addition to the main Parenting Study, we investigated whether relationships between middle-aged parents and their adult children are linked to how the children were parented when they were young. When Study members were 26, they and their parents were separately asked about the nature and quality of their relationship. Responses were obtained from 980 Study members and their parents (905 mothers and 838 fathers). The results showed that more supportive family environments and child-rearing experiences in the family of origin were associated with better quality parent-child relationships in young adulthood (Belsky et al., 2001).

***3.3 Next Generation Study***

The purpose of the Next Generation Study was to understand intergenerational patterns in health and psychosocial outcomes. Study members’ children completed objective and subjective health and wellbeing assessments when they were around 15 years old. Where possible, the assessments were the same or as similar as possible to those completed by their parents at age 15.

Results to date have shown concerning intergenerational shifts in aerobic fitness and weight (decreased fitness and increased weight in the younger generation; McAnally et al., 2018) and stability of the home environment (the younger generation experienced much greater variability in parental care, more changes in household composition, and more address changes; McAnally, Sligo et al., 2021; Sligo et al., 2017). The results have also highlighted patterns in the intergenerational transmission of depression: while recurrent depression in parents (particularly when it starts early in life) was associated with higher rates of depression in their children, a single lifetime episode of depression was not (Jaffee et al., 2021).

***3.4 Dementia Study***

The purpose of the Dementia Study was to collect family history data on dementia symptoms in Study members’ parents and grandparents, with a view to investigating the role such history might play in predicting Study members’ potential risk for dementia as they age. When Study members were around age 45, their parents were asked to report dementia symptoms in themselves, the other parent, and the Study members’ maternal and paternal grandparents. Responses were obtained for 859 Study member mothers, 777 fathers, 833 maternal grandmothers, 808 maternal grandfathers, 750 paternal grandmothers, and 724 paternal grandfathers. Coupled with the comprehensive biopsychosocial data collected throughout Study members’ lives, data from this future-focused study will help us to understand important risk and protective factors for dementia.

***3.5 COVID-19 Vaccine Study***

The purpose of the COVID-19 Vaccine Study was to understand Study members’ attitudes and behaviours in relation to the COVID-19 vaccine. The survey was administered to 832 Study members when they were around 49 years old, via mail or telephone interview. These data will be used to provide insight into the barriers to and facilitators of getting vaccinated (Moffitt et al, 2022).

**References**

Beckley AL, Caspi A, Broadbent J, et al. Association of Childhood Blood Lead Levels With Criminal Offending. *JAMA Pediatr.* 2018;172(2):166–173. doi:10.1001/jamapediatrics.2017.4005

Belsky, J., Hancox, R. J., Sligo, J., & Poulton, R. (2012). Does being an older parent attenuate the intergenerational transmission of parenting? *Developmental Psychology, 48*(6), 1570-1574. doi:10.1037/a0027599

Belsky, J., Jaffee, S., Hsieh, K. H., & Silva, P. A. (2001). Child-rearing antecedents of intergenerational relations in young adulthood: a prospective study. *Developmental Psychology, 37*(6), 801-813. doi:10.1037//0012-1649.37.6.801

Belsky, J., Jaffee, S. R., Sligo, J., Woodward, L., & Silva, P. A. (2005). Intergenerational transmission of warm-sensitive-stimulating parenting: a prospective study of mothers and fathers of 3-year-olds. *Child Dev, 76*(2), 384-396. doi:10.1111/j.1467-8624.2005.00852.x

Jaffee, S. R., Belsky, J., Harrington, H., Caspi, A., & Moffitt, T. E. (2006). When parents have a history of conduct disorder: how is the caregiving environment affected? *J Abnorm Psychol, 115*(2), 309-319. doi:10.1037/0021-843x.115.2.309

Jaffee, S. R., Sligo, J. L., McAnally, H. M., Bolton, A. E., Baxter, J. M., & Hancox, R. J. (2021). Early-onset and recurrent depression in parents increases risk of intergenerational transmission to adolescent offspring. *J Child Psychol Psychiatry, 62*(8), 979-988. doi:10.1111/jcpp.13356

McAnally, H. M., Iosua, E., Sligo, J. L., Belsky, J., Spry, E., Letcher, P., . . . Hancox, R. J. (2021). Childhood disadvantage and adolescent socioemotional wellbeing as predictors of future parenting behaviour. *J Adolesc, 86*, 90-100. doi:10.1016/j.adolescence.2020.12.005

McAnally, H. M., Reeves, L. M., Sligo, J. L., & Hancox, R. J. (2018). Intergenerational changes in adolescents' physical fitness and weight in New Zealand. *New Zealand Medical Journal, 131*(1482), 16-28.

McAnally, H. M., Sligo, J. L., Baxter, J., Tansley, J. E., Bolton, A. E., & Hancox, R. J. (2021). Changes to family structure, household composition and address among young New Zealanders: an update. *Kōtuitui: New Zealand Journal of Social Sciences Online*, 1-12. doi:10.1080/1177083X.2021.1957946

Melchior, M., Moffitt, T. E., Milne, B. J., Poulton, R., & Caspi, A. (2007). Why do children from socioeconomically disadvantaged families suffer from poor health when they reach adulthood? A life-course study. *Am J Epidemiol, 166*(8), 966-974. doi:10.1093/aje/kwm155

Milne, B. J., Caspi, A., Crump, R., Poulton, R., Rutter, M., Sears, M. R., & Moffitt, T. E. (2009). The validity of the family history screen for assessing family history of mental disorders. *Am J Med Genet B Neuropsychiatr Genet, 150b*(1), 41-49. doi:10.1002/ajmg.b.30764

Milne, B. J., Caspi, A., Harrington, H., Poulton, R., Rutter, M., & Moffitt, T. E. (2009). Predictive value of family history on severity of illness: the case for depression, anxiety, alcohol dependence, and drug dependence. *Arch Gen Psychiatry, 66*(7), 738-747. doi:10.1001/archgenpsychiatry.2009.55

Milne, B. J., Moffitt, T. E., Crump, R., Poulton, R., Rutter, M., Sears, M. R., . . . Caspi, A. (2008). How should we construct psychiatric family history scores? A comparison of alternative approaches from the Dunedin Family Health History Study. *Psychol Med, 38*(12), 1793-1802. doi:10.1017/s0033291708003115

Moffitt TE, Caspi A, Ambler A, Bourassa K, Harrington H, Hogan S, Houts R, Ramrakha S, Wood SL and Poulton R. 2022. Deep-seated psychological histories of COVID-19 vaccine hesitance and resistance. *PNAS Nexus, 0,* 1-11. doi.org/10.1093/pnasnexus/pgac034

Odgers, C. L., Milne, B. J., Caspi, A., Crump, R., Poulton, R., & Moffitt, T. E. (2007). Predicting prognosis for the conduct-problem boy: can family history help? *J Am Acad Child Adolesc Psychiatry, 46*(10), 1240-1249. doi:10.1097/chi.0b013e31813c6c8d

Reuben A, Caspi A, Belsky DW, et al. Association of Childhood Blood Lead Levels With Cognitive Function and Socioeconomic Status at Age 38 Years and With IQ Change and Socioeconomic Mobility Between Childhood and Adulthood. JAMA. 2017;317(12):1244-1251. doi:10.1001/jama.2017.1712

Reuben A, Elliott ML, Abraham WC, et al. Association of Childhood Lead Exposure With MRI Measurements of Structural Brain Integrity in Midlife. *JAMA.* 2020;324(19):1970–1979. doi:10.1001/jama.2020.19998

Reuben A, Schaefer JD, Moffitt TE, et al. Association of Childhood Lead Exposure With Adult Personality Traits and Lifelong Mental Health. JAMA Psychiatry. 2019;76(4):418-425. doi:10.1001/jamapsychiatry.2018.4192

Shearer, D. M., Thomson, W. M., Caspi, A., Moffitt, T. E., Broadbent, J. M., & Poulton, R. (2011). Inter-generational continuity in periodontal health: findings from the Dunedin family history study. *J Clin Periodontol, 38*(4), 301-309. doi:10.1111/j.1600-051X.2011.01704.x

Shearer, D. M., Thomson, W. M., Caspi, A., Moffitt, T. E., Broadbent, J. M., & Poulton, R. (2012). Family history and oral health: findings from the Dunedin Study. *Community Dent Oral Epidemiol, 40*(2), 105-115. doi:10.1111/j.1600-0528.2011.00641.x

Sligo, J. L., McAnally, H. M., Tansley, J. E., Baxter, J. M., Bolton, A. E., Skillander, K. M., & Hancox, R. J. (2017). The dynamic, complex and diverse living and care arrangements of young New Zealanders: implications for policy. *Kōtuitui: New Zealand Journal of Social Sciences Online, 12*(1), 41-55. doi:10.1080/1177083X.2016.1196715

Theodore, R. F., Broadbent, J., Nagin, D., Ambler, A., Hogan, S., Ramrakha, S., . . . Poulton, R. (2015). Childhood to Early-Midlife Systolic Blood Pressure Trajectories: Early-Life Predictors, Effect Modifiers, and Adult Cardiovascular Outcomes. *Hypertension, 66*(6), 1108-1115. doi:10.1161/hypertensionaha.115.05831

Wertz, J., Belsky, J., Moffitt, T. E., Belsky, D. W., Harrington, H., Avinun, R., . . . Caspi, A. (2019). Genetics of nurture: A test of the hypothesis that parents' genetics predict their observed caregiving. *Developmental Psychology, 55*(7), 1461-1472. doi:10.1037/dev0000709
